# Nonsense-mediated mRNA decay inhibition reshapes the cancer immunopeptidome

**DOI:** 10.1016/j.immuni.2026.02.005

**Published:** 2026-04-08

**Authors:** Roberto Vendramin, Hongchang Fu, Shanila Fernandez Patel, Yue Zhao, Danwen Qian, Lorena Ligammari, Osnat Bartok, Polina Greenberg, Ronen Levy, Andrea Castro, Krupa Thakkar, Jun Murai, Wei-Ting Lu, Christopher C.T. Sng, Chen Weller, Gordon Beattie, Amandeep Bhamra, Roc Farriol-Duran, Despoina Karagianni, Marcellus Augustine, Krijn K. Dijkstra, Christopher L. Pinder, Benjamin S. Simpson, Gordon Weng-Kit Cheung, TRACERx Consortium, Felipe Galvez-Cancino, Petra Vlckova, Silvia Surinova, Manuel Rodriguez-Justo, Mansi Shah, Nicholas McGranahan, Jeremy G. Carlton, Eva Grönroos, James L. Reading, Yardena Samuels, Charles Swanton, Sergio A. Quezada, Kevin Litchfield

**Affiliations:** 1 The Tumor Immunogenomics and Immunosurveillance Lab, University College London Cancer Institute, London, UK; 2 Cancer Evolution and Genome Instability Lab, The Francis Crick Institute, London, UK; 3 CRUK Lung Cancer Centre of Excellence, University College London Cancer Institute, London, UK; 4 Pre-Cancer Immunology Lab, University College London Cancer Institute, London, UK; 5 Organelle Dynamics Lab, School of Cancer & Pharmaceutical Sciences, King’s College London, London, UK; 6 Organelle Dynamics Lab, the Francis Crick Institute, London, UK; 7 Department of Thoracic Surgery, Shanghai Chest Hospital, Shanghai Jiao Tong University School of Medicine, Shanghai, China; 8 Department of Molecular Cell Biology, Weizmann Institute of Science, Rehovot, Israel; 9 Drug Discovery Technology Laboratories, Ono Pharmaceutical Co. Ltd., Osaka, Japan; 10 Department of Oncology, Medical Sciences Division, University of Oxford, Oxford, UK; 11 CRUK City of London Centre Single Cell Genomics Facility, University College London Cancer Institute, Oxford, UK; 12 Bioinformatics Hub, University College London Cancer Institute, London, UK; 13 Proteomics Research Translational Technology Platform, University College London Cancer Institute, London, UK; 14 Barcelona Supercomputing Center (BSC), Barcelona, Spain; 15 Cancer Genome Evolution Research Group, University College London Cancer Institute, London, UK; 16 Immune Regulation and Tumor Immunotherapy Group, University College London Cancer Institute, London, UK; 17 Division of Medicine, University College London, London, UK; 18 Department of Molecular Oncology and Immunology, the Netherlands Cancer Institute, Amsterdam, the Netherlands; 19 Oncode Institute, Utrecht, the Netherlands; 20 Research Department of Haematology, University College London Cancer Institute, London, UK; 21 Immune Regulation Lab, Centre for Immuno-Oncology, Nuffield Department of Medicine, University of Oxford, Oxford, UK; 22 Organoid Translational Technology Platform, University College London Cancer Institute, London, UK; 23 Department of Research Pathology, University College London Cancer Institute, London, UK; 24 CRUK City of London Explant and Patient-Derived Xenograft Core, London, UK

## Abstract

DNA mutations are a well-characterized source of neoepitopes in immunotherapy. Here, we examined the contribution of dysregulated RNA processing to neoantigen production. Leveraging multi-omics and check-point inhibitor (CPI) response data from >1,000 patients, we identified reduced activity of the nonsense-mediated mRNA decay (NMD) pathway kinase SMG1 as a predictor of improved CPI response. NMD inhibition through SMG1 targeting stabilized transcripts containing premature termination codons, most of which were of non-mutational origin. This reshaped the major histocompatibility complex class I (MHC class I)-bound immunopeptidome and increased neoantigen abundance to levels comparable to high mutation burden tumors. Functionally, NMD inhibition drove antigen-dependent T cell-mediated tumor cell killing *in vitro*, promoted activation of tissue-resident T cells in patient-derived models *ex vivo*, and improved CPI efficacy *in vivo*. Our findings establish NMD inhibition as a strategy to harness a previously inaccessible source of canonical and non-canonical neoantigens, with the potential to increase tumor immunogenicity across cancers.

## Graphical abstract

**Graphical abstract:**
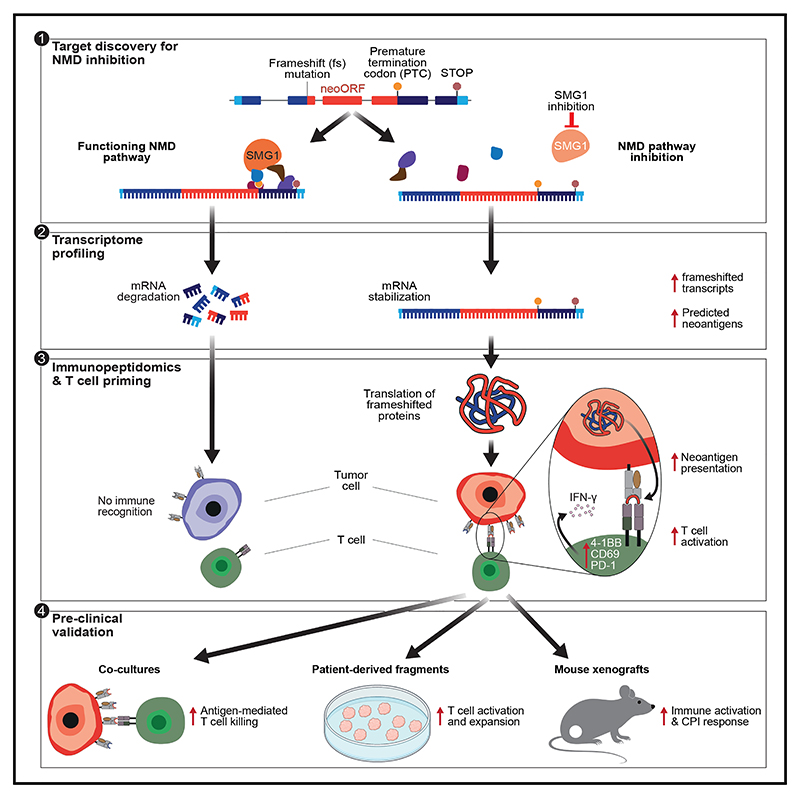

## Introduction

Effective anti-tumor immunity relies on the ability of T cells to detect cancer-associated peptides presented by the major histocompatibility complex (MHC) molecules and is shaped by both the quantity and quality of neoantigens.^1,2^ Among these, mutation-derived neoantigens (peptides derived from tumor-specific genetic alterations) represent key targets for immune recognition and elimination. High tumor mutational burden (TMB-high) is therefore a strong predictor of immunotherapy response, yet only ~10% of human tumors fall into this category.^3–12^ Moreover, the majority of somatic alterations are missense mutations that often elicit weak immunogenicity, as they result in at most a single amino acid change in the peptide sequence.^13–17^ Therefore, identifying additional sources of potent neoantigens, including those arising from non-mutational processes, is crucial to promote anti-tumor immunity, particularly in TMB-low settings.^10,18^

Notably, many solid tumors harbor abundant tumor-infiltrating lymphocytes (TILs) that nonetheless fail to mediate durable tumor control, reflecting a state of functional exhaustion.^19–21^ These TILs frequently undergo oligoclonal expansion, yet their antigen specificities often remain unknown.^22–24^ Together, these observations suggest that additional immunogenic tumor antigens may exist but remain insufficiently generated or presented to sustain effective T cell-mediated tumor control. We hypothesized that a critical mechanism limiting the production of these antigens is the nonsense-mediated mRNA decay (NMD) pathway. NMD detects and degrades transcripts containing premature termination codons (PTCs),^25^ including those arising from frameshift insertions/deletions (fs-indels) and intron-retention mutations.^25^ This effectively eliminates a rare yet highly immunogenic source of antigens, as these aberrant transcripts can encode long, non-self peptide sequences.^9,26–36^ A small subset of these transcripts can nonetheless escape degradation, and their abundance correlates with increased CD8^+^ T cell infiltration and improved responses to checkpoint inhibitors (CPIs).^9,26–28,37–40^ However, because NMD escape is rare, many potentially immunogenic neoantigens are likely never generated. Moreover, NMD-escape variants, unlike their NMD-targeted counterparts, exhibit clear signatures of negative selection, indicating that their immunogenicity imposes a selective pressure that limits their occurrence in human cancers.^27,28,38^ These findings suggest that NMD-mediated transcript degradation acts as a key immune-evasion mechanism by selectively eliminating transcripts encoding potent neoantigens. Beyond PTC-generating fs-indels and intron-retention mutations, NMD regulates a wide array of aberrant PTC-containing transcripts that are often dysregulated in cancer. These include incorrectly spliced mRNAs, pseudogene-derived RNAs, long non-coding RNAs (lncRNAs), and those originating from alternative/cryptic open reading frames (ORFs).^34,41–47^ These transcripts can provide an abundant source of non-canonical, non-mutational neoantigens. Accordingly, preclinical and clinical data indicate that the resulting neoepitopes can elicit potent T cell responses and have a major impact on disease progression and CPI efficacy.^17,47–57^ Consistent with this immunoregulatory role, tumors often exhibit elevated NMD activity, which potentially enables cancer cells to limit the accumulation of such aberrant RNAs and evade immune pressure.^37,38,39^ Accordingly, genetic ablation of NMD pathway components has been shown to suppress tumor growth *in vivo*, in an immune-dependent manner.^58,59^ However, the molecular mechanisms that drive these immune effects, and their relevance to human cancer, remain underexplored. Moreover, although the immunogenic potential of peptides deriving from NMD-escape transcripts has been previously reported,^9,26–28,37–40^ direct evidence that stabilizing transcripts normally targeted by NMD can produce immunogenic neoantigens and elicit bona fide anti-tumor immune responses is still lacking.

In this study, we address these key questions by determining whether NMD pathway inhibition (NMDi) in cancer can stabilize aberrant NMD-targeted PTC-containing transcripts and, critically, whether this previously inaccessible neoantigenic source becomes detectable in the immunopeptidome. We further investigate the immunological consequence of this expanded antigenic landscape, specifically whether their abundance is sufficient to support T cell recognition, activation, cytotoxicity, and clonal expansion.

## Results

### Systematic prioritization of NMD pathway components linked to anti-tumor immunity

**Figure 1:**
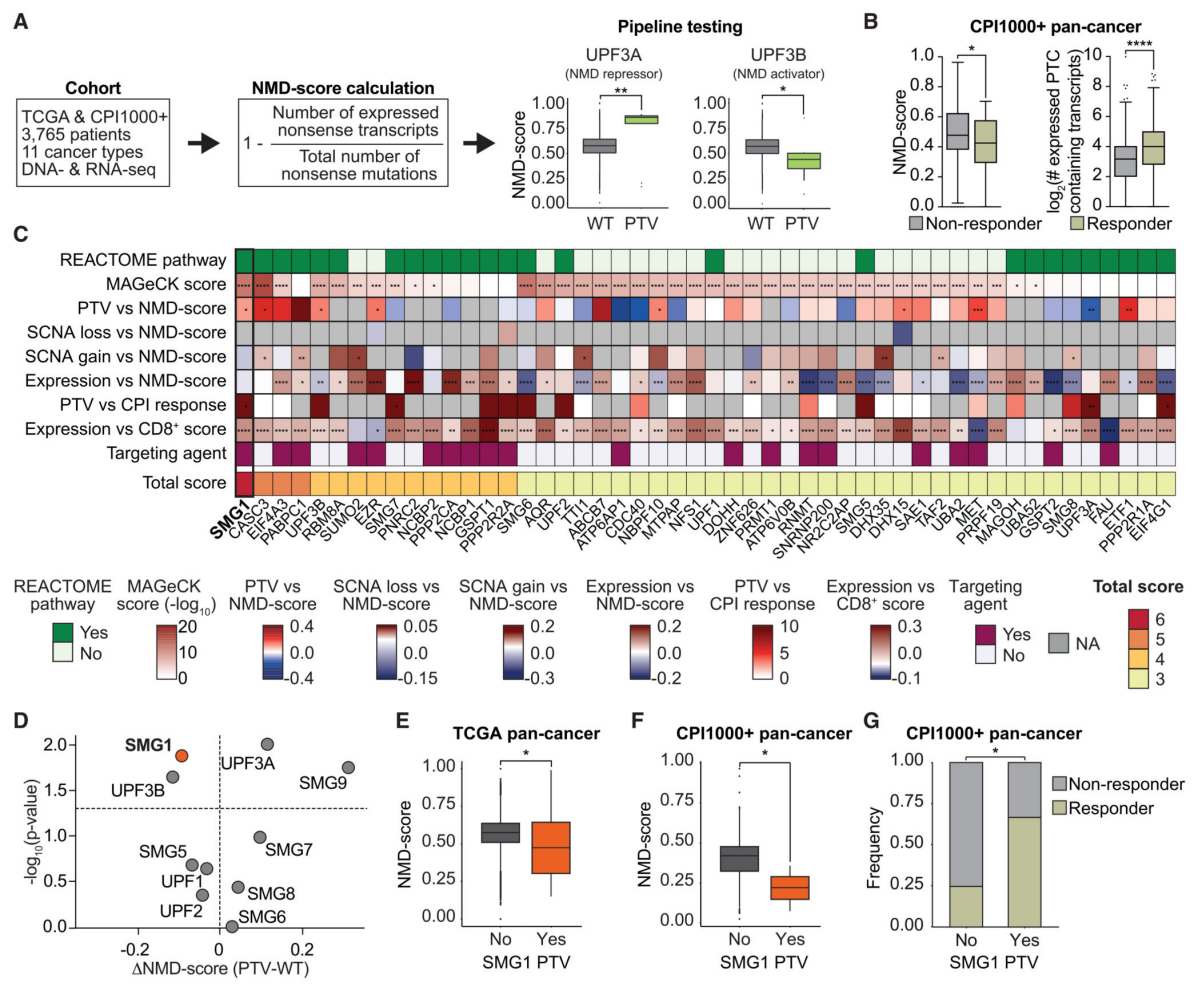
Identification of SMG1 as a candidate for cancer immunotherapy (A) Patient cohort and NMD-score formula (left) and NMD-score in tumor samples (TCGA pan-cancer) stratified by wild-type (WT) or protein-truncating variant (PTV) mutation in *UPF3A* or *UPF3B* (right, two-tailed Mann-Whitney U test). (B) Association between CPI response and either NMD score (left) or the number of expressed PTC-containing transcripts (right) in the CPI1000+ cohort (Student’s *t* test with a multivariate linear model adjusted for tumor type and TMB). (C) Heatmap of parameters used to rank NMD pathway genes. Genes with a summed score ≥3 are shown. (D) Impact of PTVs in NMD core genes on the NMD score. The *x* axis shows the ΔNMD score, calculated by subtracting the mean NMD score of WT tumors for a given NMD core gene from that of tumors harboring a PTV in the same gene. The *y* axis shows −log_10_(*p* value) (two-tailed Mann-Whitney U test). (D) Association between NMD score and *SMG1* mutational status (WT, *n* = 4,208; PTV, *n* = 26) in the TCGA pan-cancer cohort (two-tailed Mann-Whitney U test). (F and G) Association between SMG1 mutational status (WT, *n* = 1,163; PTV, *n* = 6) and either NMD score (F; two-tailed Mann-Whitney U test) or CPI response (G; Fisher’s exact test) in the CPI1000+ pan-cancer cohort. For boxplots, boxes denote the interquartile range, the black line represents the median, and whiskers extend to 1.5× the interquartile range; *p ≤ 0.05, **p ≤ 0.01, ***p ≤ 0.001, ****p ≤ 0.0001. See also Figures S1 and S2 and Table S1.

The NMD pathway has been associated with tumor immunogenicity and CPI responses.^9,26–28,37–40^ However, the relative functional contributions of the multiple genes involved in NMD remain unclear, and the association of individual NMD components with anti-tumor immune activity and CPI responses has not been systematically evaluated. To address this gap, we performed a multi-layered, orthogonal prioritization of NMD pathway components by integrating multiple data types, including genetics, transcriptomics, immune infiltration, and clinical CPI response, across multiple clinical datasets. We first compiled a comprehensive dataset of somatic and germline PTC-generating nonsense mutations using matched DNA and RNA sequencing (RNA-seq) data from 3,765 patients across 11 cancer types from The Cancer Genome Atlas (TCGA) and CPI1000+ cohorts.^9^ We then used transcriptomic data to confidently identify all genomic nonsense mutations subjected to NMD, yielding >35,000 validated events. Next, to quantify NMD pathway activity at a sample level, we developed a metric termed “NMD score,” defined as the proportion of expressed PTC-containing transcripts among all PTC-inducing mutations detected at the DNA and/or RNA level, with lower values reflecting weaker and higher values stronger NMD activity, respectively (Figure 1A). Analysis of truncating mutations (protein-truncating variants [PTVs]) in paralog genes with opposing effects on NMD (UPF3A regulator of nonsense mediated mRNA decay [*UPF3A*], NMD repressor; UPF3B regulator of nonsense mediated mRNA decay [*UPF3B*], NMD activator) confirmed that the NMD score accurately discriminated between pathway activators and repressors (Figure 1A). The NMD score thus provided a means to relate pathway activity to the expression, or mutational status, of individual genes in clinical samples.

We next sought to assess whether NMD pathway activity correlated with response to CPI by applying the NMD score to the CPI1000+ cohort.^9^ Lower NMD scores (or higher expression of PTC-containing transcripts) were associated with improved responses (either complete or partial) to CPI (Figures 1B and S1A), independent of TMB or tumor type. Moreover, PTVs in one or more NMD pathway components correlated with improved clinical responses to CPIs (Figure S1B). Additionally, the expression of NMD-related genes was elevated in tumors (from TCGA) compared with normal tissues (from the Genotype-Tissue Expression [GTEx]), suggesting that increased NMD activity is associated with tumor progression (Figure S1C).^37,40,60^

Next, we set out to identify the NMD pathway component with the most central relevance to pathway activity as well as the strongest association with increased CD8^+^ T cell infiltration and improved CPI responses. Toward that end, we compiled an extensive, curated list of NMD pathway genes by combining those annotated in the peer-reviewed Reactome pathway database (R-HSA-927802)^61^ with genes identified in a recent genome-wide NMD CRISPR screen,^62^ yielding 189 unique candidates. We next developed a bioinformatics pipeline and scoring system to rank NMD-associated genes based on nine parameters capturing NMD pathway relevance: (1) presence in the Reactome pathway database,^61^ (2) significant MAGeCK (model-based analysis of genome-wide CRISPR-Cas9 knockout) score in a recent NMD CRISPR screen,^62^ (3) association with NMD score across PTVs, (4) somatic copy-number alteration (SCNA) loss, (5) SCNA gain, (6) expression analyses, (7) association with response to CPI, (8) association with CD8^+^ T cell infiltration, and (9) availability of therapeutic agents targeting the protein product of the gene (see STAR Methods). Genes were then ranked based on their cumulative scores, and candidates with a score ≥3 were selected for further consideration (Figure 1C). The SMG1 nonsense mediated mRNA decay associated PI3K related kinase (SMG1), a key kinase that initiates the process of NMD mRNA degradation by phosphorylating the UPF1 RNA helicase and ATPase (UPF1),^63–65^ emerged as the top-scoring candidate. *SMG1* PTVs caused a marked reduction in NMD activity (Figures 1C–1F), consistent with prior studies identifying SMG1-mediated phosphorylation of UPF1 as the rate-limiting step in NMD,^63,66^ and were associated with improved CPI responses (Figure 1G). Finally, SMG1 expression was found to be elevated in tumors compared with normal tissues and negatively correlated with CD8^+^ T cell infiltration (Figures 1C and S1C).

We next sought to validate SMG1 as a target by confirming its functional relevance experimentally. We first generated *SMG1* knockout melanoma cells (A375) using CRISPR-Cas9 and observed a complete loss of UPF1 phosphorylation, consistent with the established role of SMG1 as the central kinase responsible for UPF1 phosphorylation (Figure S1D). We then assessed the impact of partial SMG1 suppression on NMD activity by downregulating SMG1 in melanoma (A375) and lung adenocarcinoma (LUAD; PC-9) cells using SMG1-targeting small interfering RNAs (siRNAs) (siSMG1) or two distinct SMG1 small-molecule inhibitors (SMG1i).^67–70^ Both approaches led to a marked reduction in phosphorylation of UPF1 (pUPF1), confirming effective inhibition of SMG1 activity (Figures S1E–S1H). Enzyme-linked immunosorbent assay (ELISA)-based quantification of pUPF1 showed potent activity for both inhibitors (SMG1i#1 and SMG1i#2), with IC_50_ values in the nanomolar range (Figure S1I). Experiments were performed using concentrations that achieved robust (75%) SMG1 inhibition without detectable cytotoxicity (0.5 μM [SMG1i#1] and 1 μM [SMG1i#2]) (Figures S1I and S1J). Similarly, siRNA-mediated knockdown of SMG1 did not affect cell growth (Figure S1K). Long-term treatment with either SMG1 inhibitor resulted in sustained suppression of UPF1 phosphorylation for up to 72 h, with a gradual recovery of pUPF1 to baseline levels at later time points (Figures S1L and S1M).

Next, we generated stable cell lines expressing a fluorescent NMD reporter encoding an enhanced green fluorescent protein (EGFP) mRNA engineered to contain a PTC, making it an NMD target and enabling fluorescence to function as an inverse measure of NMD activity (Figure S1N).^62^ SMG1 knockdown or inhibition led to a marked increase in reporter fluorescence relative to controls, indicating effective NMD suppression (Figures S1O and S1P). As SMG1 is a member of the phosphatidylinositol 3-kinase-related kinase (PIKK) family, we assessed the reporter specificity by targeting another PIKK, mammalian target of rapamycin (mTOR), using rapamycin.^71^ We also inhibited splicing with indisulam,^52^ given the known link between splicing and NMD.^72^ Neither treatment altered reporter fluorescence, confirming its specificity for NMD pathway activity (Figure S1Q).

To identify endogenous PTC-containing transcripts regulated by NMD across tumor types, we knocked down UPF1, a master regulator of NMD, in six tumor cell lines. RNA-seq and isoform-level analysis revealed widespread upregulation of PTC-containing transcripts (Table S1). Four PTC-containing transcripts originating from alternatively spliced variants of protein-coding genes (DNAJC25-PTC, TBRG1-PTC, TMEM208-PTC, and U2AF1-PTC) were consistently upregulated across all cell lines and selected as putative markers of NMD activity (Figure S2A; Table S1). Importantly, direct comparison of SMG1 and UPF1 knockdown in A375 cells revealed highly concordant transcriptional responses, with both perturbations increasing the expression of canonical NMD-target genes (ATF3, ATF4, and GADD45A) as well as the selected PTC-containing transcripts, consistent with similar disruption of NMD activity (Figure S2B). Accordingly, SMG1 inhibition or knock-down in both melanoma and LUAD cell lines robustly induced the expression of these PTC-containing transcripts (Figures S2C and S2D), along with that of canonical NMD-target genes (ATF3, ATF4, GADD45A, GADD45B, and GAS5; Figures S2E and S2F), while mTOR or splicing inhibition had no effect (Figure S2G).

Collectively, these findings establish SMG1 as a central node of the NMD pathway, whose activity is associated with tumor immune phenotypes in clinical datasets and whose impairment potently inhibits NMD, thereby stabilizing putative NMD-target transcripts.

### NMDi triggers *ex vivo* tumor-specific activation and clonal expansion of tissue-resident T cells

**Figure 2:**
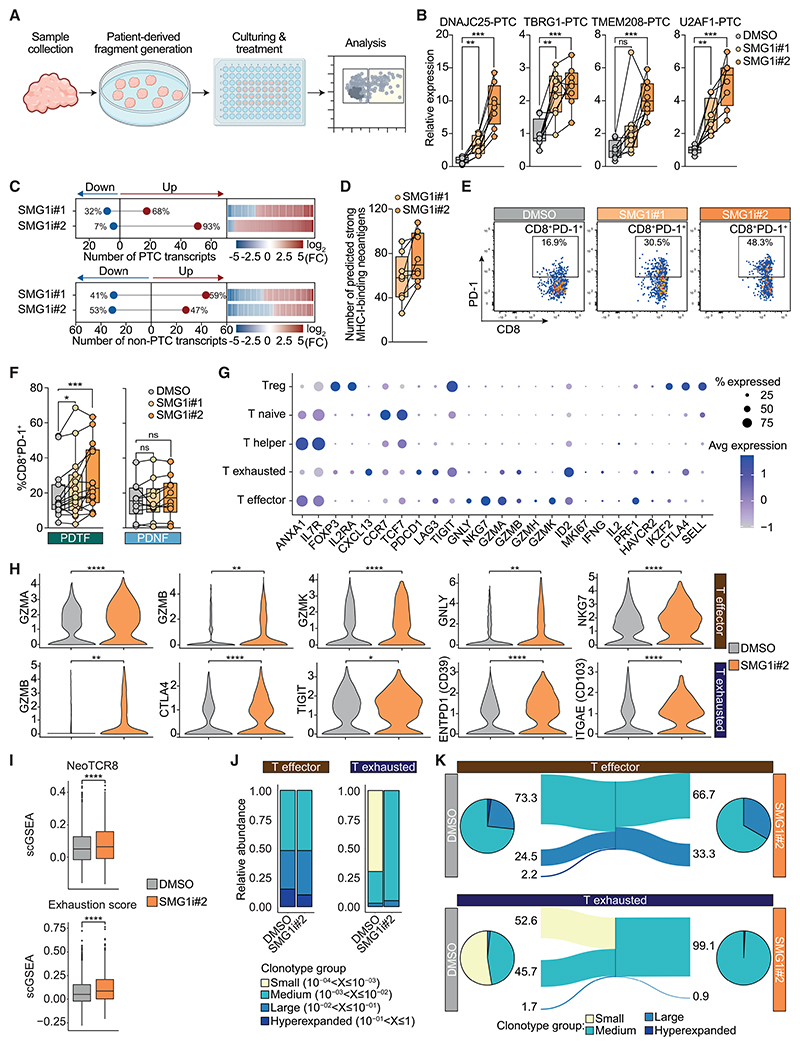
SMG1i activates tumor-specific T cells in human patient-derived fragments (PDFs) (A) Schematic of the workflow for PDFs. (B) Relative expression of PTC-containing transcripts measured by RNA-seq in PDTFs 24 h after treatment with SMG1i#1 or SMG1i#2 relative to DMSO controls (one-way repeated-measures ANOVA with Holm-Šidák correction; *n* = 8 patients). (C) (Left) Lollipop plots displaying the number of significantly (false discovery rate [FDR]-adjusted *p* value [*q*] < 0.05, relative isoform fraction [rIF] > 1.5) upregulated and downregulated PTC-containing transcripts (top) or non-PTC-containing transcripts (bottom), and (right) corresponding heatmaps showing log_2_ fold change (FC) relative to DMSO of PDTFs described in (B). (D) Number of predicted strong MHC class I binding neoantigens derived from the *in silico* translation of significantly upregulated PTC-containing transcripts described in (C) (*n* = 8 patients, see STAR Methods). (E) Representative flow cytometry plots of CD8^+^PD-1^+^ T cells in PDTFs 96 h after treatment with SMG1i#1, SMG1i#2, or DMSO. (F) PD-1 surface expression (% of CD8^+^ T cells) in PDTFs (left, *n* = 15 patients) and in matched patient-derived normal tissue adjacent to the tumor (NAT) (PDNFs) (right, *n* = 10 patients) 96 h after treatment with SMG1i#1, SMG1i#2, or DMSO (one-way repeated-measures ANOVA with Holm-Šidák correction). (G) Bubble plot showing normalized expression of key T cell marker genes across T cells from scRNA-seq of PDTFs. Bubble size indicates the percentage of cells expressing each gene, while color intensity reflects the average expression level among expressing cells. (H) Normalized gene expression of activation markers in the T effector population (top) and exhaustion markers in the T exhausted population (bottom) from (G) (two-tailed Mann-Whitney U test). (I) Average gene signature scores (scGSEA) of the NeoTCR8 score^82^ (top) and T cell exhaustion score^83^ (bottom) in effector and exhausted T cells from (G) (two-tailed Mann-Whitney U test). (J) Relative abundance of all TCR clonotypes in the effector and exhausted T cell populations from (G). (K) Pie charts and Sankey plots showing the relative abundance (%) of TCR clonotypes shared between the DMSO and SMG1i#2 conditions within the effector and exhausted T cell populations from (G). For boxplots, boxes denote the interquartile range, the black line represents the median, and whiskers extend to 1.5× the interquartile range. A line connects dots corresponding to the same patient across treatments; ns, *p* > 0.05, *p ≤ 0.05, **p ≤ 0.01, ***p ≤ 0.001, ****p ≤ 0.0001. See also Figure S3 and Tables S2, S3, and S4.

We next tested whether SMG1i could elicit immunological responses in a clinically relevant setting. To this end, we employed patient-derived tumor fragments (PDTFs) (Figure 2A), which, unlike conventional cell lines or murine models, preserve the native tissue architecture, cellular composition, and immune environment of patient tumors.^73,74^ Importantly, previous studies have shown that this model closely reflects clinical responses to immunotherapy and that the observed immunological responsiveness is influenced, at least in part, by pre-existing intra-tumoral T cells.^73,74^ The cohort was composed primarily (80%) of mismatch repair-proficient (pMMR), microsatellite stable (MSS) colorectal cancer (CRC) samples (Table S2),^75^ a tumor type characterized by low-TMB and poor CPI responses.^76^ Fragments were treated with either DMSO or SMG1 inhibitors, then subjected to bulk RNA-seq to assess transcriptomic changes upon NMDi. Because many NMD-targeted transcripts arise through alternative splicing, gene-level analyses alone cannot capture the full complexity of NMD-dependent transcript dynamics.^77–79^ We therefore performed differential gene- and iso-form-level expression analysis to accurately detect and quantify PTC-containing transcripts upon NMD perturbation (Figure S3A, see STAR Methods). This analysis confirmed effective *ex vivo* NMDi following SMG1i treatment, with robust upregulation of both PTC-containing transcripts and canonical NMD targets (Figures 2B, 2C, and S3B). Importantly, many of the significantly upregulated transcripts encoded potential neo open reading frames (neoORFs), suggesting that they may be translated into neoepitopes.^52,53,80,81^ Consistent with this possibility, *in silico* translation and MHC class I binding prediction identified numerous high-affinity neoantigens originating from these transcripts, suggesting that SMG1i may expand the cellular neoantigen repertoire (Figure 2D).

To directly assess whether NMD inhibition induces tumor-specific immune activation in this setting, we next performed high-dimensional flow cytometry (Figure S3C). SMG1i activated intratumoral CD4^+^ and CD8^+^ T cells, as indicated by elevated programmed death-1 (PD-1, CD279) surface expression (Figures 2E, 2F, and S3D),^84–86^ and led to increased interferon-gamma (IFN-γ) secretion (Figure S3E). Importantly, this was not observed in matched normal adjacent tissue (NAT) patient-derived normal fragments (PDNFs) (Figures 2F, S3D, and S3E). Phenotypic profiling showed that the increase in PD-1^+^ was largely confined to the effector memory (T_EM_) subset in both CD4^+^ and CD8^+^ T cells (Figures S3F–S3I), consistent with the phenotype of tumor-reactive and exhausted T cells in solid cancers.^87^ Together, these results suggest that SMG1 inhibition activates tissue-resident, antigen-experienced T cells in a tumor-specific manner, driving local recall responses.^88–91^

To further dissect the immunological response, we performed single-cell RNA (scRNA) and T cell receptor (TCR)-sequencing (TCR-seq) following DMSO or SMG1i treatment, allowing simultaneous identification of transcriptional states and clonal relationships across T cells (Figures 2G and S3J; Table S3). SMG1i induced upregulation of cytotoxic genes within both effector and exhausted T cell clusters and increased NeoTCR8 and exhaustion scores, consistent with (neo)antigen-driven T cell activation (Figures 2H and 2I).^82,83^ Clonotype analysis revealed clonal expansion within the exhausted compartment (Figures 2J and 2K), characterized by an increase in medium-sized clones at the expense of smaller ones, consistent with antigen-driven T cell expansion. Interestingly, clonal expansion was not observed in the cluster of non-exhausted (*PDCD1*^low^) cytotoxic memory cells, which are considered to represent the bystander, often pathogen-specific, not tumor-specific TIL population.^92^

Together, these data show that SMG1i promotes tumor-specific *ex vivo* activation and clonal expansion of tissue-resident, antigen-experienced T cells, driving recall-like responses in PDTFs derived from TMB-low, immune-cold tumors.

### NMDi enhances CPI efficacy

We next assessed whether SMG1i could enhance CPI efficacy *in vivo*. Due to the limited solubility of the compounds used for the *in vitro* and *ex vivo* experiments, we used KVS0001, a recently developed *in vivo*-compatible derivative of SMG1i#1.^93^ KVS0001 treatment of PC-9 cells increased expression of PTC-containing transcripts, canonical NMD targets, and reporter fluorescence, confirming pathway inhibition (Figures S4A and S4B).

**Figure 3:**
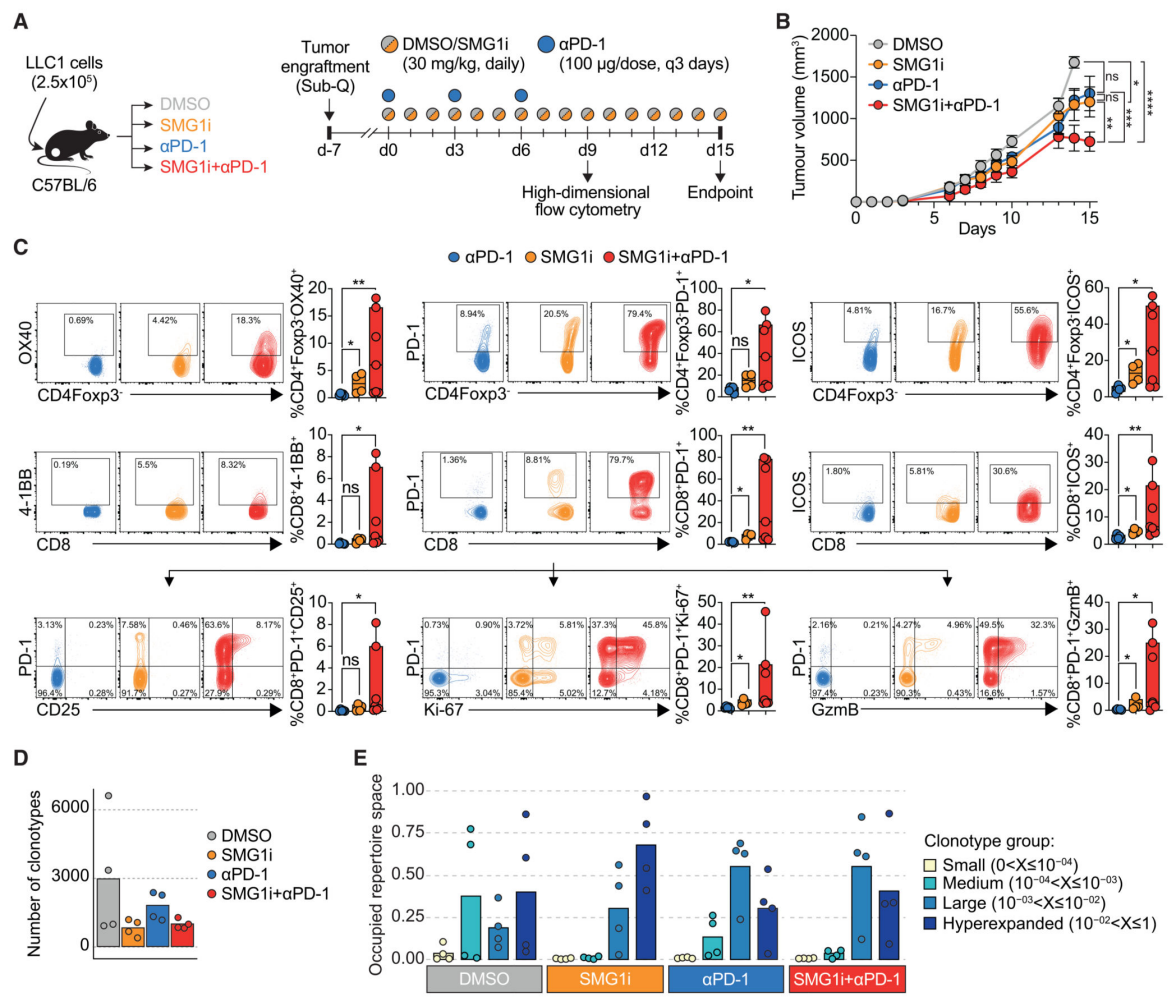
SMG1 inhibition potentiates tumor immunogenicity and CPI efficacy *in vivo* by enhancing T cell responses (A) Schematic representation of the experimental setup and treatment regimen for C57BL/6 mice bearing LLC1 tumors. (B) Tumor growth curves for mice described in (A), treated with DMSO (*n* = 9 biological replicates), KVS0001 (SMG1i; *n* = 7 biological replicates), αPD-1 (*n* = 7 biological replicates), or SMG1i+αPD-1 (*n* = 8 biological replicates) (two-way ANOVA with Tukey’s multiple comparisons test). (C) Representative flow cytometry plots (left) and quantification (right) of T cells following treatment with SMG1i (*n* = 4 biological replicates), αPD-1 (*n* = 5 biological replicates), and SMG1i+αPD-1 (*n* = 7 biological replicates) (two-tailed Mann-Whitney U test). (D) Number of unique T cell clonotypes identified by bulk TCR-seq of tumors across all treatment groups (*n* = 4 biological replicates). (E) Analysis of the relative abundance of TCRs in tumors as described in (D). Bar plots represent the mean ± SEM. For boxplots, boxes indicate the interquartile range, the black line represents the median, and whiskers extend to 1.5× the interquartile range; ns, *p* > 0.05, *p ≤ 0.05, **p ≤ 0.01, ***p ≤ 0.001, ****p ≤ 0.0001. See also Figure S4.

For *in vivo* studies, we used the LLC1 lung cancer model (also known as LL/2 or 3LL), which originates from a spontaneous Lewis lung carcinoma and harbors a high fs-indel burden. LLC1 tumors are highly aggressive and refractory to CPI owing to a profoundly immunosuppressive tumor microenvironment, making this a stringent model for testing novel immuno-oncology strategies.^94–97^ KVS0001 treatment upregulated canonical NMD-target transcripts in LLC1 cells *in vitro* (Figure S4C).^93^ LLC1 cells were injected subcutaneously into C57BL/6 mice, and animals were randomized on day 7 to receive vehicle (DMSO), KVS0001 (SMG1i), anti-PD-1 (αPD-1), or KVS0001 plus αPD-1 (SMG1i+αPD-1) (Figure 3A). KVS0001 monotherapy suppressed tumor growth comparably to αPD-1, while the combination induced greater tumor control than either treatment alone (Figure 3B). Importantly, no overt toxicity was observed aside from transient, mild weight loss (Figure S4D).

We next investigated the immunological basis of the increased efficiency observed upon the combination of SMG1i and αPD-1. To this end, we performed high-dimensional flow cytometry on day 9 post-treatment, 3 days after the final αPD-1 dose, to capture the early immunological changes preceding tumor immune escape across all treatment conditions. Despite the myeloid-dominated LLC1 TME (Figures S4E and S4F),^98^ which likely contributes to the intrinsic CPI resistance of the model, combination therapy induced potent T cell activation. Compared with αPD-1, SMG1i+αPD-1 increased activation of CD8^+^ and non-Treg (Foxp3^−^) CD4^+^ T cells, as indicated by upregulation of 4-1BB (CD137) on CD8^+^ T cells and OX40 (CD134) on CD4^+^ T cells, as well as PD-1 and inducible T-cell costimulator (ICOS, CD278) on both subsets (Figure 3C). In line with the *ex vivo* findings, CD8^+^PD-1^+^ T cells showed the highest levels of activation following SMG1i, as evidenced by the co-expression of several markers of activation (CD25), costimulation (ICOS and glucocorticoid-induced TNFR-related protein [GITR, CD357]), proliferation (Ki-67), and cytotoxicity (granzyme B [GzmB]) (Figures 3C and S4G), consistent with recent antigen encounter. Bulk TCR-seq showed a trend toward reduced overall clonotype diversity and increased expansion of large and hyperexpanded clones following SMG1i, alone or in combination with αPD-1, with a corresponding reduction in small and medium clones, further suggesting that the elevated T cell response is antigen dependent (Figures 3D, 3E, and S4H–S4J).

Together, these data show that NMD inhibition, particularly in combination with PD-1 blockade, promotes activation and clonal expansion of tumor-reactive tissue-resident T cells and improves tumor control in a CPI-refractory syngeneic model.

### NMDi stabilizes PTC-containing isoforms predicted to encode non-canonical neoantigens

**Figure 4:**
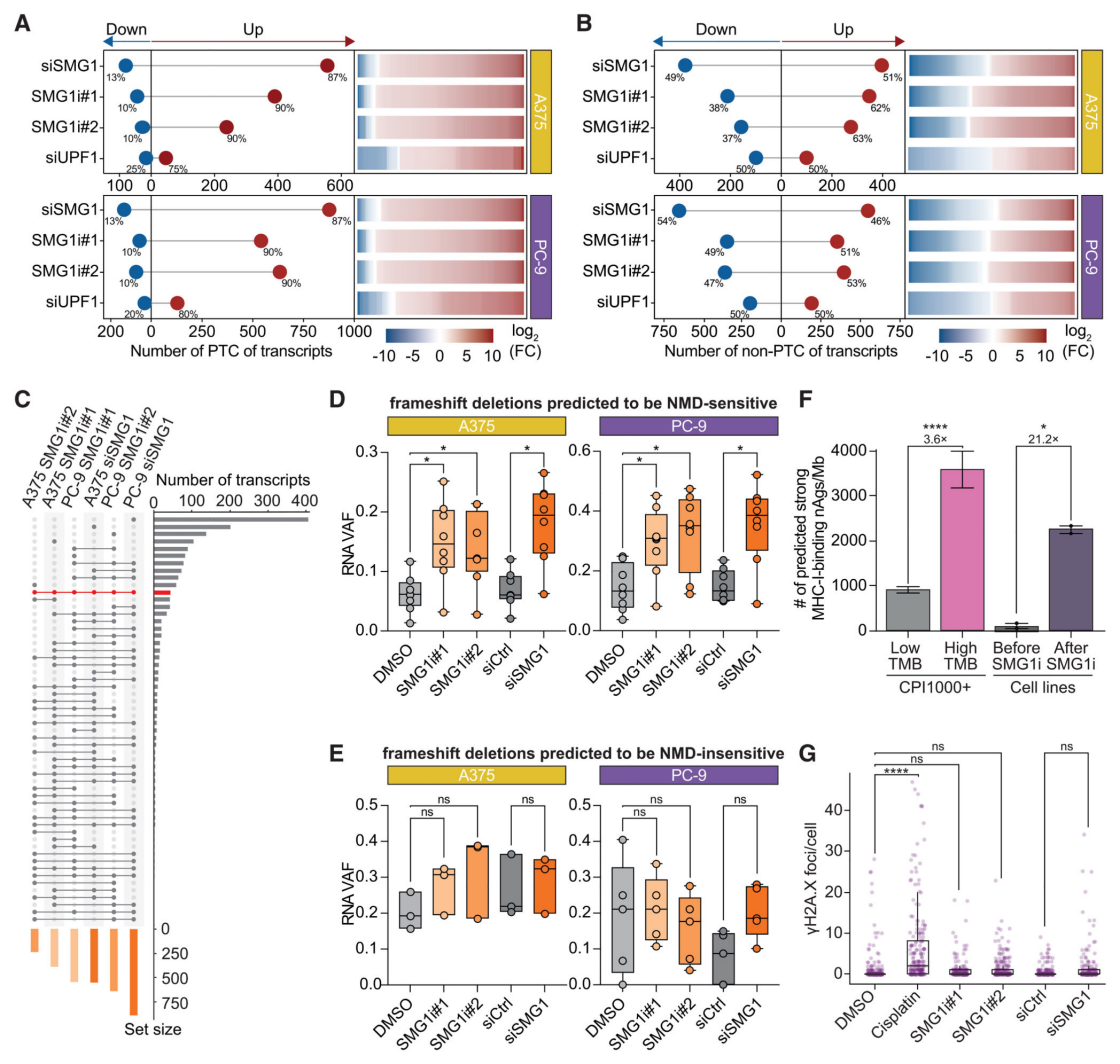
SMG1 inhibition promotes expression of PTC-containing isoforms encoding strong-binding neoantigens (A and B) (Left) Lollipop plots displaying the number of significantly (*q* < 0.05, rIF > 1.5) upregulated and downregulated PTC-containing transcripts (A) or non-PTC-containing transcripts (B), and (right) corresponding heatmaps showing log_2_FC, following SMG1 inhibition (SMG1i#1 or SMG1i#2), SMG1 knockdown (siSMG1), or UPF1 knockdown (siUPF1) relative to their respective controls (DMSO or siRNA control [siCtrl]) in A375 (top) and PC-9 (bottom) cells (*n* = 3 biological replicates). (C) UpSet plot of the overlap in significantly upregulated PTC-containing transcripts (*q* < 0.05, rIF > 1.5) following treatment with SMG1i#1, SMG1i#2, or siSMG1 in A375 and PC-9 cells. (D and E) RNA variant allele frequency (VAF) of transcripts arising from endogenous frameshift deletions that are (D) NMD-sensitive (subject to NMD-mediated degradation) or (E) NMD-insensitive (escaping NMD degradation) in A375 (left) and PC-9 (right) cells following treatment with DMSO, SMG1i#1, SMG1i#2, siCtrl, or siSMG1 (two-sided Wilcoxon matched-pairs signed-rank tests, with Holm-Šidák correction; *n* = 3 biological replicates). (F) Median (± 95% confidence interval) number of predicted strong binding neoantigens (NetMHCpan score < 0.5) per megabase (Mb) in CPI1000+ patients with TMB-low or -high cancers and cell lines (A375 and PC-9) before (DMSO and siCtrl) or after (SMG1i#1, SMG1i#2, and siSMG1) SMG1 targeting (Mann-Whitney U test, unpaired, for CPI1000+ samples; two-tailed Student’s *t* test, paired, for cell lines; dots denote individual cell lines, mean of *n* = 3 biological replicates). (G) Quantification of histone H2A.X phosphorylation at serine 139 (γH2A.X) foci per cell in A375 cells 72 h after treatment with DMSO, cisplatin (positive control, DNA damage inducer), SMG1i#1, SMG1i#2, siCtrl, or siSMG1 (two-sided Kruskal-Wallis test with Dunn’s post hoc test and Bonferroni correction; ≥200 individual cells quantified per condition across *n* = 3 biological replicates). Bar plots represent the mean ± SEM; for boxplots, boxes denote the interquartile range, the black line represents the median, and whiskers extend to 1.5× the interquartile range; ns, *p* > 0.05, *p ≤ 0.05, ****p ≤ 0.0001. See also Figure S5.

We next sought to define the molecular basis of SMG1i-mediated T cell activation. To do so, we first characterized transcriptomic changes in A375 and PC-9 cells following SMG1 knockdown or inhibition by performing RNA-seq followed by differential gene- and isoform-level expression analysis to accurately detect and quantify PTC-containing transcripts upon NMDi (Figure S5A). Both drugs and siRNAs drove broad yet similar changes, as confirmed by principal-component analysis (PCA; Figure S5B). The expression of PTC-containing transcripts was markedly and consistently upregulated across all treatment conditions and cell lines, recapitulating the effects observed *ex vivo* and showing substantial overlap with UPF1 knockdown (Figures S5C–S5F). Among PTC-containing transcripts with significant expression changes, nearly 90% were upregulated upon SMG1 knockdown or inhibition relative to controls, whereas this fraction was lower upon UPF1 knockdown (~75%–80% upregulated) (Figure 4A). Non-PTC-containing transcripts were instead evenly distributed between up- and downregulated genes (Figure 4B). Notably, a subset of significantly upregulated PTC-containing transcripts (3%, *n* = 44) was consistently detected across all cell lines upon SMG1 knockdown or inhibition (Figure 4C). We next evaluated the effect of SMG1 downregulation on transcripts originating from endogenous fs-indel mutations. DNA-level fs-indels in A375 and PC-9 cells were classified as NMD-sensitive or -insensitive based on predicted susceptibility to NMD-mediated degradation (see STAR Methods),^27^ and the RNA variant allele frequency (VAF) for each fs-indel was quantified. SMG1i selectively stabilized NMD-sensitive fs-indel transcripts, with no impact on NMD-insensitive ones (Figures 4D and 4E). Intriguingly, RNA-seq and proteomic analysis revealed upregulation of other NMD core components following SMG1 inhibition, suggesting a possible compensatory feedback loop (Figures S1D, S1F, and S5G). Despite this, gene set enrichment analysis (GSEA) of both exon-junction complex (EJC)-enhanced and EJC-independent NMD branches^99^ showed a marked decrease in overall NMD activity under these conditions, confirming SMG1 as a central regulator of the NMD pathway (Figure S5H).

Transcript biotype analysis revealed that these PTC-containing transcripts predominantly originated from putative NMD targets, retained introns, processed transcripts, and lncRNAs (Figure S5A). Many of these species are typically invisible to the antigen-processing machinery owing to NMD surveillance, yet their translation can generate non-mutational neoantigens with strong immunogenic potential.^17,47–57^ The widespread upregulation of these RNA species upon SMG1 inhibition suggested a broad remodeling of the non-canonical antigenic landscape as a result of the generation of splicing-derived peptides, pseudogene-encoded epitopes, and neoORF-driven antigens. To test this possibility, we estimated the impact of SMG1 downregulation on neoepitope production by *in silico* translation of all 8–14-mer peptides arising from the PTC-containing transcripts identified in our RNA-seq data and predicting their MHC class I binding affinity with NetMHCpan.^100^ Compared with controls, SMG1 knockdown induced an approximately 20-fold increase in predicted MHC class I strong-binding neoantigens (Figure 4F), reaching levels comparable to those observed in TMB-high cancers (≥10 mutations/Mb) in the CPI1000+ cohort. Crucially, this expansion occurred without evidence of DNA damage (Figures 4G and S5I), demonstrating that NMD modulation has the potential to increase neoantigen load without inducing genotoxic damage, unlike chemotherapy or radiotherapy.

Taken together, these findings indicate that SMG1 downregulation prevents the degradation of a pool of NMD-targeted transcripts, substantially expanding the predicted neoepitope repertoire to a level comparable to TMB-high cancers.

### NMDi-derived neoantigens are presented on tumor cells via MHC class I and elicit T cell reactivity

**Figure 5:**
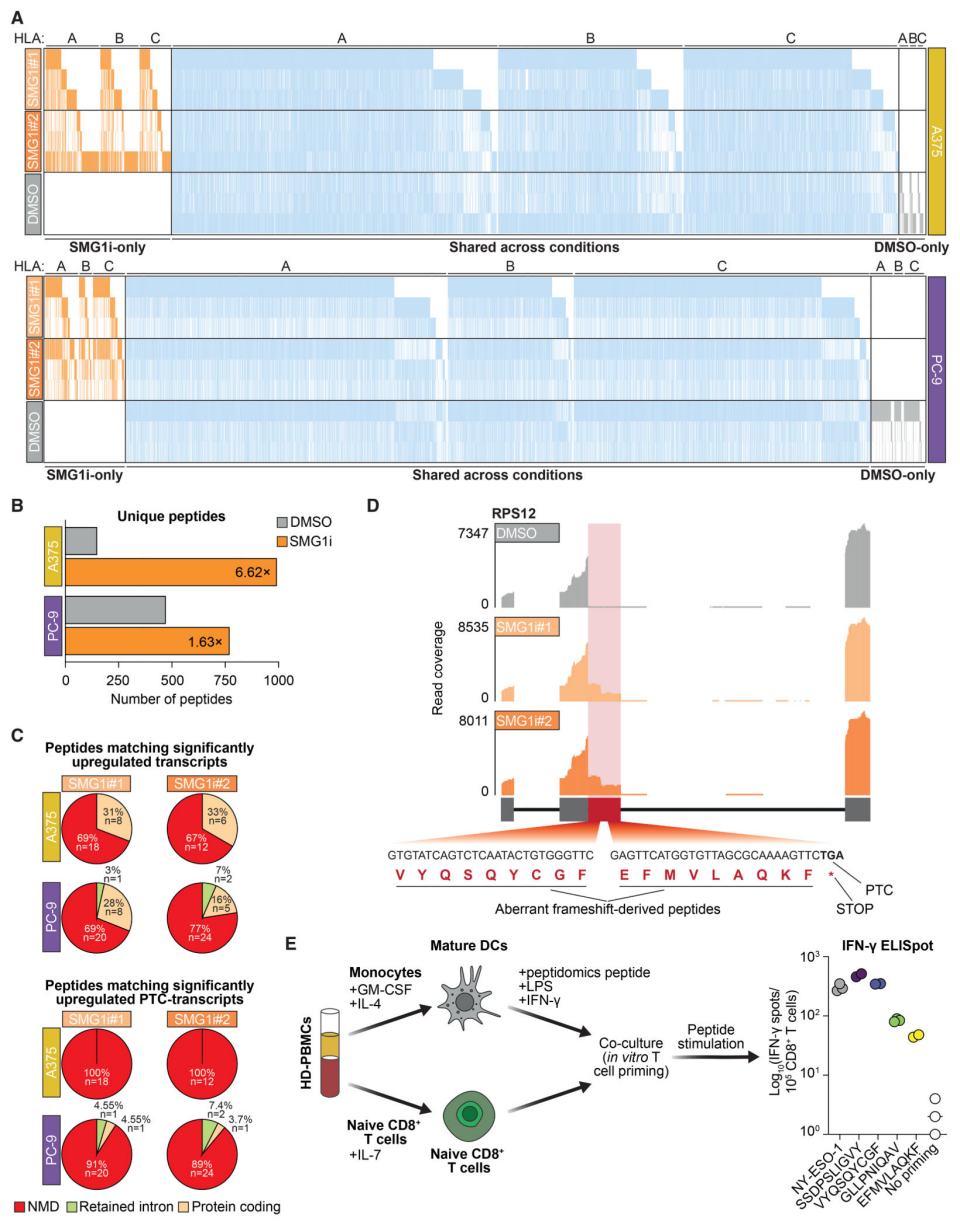
SMG1 inhibition promotes presentation of immunogenic neoantigens derived from PTC-containing transcripts via MHC class I (A) Heatmap displaying peptides (columns) detected in at least one replicate (each row corresponds to a biological replicate) in A375 and PC-9 cells following treatment with DMSO, SMG1i#1, or SMG1i#2 (*n* = 3 biological replicates). (B) Bar charts displaying the number of unique peptides identified in at least one replicate in A375 and PC-9 cells following treatment with DMSO, SMG1i#1, or SMG1i#2. (C) Pie charts showing the proportion of PTC-derived peptides originating from all significantly upregulated transcripts (top) or only significantly upregulated PTCcontaining transcripts (bottom) identified in A375 (top) and PC-9 (bottom) cells. (D) Representative RNA-seq coverage plots (top) and schematic illustration (bottom) of two SMG1i-induced frameshift-derived neoantigens originating from a PTC-containing transcript generated by intron retention in RPS12. (E) Schematic of the *in vitro* T cell priming assay (left), in which peptide-pulsed mature dendritic cells (DCs) from healthy donor peripheral blood mononuclear cells (HD PBMCs) are used to test the immunogenic potential of SMG1i-induced peptides by priming donor-matched naive CD8^+^ T cells, followed by assessment of their activation upon peptide re-exposure. CD8^+^ T cell IFN-γ secretion was quantified by ELISpot (right) and shown as log10-transformed spot counts per 105 CD8^+^ T cells. NY-ESO-1 SLLMWITQC (NYE157–165) was used as an immunogenic antigen control (*n* = 2–3 technical replicates). See also Figure S6 and Table 1.

We next asked whether this expanded pool of predicted NMD-derived neoantigens is processed and presented by tumor cells. To this end, we complemented our transcriptomic analyses with MHC class I immunopeptidomics in A375 and PC-9 cells. MHC class I-bound peptides were isolated and analyzed by liquid chromatography-tandem mass spectrometry (LC-MS/MS). Because peptide detection is highly dependent on the reference proteome,^101^ conventional database searches often fail to capture peptides arising from aberrant/cryptic sequences. To overcome this limitation, we generated an expanded reference database by incorporating the *in silico*-translated sequences from PTC-containing transcripts identified in our RNA-seq analyses (Figures 4 and S5). This customized search space enabled the detection of NMDi-derived peptides that would otherwise be excluded by standard proteomic workflows. Immunopeptidomics identified 4,990 and 5,872 predicted MHC class I binders (NetMHCpan rank < 2) in A375 and PC-9 cells, respectively (Figures 5A and S6A). SMG1i treatment increased the number of uniquely recovered peptides by ~6.6-fold in A375 and ~1.6-fold in PC-9 compared with controls (Figures 5B and S6B). Filtering for peptides corresponding to transcripts that were significantly upregulated in the matched RNA-seq data revealed that most (>70%) induced peptides originated from transcripts annotated as putative NMD targets and retained introns (Figure 5C). Further comparison with immunopeptidomes from normal tissues identified eight aberrant peptides uniquely induced by SMG1i, all validated by spectral matching to synthetic standards (Figures S6C and S6D; Table 1). Six out of eight (75%) were predicted as strong MHC class I binders (rank < 0.5) and matched transcript-level induction upon SMG1i (Figure 5D; Table 1), supporting enhanced translation and presentation of stabilized PTC-containing isoforms upon NMDi.

Many peptides identified were out of frame and consequently highly distinct from self-peptides, a feature associated with enhanced immunogenicity.^26,50,51,102^ To evaluate whether the aberrant peptides identified upon SMG1i are immunogenic, we performed *in vitro* priming using naive CD8^+^ T cells from from human leukocyte antigen (HLA)-matched healthy donors (available for six of the eight peptides) (Figure 5E).^103,104^ Monocyte-derived dendritic cells (MoDCs) were pulsed with the candidate peptides and co-cultured with autologous naive CD8^+^ T cells. After two rounds of priming, CD8^+^ T cells were restimulated with the same peptide, and immunogenicity was assessed by IFN-γ secretion using IFN-γ enzyme-linked immunosorbent spot (ELISpot). Notably, four out of six peptides (66.7%) tested elicited IFN-γ responses comparable to the known NY-ESO-1 epitope (SLLMWITQC, NY_157–165_),^105^ underscoring their immunogenicity *in vitro* (Figures 5E, S6E, and S6F).

Together, these findings demonstrate that NMDi promotes MHC class I presentation of neoantigens derived from aberrant transcripts normally degraded by NMD and that these neoantigens can elicit potent T cell activation.

### NMDi induces NMD- and antigen-dependent T cell activation and tumor cell killing

**Figure 6:**
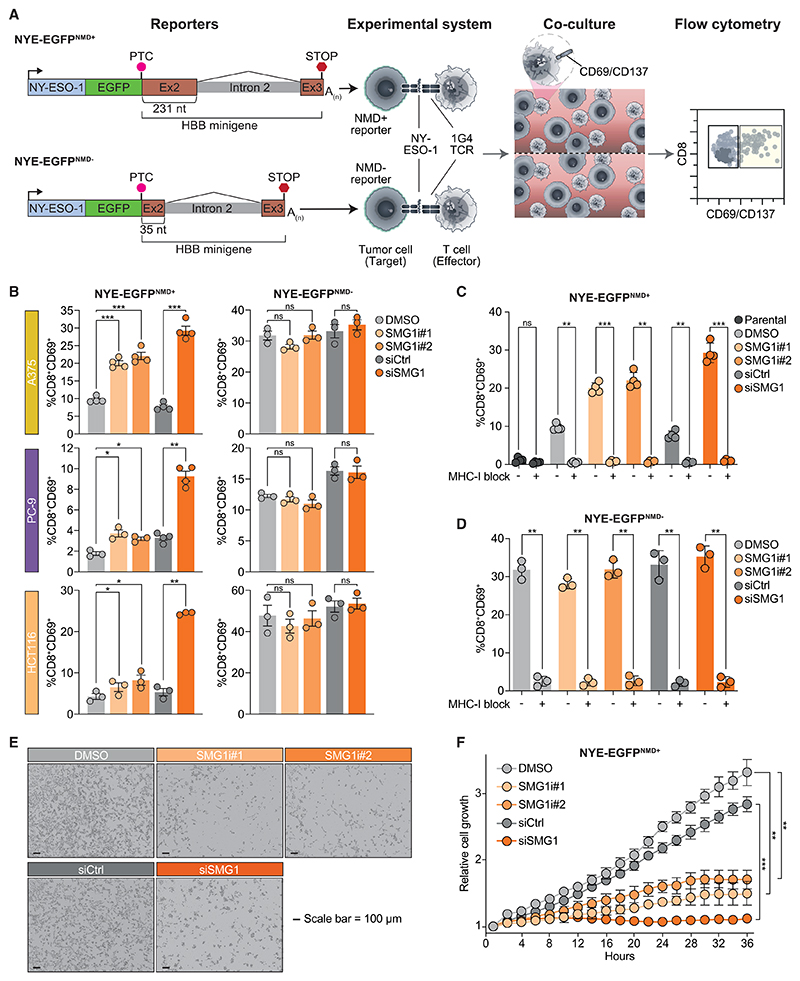
SMG1 inhibition promotes tumor immunogenicity and T cell killing in an NMD- and antigen-dependent manner (A) Schematic representation of the NYE-EGFP^NMD+^ and NYE-EGFP^NMD−^ reporters and the co-culture experimental system; nt, nucleotides. (B) CD69 surface expression (% positive) in 1G4 CD8^+^ Jurkat T cells co-cultured for 24 h with tumor cells (A375 [top], PC-9 [middle], and HCT116 [bottom]) expressing either the NYE-EGFP^NMD+^ (left) or NYE-EGFP^NMD−^ (right) reporter pre-treated for 48 h with DMSO, SMG1i#1, SMG1i#2, siCtrl, or siSMG1 (one-way repeated-measures ANOVA with Holm-Šidák correction, using a mixed-effect model to account for missing data; *n* = 3–4 biological replicates). (C and D) CD69 surface expression (% positive) in 1G4 CD8^+^ Jurkat T cells co-cultured for 24 h with A375 cells expressing the NYE-EGFP^NMD+^ (C) or NYE-EGFP^NMD−^ (D) reporter pre-treated for 48 h with DMSO, SMG1i#1, SMG1i#2, siCtrl, or siSMG1, in the presence (+) or absence (−) of an MHC class I blocking antibody (one-way repeated-measures ANOVA with Holm-Šidák correction; *n* = 3–4 biological replicates). Parental: A375 cells not expressing the NMD+ reporter. (E) Representative images of A375 cells expressing the NYE-EGFP^NMD+^ reporter treated for 48 h with DMSO, SMG1i#1, SMG1i#2, siCtrl, or siSMG1, and subsequently co-cultured with 1G4-expressing healthy donor CD8^+^ T cells for 36 h. Scale bar, 100 μm. (F) Quantification of tumor cell killing in A375-T cell co-cultures as described in (E). Statistical significance (two-way repeated measures ANOVA with Tukey’s multiple comparisons test; *n* = 4 biological replicates) shown corresponds to the endpoint (36 h). Bar plots represent the mean ± SEM; ns, *p* > 0.05, *p ≤ 0.05, **p ≤ 0.01, ***p ≤ 0.001. See also Figure S7.

To dissect the mechanism by which SMG1 downregulation enhances tumor immunogenicity, we modified the NMD reporter employed in Figure S1N by fusing the NY-ESO-1 epitope (SLLMWITQC, NY_157–165_)^105^ to its N terminus (termed NYE-EGFP^NMD+^) (Figure 6A). A matched NMD-insensitive construct (termed NYE-EGFP^NMD−^) was generated in parallel (Figure 6A, see STAR Methods). This paired reporter system enabled a controlled assessment of T cell activation upon NMDi by comparing epitopes generated from transcripts that are either NMD-targeted or that escape NMD, thereby isolating the specific contribution of NMD suppression to antigen-driven immune recognition. We next generated stable cell lines (A375, melanoma; PC-9, lung cancer; and HCT116, CRC) and LUAD patient-derived tumor organoids (PDTOs) from the TRACERx study^106,107^ expressing these reporters. SMG1 downregulation led to a marked fluorescence increase only in cells harboring the NYE-EGFP^NMD+^ reporter, confirming reporter specificity and reduced NMD pathway activity (Figure S7A).

Next, tumor cells and PDTOs expressing the NYE-EGFP^NMD+^ or NYE-EGFP^NMD−^ reporters were treated with SMG1 siRNAs or inhibitors. These cells were then co-cultured with CD8^+^ T cells engineered to express the 1G4 TCR, which selectively recognizes the NY-ESO-1_157–165_ epitope encoded by both reporters (Figure 6A).^105,108,109^ SMG1 downregulation enhanced T cell activation exclusively in cells expressing the NYE-EGFP^NMD+^ reporter, with no increase in NYE-EGFP^NMD−^ controls, reporter-negative cells, or T cells alone (Figures 6B and S7B–S7D). This showed that NMD downregulation can promote the generation of antigens otherwise subjected to NMD degradation without altering the immunogenicity of those that naturally escape NMD. Additionally, this effect was strictly MHC class I-dependent, as blockade of MHC class I, but not MHC class II, abolished T cell activation (Figures 6C, 6D, S7B, S7C, and S7E). Live-cell imaging confirmed potent T cell-mediated killing of A375 cells expressing the NYE-EGFP^NMD+^ reporter following SMG1 downregulation (Figures 6E and 6F; Video S1). Importantly, the expression of MHC class I genes (HLA-A, HLA-B, HLA-C, and beta-2-microglobulin [B2M]) was not altered following SMG1 downregulation, indicating that enhanced T cell activation was driven by increased antigen availability rather than higher abundance of MHC class I molecules (Figure S7F).

Together, these results demonstrate that the stabilization of NMD-targeted transcripts by NMD inhibition can increase tumor immunogenicity by enabling MHC class I-dependent presentation of peptides originating from transcripts normally degraded by the NMD pathway, thereby eliciting antigen-specific activation and killing by cytotoxic T cells.

## Discussion

The antigenic landscape of tumors extends far beyond canonical mutational and viral sources. Dysregulated transcription, splicing, and translation generate a rich repertoire of non-mutational neo-antigens, with the potential to expand the scope of cancer immunotherapy.^17,47–57^ Here, we identify NMD-targeted transcripts as a previously unrecognized and mechanistically distinct source of tumor neoantigens contributing to this non-mutational neoantigen pool. Inhibition of the core NMD mediator SMG1 unmasks a novel class of tumor neoantigens by stabilizing PTC-containing transcripts arising from both mutational and non-mutational (including retained introns, pseudogenes, and lncRNAs) sources. This fundamentally expands the antigenic repertoire beyond DNA-derived neoantigens and elevates the predicted neoepitope load to levels comparable to TMB-high cancers. Crucially, these antigens are functionally relevant and elicit robust T cell activation and MHC class I-dependent tumor cell killing. Moreover, NMD inhibition increases both the expression of PTC-derived neoantigens and the fraction of tumor cells that express them, an important clinical consideration given that both total antigen abundance and the proportion of antigen-expressing cells are key determinants of immune recognition.^110,111^

We report, to our knowledge, the first demonstration that pharmacologic SMG1 inhibition enhances T cell activation *ex vivo*. Intriguingly, the strongest effects arose within the effector memory compartment, indicative of recall responses to previously encountered antigens and consistent with the notion that NMD in tumors is inherently “leaky.”^44–47^ Such leakage may allow presentation of PTC-derived peptides in the tumor-draining lymph nodes, enabling priming and the subsequent establishment of tissue-resident populations within the tumor. As tumors evolve and immune pressure increases, elevated NMD activity may progressively restrict the availability of these antigens, maintaining presentation below the threshold required for sustained T cell activation, thus promoting immune escape. In this context, it is tempting to speculate that a fraction of the clonally expanded, yet dysfunctional, TILs observed in human tumors may be specific for these NMD-derived neoantigens whose presentation in late-stage tumors is constrained below this activation threshold. In this setting, acute SMG1 inhibition could restore antigen abundance above this activation threshold, thereby promoting anti-tumor immunity. The DDX20-derived peptide identified in this study exemplifies this model, as it is detectable at low levels under control conditions but becomes robustly presented following NMD inhibition and elicits a potent immune response *in vitro*. Moreover, although the LLC1 mouse syngeneic model used in our *in vivo* studies is characterized by a myeloid-rich TME,^98^ which may be more responsive to therapies targeting myeloid-driven immunosuppression,^112^ the increased T cell activation and tumor growth delay achieved with combined NMD inhibition and PD-1 blockade indicates that such an approach remains effective even in this setting, and can augment CPI responses in otherwise resistant models.

The broad immunoregulatory role of NMD is underscored by genetic studies linking its defective function to both systemic inflammation and cancer susceptibility. In mice, *Smg1* haploin-sufficiency promotes chronic inflammatory phenotypes and increased tumor incidence, while in humans, congenital NMD defects have been associated with aberrant immune activation and autoimmune pathology in non-malignant contexts, including neurodevelopmental and muscular dystrophy-associated inflammatory disorders,^113–115^ suggesting that sustained impairment of NMD can drive persistent immune activation. Prolonged NMD suppression in tumors could similarly lead to continuous neoantigen exposure, chronic inflammation, and progressive T cell exhaustion, ultimately limiting durable anti-tumor immunity. By contrast, our approach uses acute and transient NMD inhibition to increase tumor antigen load, providing evidence that this strategy may enhance tumor immunogenicity, particularly in combination with CPIs, while limiting long-term inflammatory toxicity. Consistent with this, our *in vivo* studies show that short-term pharmacologic NMD inhibition reduces tumor growth without overt systemic toxicity. Furthermore, in contrast to earlier strategies that broadly inhibited the NMD pathway,^116–119^ selective SMG1 targeting provides greater specificity and reduces the likelihood of undesired off-target effects. Moreover, by increasing the abundance of aberrant peptide-encoding transcripts without inducing widespread DNA damage, this approach also reduces the risk of therapy-induced secondary malignancies.^120–123^

Together, our findings demonstrate that targeting the core NMD component SMG1 reshapes the immunogenic landscape of tumors by promoting the generation of a previously underexplored, yet immunologically potent, source of both canonical and non-canonical neoantigens. These results indicate that NMD inhibition broadens the spectrum of targetable antigens in cancer and enhances anti-tumor immunity, even in low-TMB and CPI-refractory settings. In light of recent advances in cancer vaccines and cell-based therapies,^124–131^ NMD-derived antigens represent a promising pool of targets for the development of both public and personalized vaccines and T cell-based approaches.

## Limitations of the study

This study provides insight into the early immune consequences of NMD suppression *ex vivo*. However, the breadth of our conclusions is constrained by the experimental systems available. First, PDFs remain viable for ~120 h, enabling assessment of early responses from previously primed, tissue-resident T cells but precluding evaluation of later-stage events such as T cell priming. In addition, limited tissue availability prevented immunopeptidomic profiling of PDFs following SMG1i, thereby precluding direct identification of the antigens driving T cell activation. As a result, we cannot exclude the possibility that the observed *ex vivo* T cell responses were driven, at least in part, by non-NMD-derived neoantigens. Integration of emerging platforms such as organotypic tumor slices and tumor-on-a-chip systems may help overcome these limitations and enable direct antigen and TCR identification.^132^

Furthermore, although we observed CD4^+^ T cell activation in both *ex vivo* and *in vivo* settings, limitations in MHC class II binding prediction and peptidomics precluded assessment of MHC class II antigen contributions.^47,133^ Future studies integrating MHC class II peptidomics could expand the repertoire of targetable neoantigens and broaden therapeutic reach.

Finally, while both pharmacologic inhibition and siRNA-mediated knockdown of SMG1 increased PTC-containing transcripts, the efficacy and overlap of the SMG1 inhibitors were comparatively lower. This may reflect differences in binding affinity, conformational dynamics, site engagement, and kinetics, factors commonly observed with small-molecule inhibitors.^134–136^ Notably, the SMG1 inhibitor used *in vivo* (KVS0001, a derivative of SMG1i#1, the only *in vivo*-compatible SMG1 inhibitor currently available)^93^ also exhibited reduced potency *in vitro* compared with the parent compound, raising the possibility that full SMG1 inhibition may not have been achieved *in vivo*. Our findings will hopefully motivate further efforts to develop SMG1 inhibitors with greater potency and clinical applicability.

## Resource Availability

### Lead contact

Requests for further information, resources, and reagents should be directed to and will be fulfilled by the lead contact, Kevin Litchfield (k.litchfield@ucl. ac.uk).

### Materials availability

All materials generated in this study are available from the lead contact with a completed materials transfer agreement (MTA).

## Star★Methods

### Key Resources Table

**Table:** 

REAGENT or RESOURCE	SOURCE	IDENTIFIER
Antibodies		
Rabbit anti-human SMG-1 (clone D42D5)	Cell Signaling Technology	Cat#9149; RRID: AB_10860072
Rabbit anti-human phospho-Upf1 (Ser1127)	Sigma-Aldrich	Cat#07-1016; RRID: AB_10805931
Rabbit anti-human UPF1 (RENT1)	Bethyl Lab	Cat#A300-038A; RRID: AB_2288326
Rabbit anti-human GAPDH	Abcam	Cat#ab9485; RRID: AB_307275
Mouse anti-human alpha-Tubulin (clone B-5-1-2)	Sigma-Aldrich	Cat#T6074; RRID: AB_477582
Sheep anti-mouse IgG-HRP	Cytiva	Cat#NA931; RRID: AB_772210
Donkey anti-rabbit IgG-HRP	Cytiva	Cat#NA934; RRID: AB_772206
Mouse anti-goat IgG-HRP	Santa Cruz	Cat#sc-2354; RRID: AB_628490
Goat anti-rabbit IgG-HRP	Cell Signaling Technology	Cat#7074; RRID: AB_2099233
Purified hamster InVivoMAb anti-mouse PD-1 (CD279; clone RMP1-14)	BioXcell	Cat#BE0146; RRID: AB_10949053
Mouse anti-human HLA-ABC (clone W6/32)	Biolegend	Cat#311427; RRID: AB_2561492
Mouse anti-human HLA-DR, DP, DQ (clone Tu39)	BD Biosciences	Cat#555556; RRID: AB_39593
Mouse anti-human CD3 (clone OKT3)	ThermoFisher Scientific	Cat#16-0037-81; RRID: AB_468854
Mouse anti-human CD28 (clone CD28.2)	ThermoFisher Scientific	Cat#16-0289-85; RRID: AB_468927
Rabbit anti-human phospho-(Ser/Thr) ATM/ATR Substrate (clone 4F7)	Cell Signaling Technology	Cat#2909; RRID: AB_2163443
Rabbit anti-human phospho-histone H2A.X (Ser139, clone 20E3)	Cell Signaling Technology	Cat#9718; RRID: AB_2118009
Goat anti-rabbit IgG (H+L) (Alexa Fluor™ 488 conjugated)	ThermoFisher Scientific	Cat#A-11008; RRID: AB_143165
Mouse anti-human pan-HLA W6/32	ATCC	Cat#HB-95, RRID: N/A
Rat anti-mouse CD16/32	BioLegend	Cat#101302; RRID: AB_312801
Mouse anti-human CD3 (clone SK7; BB700 conjugated)	BD Biosciences	Cat# 566575; RRID: AB_2860004
Mouse anti-human CD3 (clone SK7; BUV805 conjugated)	BD Biosciences	Cat#612893; RRID: AB_2870181
Mouse anti-human CD4 (clone RPA-T4; FITC conjugated)	BD Biosciences	Cat#555346; RRID: AB_395751
Mouse anti-human CD4 (clone SK3; BB790-P custom conjugated)	BD Biosciences	N/A
Mouse anti-human CD8 (clone RPA-T8; BV421 conjugated)	BD Biosciences	Cat# 562428; RRID: AB_11154035
Mouse anti-human CD8 (clone RPA-T8; BUV496 conjugated)	BD Biosciences	Cat#612942; RRID: AB_2870223
Mouse anti-human CD45 (clone HI30; BV785 conjugated)	BioLegend	Cat#304048; RRID: AB_2563129
Mouse anti-human CD45RA (clone HI100; BUV563 conjugated)	BD Biosciences	Cat#612926; RRID: AB_2870211
Mouse anti-human CD69 (clone FN50; BV650 conjugated)	BD Biosciences	Cat#563835; RRID: AB_2738442
Mouse anti-human CD137 (4-1BB; clone 4B4-1; PE conjugated)	BD Biosciences	Cat#550890; RRID: AB_398477
Mouse anti-human CD137 (4-1BB; clone 4B4-1; PE/ Dazzle 594 conjugated)	BioLegend	Cat#309826; RRID: AB_2566260
Mouse anti-human CD197 (CCR7; clone G043H7; BV650 conjugated)	BioLegend	Cat#353234; RRID: AB_2563867
Mouse anti-human CD279 (PD-1; clone EH12.2H7; BV421 conjugated)	BioLegend	Cat#329920; RRID: AB_10960742
Rat anti-human Foxp3 (clone PCH101; APC conjugated)	ThermoFisher Scientific	Cat#17-4776-42; RRID: AB_1603280
Mouse anti-human TCRαβ(clone IP26; PE conjugated)	BD Biosciences	Cat#564728; RRID: AB_2738921
Rat anti-mouse CD3 (clone 17A2; BUV737 conjugated)	BD Biosciences	Cat#612803, RRID: AB_2738781
Rat anti-mouse CD4 (clone GK1.5; BUV496 conjugated)	BD Biosciences	Cat#612952, RRID: AB_2722549
Rat anti-mouse CD8a (clone 53-6.7; BUV805 conjugated)	BD Biosciences	Cat#564920, RRID: AB_2870186
Rat anti-mouse CD11b (clone M1/70; BUV661 conjugated)	BD Biosciences	Cat#612977, RRID: AB_2870249
Rat anti-mouse CD25 (clone PC61; FITC conjugated)	BioLegend	Cat#102006, RRID: AB_312854
Mouse anti-human CD34 (RQR8; clone QBEnd10; Alexa Fluor 488 conjugated)	R&D Systems	Cat#FAB7227G, RRID: AB_10973657
Rat anti-mouse CD45 (clone 30-F11; BUV563 conjugated)	BD Biosciences	Cat#612924, RRID: AB_2722550
Rat anti-mouse CD134 (OX40; clone OX86; BV786 conjugated)	BD Biosciences	Cat#740945, RRID: AB_2740573
Hamster anti-mouse CD137 (4-1BB; clone 17B5; BV711 conjugated)	BD Biosciences	Cat#756686, RRID: N/A
Hamster anti-human/mouse/rat CD278 (ICOS; clone C398.4A; PE/Cy7 conjugated)	BioLegend	Cat#313520, RRID: AB_10641839
Rat anti-mouse CD279 (PD-1; clone 29F.1A12; PE/Dazzle 594 conjugated)	BioLegend	Cat#135228, RRID: AB_2566006
Rat anti-mouse CD357 (GITR; clone DTA-1; BV510 conjugated)	BD Biosciences	Cat#740192, RRID: AB_2739945
Rat anti-mouse Foxp3 (clone FJK-16s; eFluor450 conjugated)	ThermoFisher Scientific	Cat#48-5773-82, RRID: AB_1518812
Mouse anti-human/mouse granzyme B (clone GB11; APC conjugated)	ThermoFisher Scientific	Cat#GRB05, RRID: AB_2536539
Rat anti-human/mouse/rat Ki-67 (clone SolA15; AF700 Conjugated)	ThermoFisher Scientific	Cat#56-5698-80, RRID: AB_2637479
Mouse anti-mouse NK-1.1 (clone PK136; BUV395 conjugated)	BD Biosciences	Cat#564144, RRID: AB_2738618
Bacterial and virus strains		
DH5α Competent Cells	ThermoFisher Scientific	Cat#EC0111
One Shot™ Stbl3™ Chemically Competent E. coli	ThermoFisher Scientific	Cat#C737303
Biological samples		
Healthy donors PBMCs	Tebu-Bio	297CTIPB.1.24-fresh
Non-small cell lung cancer resection specimens (for organoid derivation)	TRACERx study^106^,^107^	CRUK1398
Colorectal cancer specimens (for PDTF derivation)	UCL/UCLH Biobank	REC#20/YH/0088
Normal-adjacent specimens (for PDNF derivation)	UCL/UCLH Biobank	REC#20/YH/0088
Chemicals, peptides, and recombinant proteins		
SBE-β-CD	MedChemExpress	Cat#HY-17031
DMEM, high glucose, GlutaMAX supplement, pyruvate	ThermoFisher Scientific	Cat#31966021
RPMI 1640 Medium, GlutaMAX supplement	ThermoFisher Scientific	Cat#61870010
X-VIVO 15 serum-free hematopoietic cell medium	Lonza	Cat#02-053Q
DMEM-F12	ThermoFisher Scientific	Cat#11320033
Advanced DMEM-F12	GIBCO	Cat#12634010
Opti-MEM reduced serum medium	ThermoFisher Scientific	Cat#31985070
Fetal bovine serum, qualified, heat inactivated, Brazil	ThermoFisher Scientific	Cat#10500064
Human serum, from human male AB plasma	Sigma-Aldrich	Cat#H3667
Bovine serum albumin	Sigma-Aldrich	Cat#A9418
Mouse serum	Sigma-Aldrich	Cat#M5905
Rat serum	Sigma-Aldrich	Cat#R9759
Rabbit serum	Sigma-Aldrich	Cat#R9133
Normal goat serum	ThermoFisher Scientific	Cat#10000C
DPBS, no calcium, no magnesium	ThermoFisher Scientific	Cat#14190136
L-Glutamine	ThermoFisher Scientific	Cat#25030081
Ultraglutamine type I	Lonza	Cat#BE17-605E
B27 supplement	ThermoFisher Scientific	Cat#17504044
N-2 Supplement	ThermoFisher Scientific	Cat#17502048
Recovery cell culture freezing medium	ThermoFisher Scientific	Cat#12648010
Lipofectamine™2000 transfection reagent	ThermoFisher Scientific	Cat#11668027
Lipofectamine™ RNAiMAX transfection reagent	ThermoFisher Scientific	Cat#13778150
GeneJuice® transfection reagent	Sigma-Aldrich	Cat#70967-5
RetroNectin® recombinant human fibronectin fragment	Takara Bio	Cat#T100
Penicillin-streptomycin	ThermoFisher Scientific	Cat#15140122
Trypsin-EDTA (0.05%), phenol red	ThermoFisher Scientific	Cat#25300054
Sodium pyruvate	ThermoFisher Scientific	Cat#11360070
MEM non-essential amino acids solution	ThermoFisher Scientific	Cat#11140035
Primocin	InvivoGen	Cat#ant-pm-1
Hygromycin B	ThermoFisher Scientific	Cat#J67371.XF
Puromycin	InvivoGen	Cat#ant-pr-1
Ampicillin sodium salt	Sigma-Aldrich	Cat#A9518
Kanamycin sulfate from Streptomyces kanamyceticus	Sigma-Aldrich	Cat#K1377
LB agar, Vegitone	Sigma-Aldrich	Cat#19344
LB broth (Luria low salt)	Sigma-Aldrich	Cat#L3397
Dimethyl sulfoxide	Sigma-Aldrich	Cat#276855
PageRuler™ Plus prestained protein ladder, 10 to 250 kDa	ThermoFisher Scientific	Cat#26620
HiMark™ pre-stained protein standard	ThermoFisher Scientific	Cat#LC5699
NuPAGE™ MES SDS running buffer (20X)	ThermoFisher Scientific	Cat#NP0002
NuPAGE™ MOPS SDS running buffer (20X)	ThermoFisher Scientific	Cat#NP0001
NuPAGE™ tris-acetate SDS running buffer (20X)	ThermoFisher Scientific	Cat#LA0041
iBlot™ 2 transfer stacks, nitrocellulose, mini	ThermoFisher Scientific	Cat#IB23002
iBlot™ 2 transfer stacks, nitrocellulose, regular	ThermoFisher Scientific	Cat#IB23001
Bolt™ bis-tris plus mini protein gels, 4%–12%	ThermoFisher Scientific	Cat#NW04120BOX
NuPAGE™ tris-acetate mini protein gels, 3% to 8%	ThermoFisher Scientific	Cat#EA0375BOX
Halt™ protease and phosphatase inhibitor cocktail	ThermoFisher Scientific	Cat#78442
cOmplete protease inhibitor cocktail	Roche	Cat#11873580001
PhosSTOP™ tablet	Roche	Cat#4906845001
Protease inhibitor cocktail	Sigma-Aldrich	Cat#P8340
Fast SYBR green master mix	ThermoFisher Scientific	Cat#4385612
Paraformaldehyde 16% (w/v) in aqueous solution methanol-free	ThermoFisher Scientific	Cat#043368.9M
DAPI	ThermoFisher Scientific	Cat#62248
Vectashield	Vector laboratories	Cat#H1200
Triton X-100	Sigma-Aldrich	Cat#X100
Tween 20	Sigma-Aldrich	Cat#P1379
Lysyl endopeptidase, mass spectrometry grade (Lys-C)	FUJIFILM Wako	Cat#129-02541
Tris(2-carboxyethyl)phosphine hydrochloride	Sigma-Aldrich	Cat#C4706
2-Chloroacetamide	Sigma-Aldrich	Cat#22790
Triethylammonium bicarbonate buffer	Sigma-Aldrich	Cat#T7408
Sequencing grade modified trypsin	Promega	Cat#V5113
Trifluoroacetic acid	Sigma-Aldrich	Cat#302031
Protein A resin	GenScript	Cat#L00210
Glo substrate reagent pack	R&D Systems	Cat#DY993
ON-TARGETplus™ non-targeting pool	Horizon Discovery	Cat#D-001810-10-20
ON-TARGETplus™ human UPF1 (5976) siRNA - SMARTpool™	Horizon Discovery	Cat#L-011763-00-0010
ON-TARGETplus™ human SMG1 (23049) siRNA - SMARTpool™	Horizon Discovery	Cat#L-005033-00-0010
SMG1i#1	Genesis Therapeutics	N/A
SMG1i#2	Genesis Therapeutics	N/A
KVS0001	MedChemExpress	Cat#HY-161111
Rapamycin	MedChemExpress	Cat#HY-10219
Indisulam	Sigma-Aldrich	Cat#SML1225
Synthetic peptides	GenScript Biotech Corp.	N/A
Proleukin® (aldesleukin) recombinant IL-2	Clingen	Cat#PL31644/0003
Human IL-4, animal-free recombinant protein	PeproTech	Cat#AF-200-04
Human IL-7, animal-free recombinant protein	PeproTech	Cat#AF-200-07
Human IL-15, animal-free recombinant protein	PeproTech	Cat#AF-200-15
Human IL-21, animal-free recombinant protein	PeproTech	Cat#AF-200-21
Human IFN-gamma, animal-free recombinant protein	PeproTech	Cat#AF-300-02
Human GM-CSF, animal-free recombinant protein	PeproTech	Cat#AF-300-03
Human EGF recombinant protein	PeproTech	Cat#AF-100-15
Human FGF-basic (FGF-2/bFGF) (154 aa) recombinant protein	PeproTech	Cat#100-18B
Lipopolysaccharides from Escherichia coli O111:B4	Sigma-Aldrich	Cat#L2630
Human CD14 microbeads	Miltenyi Biotec	Cat#130-050-201
Human CD45RO microbeads	Miltenyi Biotec	Cat#130-046-001
Human CD57 microbeads	Miltenyi Biotec	Cat#130-092-073
Y-27632	Selleckchem	Cat#S1049
Geltrex™ LDEV-free reduced growth factor basement membrane	ThermoFisher Scientific	Cat#A1413202
Collagenase type I	ThermoFisher Scientific	Cat#17100017
Collagenase type II	Sigma-Aldrich	Cat#C6885
DNase I	Sigma-Aldrich	Cat#4716728001
HEPES	ThermoFisher Scientific	Cat#15630080
Accutase	ThermoFisher Scientific	Cat#A1110501
Dispase type II	Sigma-Aldrich	Cat#D4693
Benzonase	Sigma-Aldrich	Cat#70746-3
Phorbol 12-myristate 13-acetate (PMA)	Sigma-Aldrich	Cat#19-144
Ionomycin	Sigma-Aldrich	Cat#I9657
Ficoll-Paque™ PLUS	GE Healthcare	Cat#17-1440-02
Polyethylenimine (PEI)	Polysciences	Cat#23966
Polybrene	Sigma-Aldrich	Cat#TR-1003-G
MluI	ThermoFisher Scientific	Cat#ER0561
NcoI	ThermoFisher Scientific	Cat#ER0571
FastDigest Esp3I (BsmBI)	ThermoFisher Scientific	Cat#FD0454
FastAP Thermosensitive Alkaline Phosphatase	ThermoFisher Scientific	Cat#EF0651
T4 Polynucleotide Kinase	New England Biolabs	Cat#M0201S
T4 DNA Ligase Reaction Buffer	New England Biolabs	Cat#B0202S
Quick Ligation™ Kit	New England Biolabs	Cat#M2200S
In-fusion snap assembly master mix	Takara Bio	Cat#638948
eBioscience™ fixable viability dye eFluor 780	ThermoFisher Scientific	Cat#65-0865-14
Belzer UW® cold storage transplant solution	Bridge to Life	Cat#BTLBUW-1000
Corning® Matrigel® matrix	Corning	Cat#356234
Corning® collagen I, rat tail	Corning	Cat#354236
HyClone™ sodium bicarbonate	Cytiva	Cat#SH30033.01
Sodium pyruvate	Sigma-Aldrich	Cat#S8636
Collagenase type IV	Sigma-Aldrich	Cat#C4-22-1G
UltraComp eBeads™ compensation beads	Invitrogen	Cat#01-2222-42
UltraComp eBeads™ plus compensation beads	Invitrogen	Cat#01-3333-42
eBioscience™ Foxp3 / transcription factor staining buffer set	Invitrogen	Cat#00-5523-00
Brilliant stain buffer plus	BD Biosciences	Cat#566385
ArC™ amine reactive compensation bead kit	Invitrogen	Cat#A10346
Human TruStain FcX™ (Fc receptor blocking solution)	BioLegend	Cat#422302
Liberase TL	Sigma-Aldrich	Cat#5401020001
Histopaque^(R)^-1119	Sigma-Aldrich	Cat#11191
Critical commercial assays		
NucleoSpin® RNA/protein kit	Macherey-Nagel	Cat#740933
NucleoSpin® RNA kit	Macherey-Nagel	Cat#740955
NucleoBond® xtra maxi columns	Macherey-Nagel	Cat#740414
NucleoSpin® plasmid quickpure kit	Macherey-Nagel	Cat#740615
Pierce™ BCA protein assay kit	ThermoFisher Scientific	Cat#A65453
Qubit® protein assay kit	ThermoFisher Scientific	Cat#Q33211
Qubit® dsDNA HS assay kit	ThermoFisher Scientific	Cat#Q32854
Qubit® dsDNA BR assay kit	ThermoFisher Scientific	Cat#Q32853
High-Capacity cDNA reverse transcription kit	ThermoFisher Scientific	Cat#4374967
CyQUANT™ cell proliferation assay, for cells in culture	ThermoFisher Scientific	Cat#C7026
NEBNext® rRNA depletion kit (human/mouse/rat)	New England Biolabs	Cat#E6310
NEBNext® Ultra II directional RNA library prep kit for Illumina	New England Biolabs	Cat#E7760
NGS fragment kit (1-6000bp), 1000	Agilent Technologies	Cat#DNF-473-1000
Tumor cell dissociation kit, human	Miltenyi Biotec	Cat#130-095-929
CD8^+^ T cell isolation kit	Miltenyi Biotec	Cat#130-096-495
ELISpot plus: human IFN-γ HRP	Mabtech	Cat#3420-4HST
CBA human IFN-γ enhanced sensitivity flex set	BD Biosciences	Cat#561515
CellTrace™ Far Red cell proliferation kit	ThermoFisher Scientific	Cat#C34564
Quick ligation kit	New England Biolabs	Cat#M2200
Chromium next GEM single-cell 5’ kit v2	10x Genomics	Cat#PN-1000263
SMARTer® Mouse TCR a/b Profiling Kit	Takara Bio	634403
MiSeq Reagent Kit v2 2 × 250 bp, 500 cycles	Illumina	MS-102-2003
Deposited data		
Raw western blot images	This paper	Zenodo: 10.5281/zenodo.10599407
Raw confocal microscopy images	This paper	Zenodo: 10.5281/zenodo.10599407
Raw bulk RNA-seq data from human cell lines and bulk TCR-seq data from mice	This paper	BioProject: PRJNA1263526
Raw bulk RNA-seq and single-cell RNA/TCR-seq data from human patient-derived fragments (PDFs)	This paper	EGA: EGAC50000000725
Raw proteomics data	This paper	PRIDE: PXD063528
Raw immunopeptidomics data	This paper	PRIDE: PXD073256
Processed sequencing data	This paper	Zenodo: 10.5281/zenodo.10599407
Processed proteomics data	This paper	Zenodo: 10.5281/zenodo.10599407
Processed immunopeptidomics data	This paper	Zenodo: 10.5281/zenodo.10599407
Processed source data used to generate the figures	This paper	Zenodo: 10.5281/zenodo.10599407
TCGA (expression data)	GDC portal	https://portal.gdc.cancer.gov/
GTEx (expression data)	GDC portal	https://portal.gdc.cancer.gov/
CPI1000+ cohort (RNA, exome and clinical data)	Litchfield et al.^9^	DOI: 10.1016/j.cell.2021.01.002
Experimental models: Cell lines		
A375	The Francis Crick Institute	RRID: CVCL_0132
A549	The Francis Crick Institute	RRID: CVCL_0023
HCT116	The Francis Crick Institute	RRID: CVCL_0291
HEK293T	The Francis Crick Institute	RRID: CVCL_0063
LL/2 (LLC1)	The Francis Crick Institute	RRID: CVCL_4358
MCF-7	The Francis Crick Institute	RRID: CVCL_0031
MDA-MB-157	The Francis Crick Institute	RRID: CVCL_0618
PC-9	The Francis Crick Institute	RRID: CVCL_B260
SK-MES-1	The Francis Crick Institute	RRID: CVCL_0630
NY-ESO-1 PBL cells	Laboratory of Martin Pule, UCL Cancer Institute, London, UK	N/A
Jurkat CD8^+^TCR^–/–^	Laboratory of Martin Pule, UCL Cancer Institute, London, UK	N/A
Experimental models: Organisms/strains		
C57BL/6J	The Francis Crick Institute	RRID: IMSR_JAX:000664
Recombinant DNA		
hAAVS1 1L TALEN	Addgene	Cat#35431; RRID: Addgene_35431
hAAVS1 1R TALEN	Addgene	Cat#35432; RRID: Addgene_35432
pAAVS-EGFP^PTC-35^	Zhu et al.^62^; Laboratory of Joshua Mendell, University of Texas Southwestern Medical Center, Dallas, TX, USA	DOI: 10.1016/j.celrep.2020.107895
pAAVS-EGFP^PTC-231^	Zhu et al.^62^; Laboratory of Joshua Mendell, University of Texas Southwestern Medical Center, Dallas, TX, USA	DOI: 10.1016/j.celrep.2020.107895
pCMV-VSV-G	Addgene	Cat#8454; RRID: Addgene_8454
pCMV-dR8.2 dvpr	Addgene	Cat#8455; RRID: Addgene_8455
pLVX-IRES-ZsGreen1	Takara Bio	Cat#632187
pLVX-CMV-CTAG1B (NY-ESO1)-IRES-ZsGreen plasmid DNA	Laboratory of Manuel Varas, San Sebastian University, Chile	N/A
pLVX-CMV-Stuffer-IRES-ZsGreen_empty plasmid DNA	Laboratory of Manuel Varas, San Sebastian University, Chile	N/A
pLentiNYE-EGFP^NMD+^	This study	N/A
pLentiNYE-EGFP^NMD−^	This study	N/A
Retroviral SFG.mR	Phillip et al.^138^; Laboratory of Martin Pule, UCL Cancer Institute, London, UK	DOI: 10.1182/blood-2014-01-545020
RDF (RD114, envelope expression plasmid)	Cosset et al.^139^; Laboratory of Martin Pule, UCL Cancer Institute, London, UK	DOI: 10.1128/JVI.69.12.7430- 7436.1995
pEQ-Pam3-E (Gag-pol expression plasmid)	Mille^140^; Laboratory of Martin Pule, UCL Cancer Institute, London, UK	DOI: 10.1002/0471142905.hg1205s80
pCRISPRv2	Sanjana et al.^141^ Addgene	Cat#52961; RRID: Addgene_52961
Software and algorithms		
FlowJo V10.10.0	BD Biosciences	https://www.flowjo.com/solutions/flowjo/downloads
GraphPad Prism 10 V10.4.2 (534)	Dotmatics	https://www.graphpad.com/
FACS diva v9.1	BD Biosciences	https://www.bdbiosciences.com/en-gb/products/software/instrument-software/bd-facsdiva-software
Sony ID7000 Software V2.02.17121	Sony Biotechnology	Installation mirror of the UCL flow cytometry translational technology platform
Original code	This paper	GitHub: https://github.com/Lab-TIGI-UCL/NMDi_promotes_cancer_neoantigens_2026_Immunity
Peptide-PRISM	Erhard et al^142^	https://erhard-lab.de/software
FragPipe v21.1	Kong et al.^143^; Ferreira et al.^144^	https://github.com/Nesvilab/FragPipe/releases
RStudio v2024.12.1+563	N/A	https://cran.rstudio.com
R V4.2.2	N/A	https://www.r-project.org/
biomaRt R package V2.46.3	Durinck et al.^145^	https://bioconductor.org/packages/release/bioc/html/biomaRt.html
Platypus V0.8.1	Rimmer et al.^146^	https://github.com/andyrimmer/Platypus
Annovar v2016Feb01	Wang et al.^147^	https://annovar.openbioinformatics.org/en/latest/
GenomicRanges V2.4.0	Lawrence et al.^148^	https://bioconductor.org/packages/release/bioc/html/GenomicRanges.html
LiftOver	Nassar et al.^149^	https://genome.ucsc.edu/cgi-bin/hgLiftOver
Rsamtools v1.40.0	Bioconductor	https://bioconductor.org/packages/release/bioc/html/Rsamtools.html
pHeatmap v1.0.12	Bioconductor	https://cran.r-project.org/web/packages/pheatmap/pheatmap.pdf
CellRanger Single-Cell Software Suite v7.1.0, Dec 7, 2022	10X Genomics	https://www.10xgenomics.com/support/software/cell-ranger/latest
Seurat v4.3.0	Hao et al.^150^	https://satijalab.org/seurat/
SeuratData v0.2.2	Hao et al.^150^	https://satijalab.org/seurat/articles/extensions
Sctransform v0.3.5	Hao et al.^150^	https://satijalab.org/seurat/articles/sctransform_vignette.html
Harmony v0.1.1	Broad Institute	https://portals.broadinstitute.org/harmony/
scRepertoire v1.8.0	Borcherding et al.^151^	https://www.bioconductor.org/packages/release/bioc/html/scRepertoire.html
DoubletFinder v2.0.4	McGinnis et al.^152^	https://github.com/chris-mcginnis-ucsf/DoubletFinder
MAST v1.24.1	Finak et al.^153^	https://www.bioconductor.org/packages/release/bioc/html/MAST.html
Hdf5r v1.3.8	Novartis	https://github.com/Novartis/hdf5r
FastQC v0.11.9	Babraham Institute	https://www.bioinformatics.babraham.ac.uk/projects/fastqc/
Trim Galore v0.6.10	Babraham Institute	https://www.bioinformatics.babraham.ac.uk/projects/trim_galore/
RSEM v1.3.3	Haas et al.^154^	http://deweylab.github.io/RSEM/
STAR v2.7.9a	Dobin et al.^155^	https://github.com/alexdobin/STAR
StringTie v2.1.7	Pertea et al.^156^	https://ccb.jhu.edu/software/stringtie/
Kallisto v0.46.1	Bray et al.^157^	https://pachterlab.github.io/kallisto/about
DESeq2 v1.45.0	Love et al.^158^	https://github.com/thelovelab/DESeq2/
Sva V3.53.0	Leek et al.^159^,^160^	https://bioconductor.org/packages/release/bioc/html/sva.html
IsoformSwitchAnalyzeR v1.18.0 (cell lines), V2.5.0 (explants)	Vitting-Seerup et al.^161^	https://github.com/kvittingseerup/IsoformSwitchAnalyzeR
Rtracklayer V1.65.0	Bioconductor	https://bioconductor.org/packages/release/bioc/html/rtracklayer.html
ReactomePA V1.50.0	Yu et al.^162^	https://github.com/YuLab-SMU/ReactomePA
clusterProfiler V4.13.4	Xu et al.^163^; Yu^164^	https://github.com/YuLab-SMU/clusterProfiler
Enrichplot V1.25.3	N/A	https://github.com/YuLab-SMU/enrichplot
organism (org.Hs.eg.db) V3.20.0	Bioconductor	https://bioconductor.org/packages/release/data/annotation/html/org.Hs.eg.db.html
VennDiagram V1.7.3	Chen and Boutros^165^	https://github.com/uclahs-cds/package-VennDiagram
UpSetR V1.4.0	Conway et al.^166^	https://github.com/hms-dbmi/UpSetR/
Mixcr V4.7.0	MiLaboratories Inc	https://mixcr.com/
Immunarch V0.9.1	N/A	https://immunarch.com/
Bio.SeqIO module (BioPython)	Cock et al.^167^	https://biopython.org/docs/1.76/api/Bio.SeqIO.html
Statannotations	N/A	https://pypi.org/project/statannotations/
NetMHCpan v4.1	Reynisson et al.^100^	https://services.healthtech.dtu.dk/services/NetMHCpan-4.1/
Python	N/A	https://www.python.org/downloads/
BSgenome.Hsapiens. UCSC.hg19 V1.4.3	Bioconductor	https://bioconductor.org/packages/release/data/annotation/html/BSgenome.Hsapiens.UCSC.hg19.html
BSgenome.Hsapiens. UCSC.hg38 V1.4.5	Bioconductor	https://bioconductor.org/packages/release/data/annotation/html/BSgenome.Hsapiens.UCSC.hg38.html
ComplexHeatmap	Gu et al.^168^	https://github.com/jokergoo/ComplexHeatmap
Circlize	Gu et al.^169^	https://github.com/jokergoo/circlize
Prosit	Gessulat et al.^170^; Weller et al.^51^	https://github.com/kusterlab/prosit/
ProteomicsDB	Schmidt et al.^171^	https://www.proteomicsdb.org/
MSstats v4.0	Kohler et al.^172^	https://github.com/Vitek-Lab/MSstats
MaxQuant V2.2.0	Cox et al.^173^	https://www.maxquant.org
Other		
Sep-pak tC18 96-well	Waters	Cat#186002321
Ultra-micro SpinColumn™, C18	Harvard Apparatus	Cat#BVD-74-7206
Falcon™ 8-well culture slide	Corning	Cat#354118
BioPureSPN PROTO C18 MACRO™ MiniSpin Columns	The Nest Group	Cat#HMM S18V

### Experimental Model and Subject Details

#### Cell lines and culture conditions

All lines were grown in 5% CO_2_ at 37 °C. A375, A549, HCT116, HEK293T, LLC1, MCF-7, MDA-MB-157, PC-9, and SK-MES-1 were obtained from the Francis Crick Institute Cell Science platform, authenticated using short tandem repeat (STR) profiling, and regularly tested for contamination for Mycoplasma (for human cell lines) and for mouse pathogens (for LLC1 cells). Jurkat CD8^+^ and TCR^-/−^ were obtained from Dr. Hans Strauss. Healthy donor peripheral blood mononuclear cells (HD-PBMCs) were obtained from blood cones purchased from Tebu-Bio. Gender of the patients from whom the cell cultures were derived is as follows (female: F; male: M): A375 (F), A549 (M), HCT116 (M), HEK293T (F), Jurkat (M), MCF-7 (F), MDA-MB-157 (F), PC-9 (M), and SK-MES-1 (M). No clinical information can be disclosed for blood cones. LLC1 cells derive from the lung of a male C57BL mouse bearing a tumor resulting from the implantation of primary Lewis lung carcinoma.

PC-9, A549, Jurkat CD8^+^, and Jurkat TCR^-/−^ were grown in Roswell Park Memorial Institute Medium (RPMI) 1640-glutamax (Gibco, Invitrogen) supplemented with 10% fetal bovine serum (FBS, Gibco, Invitrogen) and 100 U/mL penicillin-streptomycin (P/S, Gibco, Invitrogen). MDA-MB-157 were grown in RPMI 1640-glutamax (Gibco, Invitrogen) supplemented with 20% FBS (Gibco, Invitrogen) and 100 U/mL P/S (Gibco, Invitrogen). A375, HCT116, HEK293T, LLC1, and MCF-7 were grown in Dulbecco’s Modified Eagle’s Medium (DMEM)-glutamax (Gibco, Invitrogen) supplemented with 10% FBS (Gibco, Invitrogen) and 100 U/mL P/S (Gibco, Invitrogen). SK-MES-1 cells were grown in DMEM-glutamax (Gibco, Invitrogen) supplemented with 10% FBS (Gibco, Invitrogen), 100 U/mL P/S (Gibco, Invitrogen), 2 mM Glutamine (Gibco, Invitrogen) and 1% non-essential amino acids (NEAA, Gibco, Invitrogen). HD-PBMCs and the derived peripheral blood CD8^+^ T cells were grown in RPMI 1640-glutamax (Gibco, Invitrogen) supplemented with 10% human serum (from human male AB plasma, Sigma-Aldrich), 100 U/mL P/S (Gibco, Invitrogen), and 100 U/mL of Proleukin (IL-2, aldesleukin, Clingen).

#### Patient-derived fragments (PDFs) generation and culture conditions

Fresh human samples from patients diagnosed with CRC were collected from University College London Hospitals via the UCL/ UCLH Biobank (REC reference 20/YH/0088). Tumors and normal tumor-adjacent tissue were obtained from surgical resections and processed within 24 hours. All patients provided written consent for the research use of their tissue samples that were not required for diagnostic purposes. Detailed patient characteristics are provided in Table S2. Solid tumor lesions and matched adjacent normal tissue were macroscopically selected by a pathologist from the resected tumor material. Part of the tumor and normal tissue were collected in ice-cold collection medium (University of Wisconsin Solution [UW Solution, Bridge to Life] supplemented with 100 μg/mL of Primocin [InvivoGen]) for subsequent PDF cultures. All normal tissues were matched to tumor tissues used in the experiment, with the exception of CRC_019. Tissue materials collected for subsequent PDF cultures were immediately processed by manual resection into small tumor fragments of 1–2 mm^3^ size, on ice. After processing, tumor fragments from different regions within a tumor were mixed to minimize tumor heterogeneity and were frozen in cryovials containing 1 mL of 90% FBS (Gibco, Invitrogen) and 10% dimethyl sulfoxide (DMSO) (Sigma-Aldrich) with 15 tumor fragments per vial. All vials of PDFs were cryopreserved in liquid nitrogen until further usage.

For culture, PDFs were embedded in extracellular matrix (ECM) containing sodium bicarbonate (7.5% [Cytiva]), collagen I (1 mg/mL final concentration [Corning]), Matrigel (4 mg/mL final concentration [Corning]), and tumor medium. The ECM was prepared in the sequence of the materials listed above and was kept cold on ice throughout the process to avoid solidification. The tumor medium was made with DMEM, 1 mM of sodium pyruvate (Sigma-Aldrich), 1× MEM nonessential amino acids (Sigma-Aldrich), 1× Glutamax (Gibco, Invitrogen), 10% FBS, and 100 U/mL penicillin-streptomycin. Before PDF embedding, 40 μL of ECM were added to each well of a 96-well plate to be used. The ECM was solidified in a 37 °C incubator for at least 30 minutes. To thaw cryopreserved PDFs, vials were thawed in a 37 °C water bath until only a sliver of ice remained. Tumor fragments were then transferred to a 50 mL conical tube, and warm wash medium (DMEM, 10% FBS, and 1% P/S) was added dropwise up to 10 mL to gradient wash off DMSO. Afterwards, the tumor fragments were placed on a cell strainer (100 μm) in a 6-well plate and serially washed three times with 7 mL of wash medium in each well. After washing, one fragment was placed on the top of each solidified ECM-filled well, and an additional layer of 40 μL of ECM was added on top. The plate was then incubated again for at least 30 minutes for subsequent treatment. After ECM solidification, 120 μL of tumor medium were added on top of the ECM-embedded PDF.

#### Patient-derived organoids

Patients were recruited in the Tracking Cancer Evolution through Therapy (TRACERx) clinical study, which was approved by an independent research ethics committee (13/LO/1546; https://clinicaltrials.gov/ct2/show/NCT01888601). Written informed consent was obtained from all patients. Tumor tissue was obtained from a patient undergoing surgical resection of a primary NSCLC. The patient is also known under the study identifier CRUK1398 (male). Tissue was immediately transported on ice from the operating theatre to a pathology laboratory, where the tumor was sampled. Tumor tissue was collected in Ad-DF+++ medium (Advanced DMEM/F12 [Gibco, Invitrogen] supplemented with 2 mM UltraGlutamine I [Lonza], 10 mM HEPES [Gibco, Invitrogen], and 100 U/ml Penicillin/Streptomycin [Gibco, Invitrogen]), further supplemented with 1x Primocin (InvivoGen) and stored on ice. Samples were processed for organoid establishment within 24 hours of resection.

All plasticware was first coated by rinsing with a 1% FBS/ phosphate buffer saline (PBS, Gibco, Invitrogen) solution to prevent tumor cells or fragments sticking to plastics. Tumor tissue was washed in PBS, dissected into small fragments using surgical scalpels, and resuspended in 2.3 mL digestion buffer (Tumor Dissociation Kit, Miltenyi Biotec). Tumor tissue was digested following the manufacturer’s instructions for 60 minutes on a gentleMACS system. Digested tumor fragments were passed through a 70 μm strainer and pelleted by centrifugation (300 × *g*, 4 minutes, room temperature). If the cell pellet was red, cells were incubated for 5 minutes in red blood cell lysis buffer (8.26 g/L ammonium chloride, 1 g/L sodium bicarbonate, 0.1 Mm EDTA in distilled water) at room temperature. Cells were washed twice with PBS and pelleted by centrifugation. Establishment of tumor organoids was performed using previously published methods.^107,174^ Briefly, tumor cells from processed tissue were resuspended in ice-cold basement membrane extract (BME; Geltrex LDEV-free reduced growth factor basement membrane matrix [Gibco, Invitrogen]) diluted 2 : 1 with DMEM-F12 supplemented with 100 U/ml penicillin/streptomycin. After solidification of BME (20 minutes at 37 °C), tumor organoids were overlaid with minimal basic medium (MBM)^175^ to prevent outgrowth of normal airway organoids. MBM consists of DMEM/F12, supplemented with 100 U/ml penicillin/streptomycin, 1x B27 supplement (Gibco, Invitrogen), 1x N-2 supplement (Gibco, Invitrogen), 50 ng/mL human recombinant EGF (PeproTech), 20 ng/mL human recombinant FGF-2 (PeproTech), and 10 μM Y-27632 (Selleckchem). Organoids were passaged by isolating organoids from BME by washing with cold PBS, followed by dissociation with Accutase (Gibco, Invitrogen) for 3–10 minutes at 37 °C. Single cells or small cell clusters were reseeded in BME and cultured as above. Organoids were authenticated using STR profiling and regularly tested for Mycoplasma contamination.

#### Mice and housing conditions

C57BL/6J mice were bred and maintained under specific pathogen-free (SPF) conditions at the animal facility of the Francis Crick Institute. Animals were housed in ventilated cages with unlimited access to food (2018 Autoclavable Rodent Breeding Diet, ENVIGO RMS UK LTD, T.2018S.12) and water. Mice were littermates and co-housed for a minimum of 2 weeks before experiments. For experiments, mice at 8-12 weeks of age and weighing at least 20 grams were used. Males were used for the tumor challenge, as xenografts of LLC1 cells in female mice resulted in severe ulceration. Mice from the same litter were randomly assigned to treatment or control groups. Animal experiments were performed in accordance with national and institutional guidelines for animal care and were approved by the Francis Crick Institute Biological Research Facility Strategic Oversight Committee, incorporating the Animal Welfare and Ethical Review Body, conforming with UK Home Office guidelines and regulations under the Animals (Scientific Procedures) Act 1986, including Amendment Regulations 2012.

#### Healthy donor-derived model antigen CD8^+^ T cells

1G4 TCR-expressing T cells were kindly provided by Angeliki Karamani, Manar Shafat, Gordon Weung-Kit Cheung, and Martin Pule. CD8^+^ T cells were enriched using a CD8^+^ T cell isolation kit (Miltenyi Biotec). T cells were expanded in 1–2 cycles of a rapid expansion protocol (REP),^176^ by stimulation with 30 ng/mL anti-CD3 (OKT-3, eBioscience), 3000 U/mL IL-2 (Proleukin, Clingen), and 1:100 40 Gy irradiated feeder cells from three pooled healthy donors (Tebu-Bio). After two weeks of REP, cells were rested for 1 week in T cell medium (RPMI 1640 medium [Gibco, Invitrogen] supplemented with 10% human serum and 100 U/mL penicillin/streptomycin) with 100 U/mL IL-2 before cryopreservation. T cells were cultured in T cell medium with 100 U/mL IL-2.

### Method Details

#### Analysis of GTEx, TCGA, and CPI1000+ datasets

Expression data for GTEx and TCGA were downloaded from the GDC portal across all tissues and cancer types. Ensembl transcript IDs (https://www.ensembl.org/info/genome/stable_ids/index.html) were converted to gene IDs using the biomaRt package (2.46.3), and the transcripts per million (TPM) data were normalized to log_2_(TPM+1). The expressed means of each of the NMD genes were calculated across both cohorts.

Exome and transcriptome data from the CPI1000+ cohort were obtained from Litchfield et al.,^9^ in which a cohort of 1,008 CPI-treated patients was harmonized through a standardized bioinformatics pipeline. The outputs of this pipeline included TPM expression data, mutation status, and TMB. Further details on the processing, analysis, and cohorts can be found in the original paper. In the CPI1000+ cohort, a uniform clinical endpoint of response was defined across the studies based (“responder” is defined as a RECIST-criteria-based radiological response with complete response [CR] or partial response [PR], and “non-responder” is defined as stable disease [SD] or progressive disease [PD]).

#### NMD-score calculation

To measure the function of the NMD pathway, we developed a metric to assess sample-level NMD pathway activity (NMD-score). Nine TCGA cohorts (BRCA, COAD, HNSC, KIRC, LUAD, LUSC, OV, PRAD, and SKCM) and the CPI1000+ cohort were included. For germline variant calling, we first identified homozygous and heterozygous single nucleotide polymorphisms (SNPs) from BAM files using Platypus (v0.8.1)^146^ and annotated the variants using Annovar (v2016Feb01).^147^ hg38 was used as the reference genome. Annotated germline alterations were then filtered using the Genome Aggregation Database (gnomAD).^177^ For somatic variations, MAF files containing somatic mutations for the TCGA cohorts were directly downloaded from cBioPortal (https://www.cbioportal.org), and somatic mutations for the CPI1000+ cohort were called as previously described in Litchfield et al.^9^ Somatic mutations annotated using hg19 were converted to hg38-based ones using LiftOver.^149^ For all variants, coverage of read counts was extracted using Rsamtools (v1.40.0, https://bioconductor.org/packages/release/bioc/html/Rsamtools.html), and somatic and germline genetic alterations were validated using GenomicRanges (v2.4.0).^148^ Genes with RNA expression measured in Fragments Per Kilobase of transcript per Million (FPKM) = 0 were excluded. For each sample, the NMD-score was calculated as 1 - (number of expressed PTC mutations at the RNA level detected in both DNA and RNA / number of PTC mutations detected in DNA, or DNA and RNA). For the pan-cancer analysis, a linear regression model was used to adjust for tumor type and TMB, with Student’s *t* test applied.

#### Calculation of expressed PTCs and CPI response

The number of expressed PTCs was first log_2_-transformed and then compared between responders and non-responders in the CPI1000+ dataset, both pan-cancer and across individual cancer types. For the pan-cancer analysis, a linear regression model was used to adjust for tumor type and TMB, with Student’s *t* test applied.

#### Scoring of genes related to the NMD pathway

To identify an optimal therapeutic target within the NMD pathway, we compiled multiple sources: (I) Nonsense-Mediated Decay (NMD) Reactome pathway (*n* = 117, R-HSA-927802)^61^ and (II) genes with a significant MAGeCK score (*p* < 0.05) in a genome-wide CRISPR screen (*n* = 92).^62^ The two lists were merged, thereby obtaining a list of 195 unique genes. Ribosomal genes were then removed, resulting in a list of 109 candidate genes. Next, a scoring system to rank NMD pathway genes based on a wide number of biological and clinically relevant parameters was established: (1) presence in the NMD peer-reviewed Reactome pathway database (R-HSA-927802),^61^ (2) significant MAGeCK score in a recent NMD CRISPR screen,^62^ (3) positive scoring (*p* < 0.05) in an *in silico* protein-truncating variant (PTV, nonsense mutations and fs-indels) screen for genes significantly associated with lower NMD-score, (4) positive scoring (*p* < 0.05) in an *in silico* SCNA loss screen, (5) SCNA amplification screen for significant (*p* < 0.05) gene associations with NMD-score, (6) gene expression with a significantly (*p* < 0.05) positive association with NMD-score, (7) patients with PTV mutations of the gene resulting in significantly higher CPI response rate, (8) gene expression with a significant (*p* < 0.05) negative association with Danaher’s CD8^+^ scores,^178^ and (9) availability of therapeutic agents targeting the protein product of the gene (source OpenTarget v23.12 https://platform.opentargets.org/).^179^ One point was assigned to each gene when it scored in any of these parameters. Genes were then ranked in descending order based on their summed scores. Genes with a total score ≥ 3 were visualized (*n* = 50, Figure 1C).

#### Cell transfection and chemical inhibition

Cells were transfected with Lipofectamine RNAiMax (ThermoFisher Scientific) according to the manufacturer’s instructions with 30 nM of ON-TARGETplus siRNA Pools (Dharmacon) targeting SMG1 (23049), UPF1 (5976), or a non-targeting pool. EGFP-NMD reporter cell lines were generated as described in Zhu et al.^62^ Briefly, plasmids expressing TALEN pairs targeting the human AAVS1/ PPP1R12C locus (Addgene #35431 and #35432)^180^ and pAAVS-EGFP^PTC-231^ or pAAVS-EGFP^PTC-35^ (a kind gift from Prof. Joshua T. Mendell) were co-transfected into A375 and PC-9 cells using Lipofectamine^™^ 2000 (ThermoFisher Scientific) at a ratio of Left TALEN: Right TALEN: pAAVS-EGFP^PTC-231^ or pAAVS-EGFP^PTC-35^ of 1:1:5. Two days after transfection, selection with 400 μg/mL hygromycin (ThermoFisher Scientific) was initiated and maintained for 14 days. Single-cell clones were then derived and screened for reporter fluorescence.

SMG1 inhibition was achieved by using two different small-molecule inhibitors (obtained from Genesis Therapeutics) at 0.5 μM (SMG1i#1) and 1 μM (SMG1i#2) unless otherwise stated. Rapamycin (MedChemExpress) was used at 100 nM and indisulam (Sigma-Aldrich) at 1 μM.

#### Cell growth assays

For proliferation assays upon SMG1 knockdown, 6 × 10^3^/well cells were plated in 96-well plates. The following day, cells were transfected with siSMG1 or non-targeting siRNAs and cultured for 72 hours. Four images per well were taken at 2-hour intervals using an IncuCyte ZOOM system (Essen BioScience). The percentage of cell confluency was measured and analyzed with the IncuCyte ZOOM software.

For proliferation assays upon SMG1 chemical inhibition, 1 × 10^3^/well (A375) or 0.5 × 10^3^/well (PC-9) cells were plated in 8 different 96-well plates. The following day, cells were treated with DMSO, SMG1i#1, or SMG1i#2 at the appropriate concentrations. Every day (including day 0, after cells attached to the plate and before treatment, as a plating reference), one plate was taken from the incubator, the media was gently removed, and cells were frozen at -80 °C. Once all plates were collected, cell proliferation was measured using the CyQUANT Cell Proliferation Assay for cells in culture (ThermoFisher Scientific) according to the manufacturer’s instructions. Luminescence was measured using a Varioskan LUX (ThermoFisher Scientific).

#### RNA and protein extraction, real-time quantitative polymerase chain reaction (RT-qPCR), and western blot

RNA and proteins were extracted with the Nucleospin RNA/protein or RNA kit (Macherey-Nagel) according to the manufacturer’s instructions. 1 μg of RNA was reverse transcribed into complementary DNA (cDNA) using the High-Capacity complementary DNA Reverse Transcription Kit (ThermoFisher Scientific). RNA expression was measured by qPCR using Fast SYBR™ Green Master Mix (ThermoFisher Scientific) on a LightCycler 480 (Roche) and normalized using HPRT, TBP, and UBC as reference genes. Primer sequences can be found in Table S5. Western blotting experiments were performed using the following primary antibodies: tubulin (RRID: AB_477582, T6074, clone B-5-1-2, Sigma-Aldrich, 1:5,000), GAPDH (RRID: AB_307275, ab9485, Abcam, 1:1,000), SMG1 (RRID: AB_10860072, 9149S, clone D42D5, Cell Signaling Technology, 1:1,000), phospho-UPF1 (RRID: AB_10805931, Ser1127, 07-1016, Sigma-Aldrich, 1:1,000) and UPF1 (RRID: AB_2288326, A300-038A, Bethyl Lab, 1:10,000).

#### pUPF1 enzyme-linked immunosorbent assay (ELISA)

A549 cells were seeded at 5 × 10^4^ cells/well in a 96-well tissue culture-treated plate and allowed to adhere overnight at 37 °C. Cells were treated with compounds for 6 hours at 37 °C. Next, cells were lysed, and lysate was transferred to pre-coated and blocked anti-UPF1 plates (RRID: AB_2288326, Bethyl Lab, A300-038A) for an overnight incubation at 4 °C. Plates were washed, and phospho-ATM/ATR substrate motif (Cell Signaling, 2909) detection antibody was added and incubated for 3 hours at room temperature. Plates were washed, and HRP-linked anti-rabbit IgG antibody (Cell Signaling, 7074) was added and incubated for 1 hour at room temperature. Glo substrate (R&D Systems, DY993) was used to develop the signal, and plates were read on the Neo2 (Perkin Elmer) using luminescence mode after 10 minutes of incubation at room temperature.

#### DNA damage assessment

5 × 10^4^ A375 cells were seeded in 8-chamber culture slides (Corning) and treated with DMSO, SMG1i#1 (0.5 μM), SMG1i#2 (1 μM), siCtrl (30 nM), siSMG1 (30 nM) or cisplatin (2 μM) and cultured for 72 hours. Samples were then fixed with 4% paraformaldehyde (ThermoFisher Scientific) for 20 minutes and washed 3 times with PBS. Samples were blocked for 1 hour at room temperature with Goat Serum Dilution Buffer (GSDB; 450 mM NaCl, 12.75 mM sodium phosphate buffer, 16% goat serum, 0.3% Triton X-100). The samples were subsequently washed with 0.1% TBS-Tween and incubated with primary antibody overnight at 4 °C (γH2A.X, Cell Signalling Technology, #9718; 1/600 dilution in GSDB). Samples were then washed in 0.1% TBS-Tween, incubated with secondary antibody (Alexa Fluor 488, Invitrogen) and with DAPI (Invitrogen) in GSDB for 1 hour at room temperature. Samples were washed with 0.1% TBS-Tween and mounted with Vectashield (Vector Laboratories, H1200) before imaging. Images were acquired using a Zeiss AxioImager.M1 microscope with 40x 1.3NA Plan-Neofluar or 63x 1.4 Plan-Apochromat objectives, equipped with an Orca-Spark CMOS camera (Hamamatsu), pE300-white LED light source (CoolLED), and controlled by Micro-Manager 2.0 Software.^181^ Images were processed and counted using the FindFoci ImageJ plugin.^182^

#### RNA library preparation and NovaSeq sequencing

Initial RNA sample quality assessment, RNA library preparations, and sequencing were conducted at GENEWIZ. RNA samples were quantified using Qubit 4.0 Fluorometer (ThermoFisher Scientific), and RNA integrity was checked with an Agilent 5600 Fragment Analyzer (Agilent Technologies). RNA-seq library preparation was done using NEBNext rRNA Depletion Kit (Human/Mouse/Rat) and NEBNext Ultra II Directional RNA Library Prep Kit for Illumina following the manufacturer’s instructions (NEB). Briefly, rRNA was depleted with the NEBNext rRNA Depletion Kit (Human/Mouse/Rat) and fragmented. First-strand and second-strand cDNA were subsequently synthesized. The second strand of cDNA was marked by incorporating dUTP during the synthesis. cDNA fragments were adenylated at the 3’ ends, and an indexed adapter was ligated to cDNA fragments. Limited-cycle PCR was used for library amplification. The dUTP incorporated into the cDNA of the second strand enabled its specific degradation to maintain strand specificity. Sequencing libraries were validated using the DNA Kit on the Agilent 5600 Fragment Analyzer (Agilent Technologies) and quantified by using the Qubit 4.0 Fluorometer (Invitrogen, Carlsbad, CA). The sequencing libraries were multiplexed and clustered on the flowcell. After clustering, the flowcell was loaded on the Illumina NovaSeq 6000 instrument according to the manufacturer’s instructions. The samples were sequenced using a 2×150 Pair-End (PE) configuration. Image analysis and base calling were conducted by the NovaSeq Control Software (v1.6) on the NovaSeq instrument. Raw sequence data (.BCL files) generated from Illumina NovaSeq were converted into fastq files and de-multiplexed using the Illumina bcl2fastq program (v2.20). One mismatch was allowed for index sequence identification.

#### RNA-seq data pre-processing

FastQC (v0.11.9) was used to check sequence quality (https://www.bioinformatics.babraham.ac.uk/projects/fastqc/). The RNA-seq data of the PDFs were trimmed using Trim Galore (v0.6.10) before further processing (https://www.bioinformatics.babraham.ac.uk/projects/trim_galore/). The reads were aligned to the human reference genome (hg38) and assembled into potential transcripts using STAR (v2.7.9a)^155^ and StringTie (v2.1.7).^156^ GeneCode Human Release 104 (hg38)^183^ was used as the gene and transcript annotation source. RNA quantification was performed using Kallisto (v0.46.1)^157^ and RSEM (v1.3.3).^184^

#### IsoformSwitchAnalyzeR and DESeq2

Kallisto-quantified transcript abundance was used to identify isoform switching events with the IsoformSwitchAnalyzeR (v1.18.0 for cell lines) and (v2.5.0 for explants) package in R (v4.4.2).^161^ Before investigating isoform switching events in the PDFs RNA-seq data, the sva (v3.53.0)^159,160^ package was used to identify and build surrogate variables using the svaseq() function, which allowed for accounting for batch effects in the IsoformSwitchAnalyzeR pipeline. The IsoformSwitchAnalyzeR package embedded functions take kallisto quantification results as input, specifically the estimated counts and TPM values. The importIsoformExpression() function from ISAR utilizes edgeR (v4.3.0) to normalize counts for library size differences, thereby enabling the identification of isoform switches using the DEXSeq package (v1.51.0), along with the annotation of ORFs and PTCs. Isoform usage was calculated using the isoform fraction (IF), calculated as the fraction of isoform expression over gene expression. The IFs were next used to determine the ratio of IF (rIF) by calculating the fraction of IFs between a condition of interest and the control condition, which was used as a measure of the change in isoform expression in a condition of interest. The results were annotated based on the Comprehensive Gene Annotation data (GRCh38, release 41) covering the whole genome from GeneCode (https://www.gencodegenes.org/human/). Results were considered statistically significant if rIF was > 1.5 and false-discovery rate (FDR)-corrected *p* value (*q*) < 0.05. Several plots were created using packages in R: pie charts and box plots were generated using ggplot2 (v3.4.2) (https://ggplot2.tidyverse.org/), Venn diagrams were made using the VennDiagram (v1.7.3) package,^165^ and UpSet plots were created using the UpSetR (v1.4.0) package.^166^ Differential gene expression analysis was performed by importing the outputs of RSEM into R and using DESeq2 (v1.45.0).^158^ PCA plots were generated using an embedded function in the DESeq2 package.

#### RNA variant allele frequency (VAF) analysis

The RNA VAF was calculated for transcripts that are known to have PTC-producing mutations in the A375 and PC-9 cell lines, to study whether NMD inhibition increased the RNA VAF for these transcripts compared to the control condition. DNA mutations for these cell lines were downloaded from the DepMap portal (CCLE_DepMap_18q3_maf_20180718.txt, https://depmap.org/portal/download/all/). The DNA mutation file, Rsamtools (v1.40.0, https://bioconductor.org/packages/release/bioc/html/Rsamtools.html) and GenomicRanges (v2.4.0)^148^ packages were used to calculate the number of allelic reads in the RNA-seq data for each transcript expected to have a mutation (based on the DepMap mutation file). The VAF was then calculated for each mutation as the number of reads of the mutated allele over the total number of reads at that locus. The transcripts for which VAF was calculated were classified based on their mutation’s NMDetective score (NMDetectiveB_v2), which indicates the efficiency of NMD at degrading transcripts with a given PTC.^27^ Mutations associated with an NMDetective score < 0.25 were classified as NMD-insensitive, and those associated with a score ≥ 0.25 as NMD-sensitive. Transcripts with VAFs = 0.00 in all conditions were removed due to their low expression. Transcripts with VAFs > 0.75 in all conditions were also removed, as this pattern indicates that these transcripts were not degraded by NMD, even in the control conditions, but rather constitutively escaped NMD-mediated degradation and would represent a confounder in our analysis. This phenomenon is expected, given that even the most robust predictive model to date, developed by Lindeboom et al.^27^ and used in our analysis, can accurately predict ~75% of NMD-regulated events.

#### Gene Set Enrichment Analysis (GSEA)

Differential gene expression analysis was performed on the cell lines (A375 and PC-9) RNA-seq data (RSEM outputs) grouped by treatment (siSMG1, SMG1i#1, SMG1i#2) using DESeq2.^158^ Before running DESeq2, the sva (v3.53.0) package was used to identify and build surrogate variables using the svaseq() function, which allowed to account for batch effects in the differential expression analysis.^159,160^ The outputs from the differential expression analysis were filtered for significant (adjusted *p* < 0.05) differentially expressed protein-coding genes (based on GeneCode Human Release 104 (hg38) annotations)^183^ and used as inputs for the GSEA, which was performed per treatment in R using the gsePathway() function from the ReactomePA (v1.50.0) package and the cluster-Profiler (v4.13.4) package to convert gene symbols to Entrez IDs using the bitr() function.^162,185^ GSEA enrichment plots were made for the NMD enhanced by the EJC (R-HSA-975957) and NMD independent of the EJC (R-HSA-975956) pathways of the Reactome database using the gseaplot2() function from the enrichplot (v1.25.3) package in R.^186^

#### HLA binding prediction

Cell lines: to quantify the number of predicted strong-binding antigens upon SMG1i or SMG1 knockdown, significantly upregulated PTC-containing transcripts (defined as significantly upregulated if rIF ≥ 1.5 and *q* < 0.05 compared to the control condition (DMSO or siCtrl)) were used. *In silico* translation of neoORFs derived from PTC-containing transcripts was performed using the cDNA sequences from the GRCh38.104 genome assembly. For each significantly upregulated PTC-containing transcript, *in silico* translation was initiated from the annotated start codon and continued until a stop or nonsense codon was reached. If a start codon was unannotated (e.g., in retained intron transcripts), the start codon coordinates were taken from an overlapping protein-coding transcript.

The translated PTC-containing sequence was then compared to the translated sequences of all overlapping protein-coding isoforms using a pairwise alignment algorithm. Any PTC-containing sequences that exactly matched longer sections (≥ 6 residues) of a protein-coding isoform were subtracted, leaving behind a neoORF sequence unique to the PTC-containing transcript. The resulting peptide sequences were processed using a sliding window to generate 8-14-mer sequences, each containing ≥ 1 residue from the neo-ORF sequence, for downstream MHC-peptide binding prediction. NetMHCpan (v4.1)^100^ (with cell line-specific HLA alleles) was used to predict binding affinity scores. The harmonic mean of the scores of the best peptides presented on each HLA allele was used to create a parent sequence level presentation score. Strong binding was defined as a presentation score < 0.5. The raw number of presentable neoepitopes was divided by 32.102474 to get the number of presented epitopes per megabase (Mb). Cancers from the CPI cohort were defined as TMB-high if they had ≥ 10 mutations/Mb, and TMB-low otherwise.

Patient-derived fragments: HLA-HD (v1.6.0)^187^ was used to call HLA types from RNA, as no DNA-seq data were available. All replicates and conditions for each explant resulted in the same HLA types called. Transcripts were defined as significantly upregulated if they had rIF ≥ 1.5 and *q* < 0.05 compared to the control condition (DMSO). *In silico* translation was performed as described above. As DNA-seq data were unavailable, peptide identification was limited to those not detected in the DMSO control RNA-seq data. NetMHCpan (v4.1)^100^ was used to predict binding affinity scores. Peptides were defined as presentable if the best-ranking peptide had a percentile rank score of < 0.5.

#### Proteomics sample preparation

Cell pellets consisting of 1 × 10^7^ cells treated with siCtrl, siSMG1, DMSO, SMG1i#1, or SMG1i#2 for 72 hours were lysed in 500 μL urea lysis buffer (50 mM triethylammonium bicarbonate, 8 M urea, cOmplete Protease Inhibitor Cocktail (1:50 dilution; Roche, 11873580001), 1 PhosSTOP tablet (Roche, 4906845001), 1 mM sodium orthovanadate) and lysates sonicated until clear. Protein concentration was measured using a BCA protein assay (Pierce #23227). 40 μg of protein was reduced with 5 mM tris(2-carboxyethyl)phosphine hydrochloride (Sigma-Aldrich, C4706) at 37 °C for 20 minutes and alkylated using 10 mM 2-chloroacetamide (Sigma-Aldrich, 22790) for 20 minutes at room temperature in the dark. Peptides were digested with 400 ng LysC (FUJIFILM Wako, 129-02541) for 4 hours at 30 °C, then diluted with 50 mM triethylammonium bicarbonate (Sigma T7408) to reduce the urea concentration down to 1.6 M. Samples were further digested with 800 ng Trypsin (Promega, V5113) for 4 hours at 37 °C.Digest reactions were quenched by the addition of 10% trifluoroacetic acid (Sigma-Aldrich, 302031) to a final pH of 2. Sample desalting was performed using 35-350 μg C18 columns (The Nest Group, Inc., HMM S18V) according to the manufacturer’s specifications. Dried samples were stored at -80 °C and resuspended in 1% formic acid immediately before LC-MS/MS analysis.

#### Proteomics LC-MS/MS

NanoLC-MS/MS was performed on an Orbitrap Exploris 480 coupled to an Easy-nLC 1200 (ThermoFisher Scientific) liquid chromatograph. 2 μg of each sample was analyzed as 2 μL injections. Peptides were loaded on a 25 cm (75 μm ID, 1.7 μm 120 Å pore size C18 resin) Aurora Ultimate UHPLC packed emitter column (Ion Opticks) housed in a Nanospray Flex Ion Source modified to include a column oven (Sonation GmbH) set to 35 °C. Peptides were separated using a linear gradient from 6% to 38% (buffer A: 0.1% formic acid in water, buffer B: 80% acetonitrile/0.1% formic acid) over either 180 minutes (A375) or 240 minutes (PC-9), at a flow rate of 250 nL/minute. Peptides were ionized by electrospray ionization using 1.8 kV applied immediately before the analytical column via a microtee built into the nanospray source with the ion transfer tube heated to 320 °C and the RF-lens set to 45%. Precursor ions were measured in a data-dependent mode in the Orbitrap analyzer scanning between 375–1200 m/z at a resolution of 120,000 and a normalized AGC target of 300% (3 × 10^6^ ions) (max. MS1 injection time set to “Auto”). With the duty cycle fixed to 3 seconds, precursors were subjected to MS/MS fragmentation under the following criteria: Monoisotopic Peak Determination set to “Peptide”, Intensity threshold set to 1 × 10^5^, Charge states set to “2–5”. MS/MS scans were generated using HCD fragmentation with the collision energy set to 30% and measured in the Orbitrap at a resolution of 15,000 with the first mass set to m/z = 120 and the normalized AGC target of 75% (7.5 × 10^4^ ions) (max. MS2 injection time set to “Auto”).

#### Proteomics data analysis

Raw data were analyzed with MaxQuant (v2.2.0)^173^ where they were searched against the human SwissProt database (http://www.uniprot.org/, downloaded 27/07/2023) using default settings. Carbamidomethylation of cysteines was set as a fixed modification, and oxidation of methionines and acetylation at protein N-termini were set as variable modifications. Enzyme specificity was set to trypsin with maximally 2 missed cleavages allowed. To ensure high-confidence identifications, peptide-spectral matches, peptides, and proteins were filtered at a less than 1% FDR. Label-free quantification in MaxQuant was used with an LFQ minimum ratio count of 2, Fast LFQ selected, and the ‘skip normalization’ option selected. The ‘match between runs’ feature was selected with a match time window of 42 seconds and an alignment time window of 20 minutes. For statistical analysis, the ‘proteinGroups.txt’ and ‘evidence.txt’ output files from MaxQuant were loaded into the MSstats statistical framework package (v4.0)^172^ run in R. Contaminants and reverse sequences were removed, data were log_2_-transformed, normalized by the global median, and a linear mixed-effects model was fitted to the data. The group comparison function was employed to test for differential abundance between conditions. p-values were adjusted to control the FDR using the Benjamini-Hochberg procedure.^188^

#### HLA-peptidomics sample preparation

HLA purification was done as previously described in Kalaora et al.^189^ Briefly, cell pellets consisting of 2 × 10^8^ cells treated with DMSO, SMG1i#1, or SMG1i#2 for 72 hours were homogenized and lysed with lysis buffer (containing 0.25% sodium deoxycholate, 0.2 mM iodoacetamide, 1 mM EDTA, protease inhibitor cocktail (Sigma-Aldrich), 1 mM PMSF and 1% octyl-β-D-glucopyranoside in PBS) and incubated at 4 °C for 1 hour. Lysates were cleared by centrifugation at 4 °C, 48,000 × *g* for 45 minutes, and passed through a pre-clearing column containing Protein A Sepharose beads (GenScript). HLA-peptide complexes were then immunoaffinity purified from the cleared lysate using pan-HLA antibody (W6/32 antibody purified from HB95 hybridoma cells), covalently bound to Protein A Sepharose beads. The HLA-peptide complexes were eluted with 1% trifluoracetic acid (TFA), followed by purification of the peptides by Sep-Pak tC18 100 mg Sorbent 96-well plate (Waters). Elution of the peptides was done with 28% acetonitrile (ACN) in 0.1% TFA.

#### HLA-peptidomics LC-MS/MS analysis

The HLA peptides were dried by vacuum centrifugation, resolubilized with 0.1% TFA and 5 mM TCEP, and separated using reversedphase chromatography using the nanoElute2 system (Bruker), with the Aurora C18 nano column (75 μm × 250 mm, IonOptiks, Australia), mobile phase A: H_2_O + 0.1% formic acid, B: acetonitrile + 0.1% formic acid. The HLA peptides were separated with a linear gradient over 80 minutes from 2 to 29%, 28 to 95% in 0.5 minutes, maintained at 95% for 2.9 minutes, and back to initial conditions, at a flow rate of 0.3 μL/minute. The chromatography system was coupled with a captive spray nanoESI to a quadrupole-ion mobility-time-of-flight mass spectrometer (TIMS-ToF Pro, Bruker). The MS was operated in data-dependent acquisition mode (DDA). Mass range of 100-1,700 Th. The Ion Mobility range was set to start at ion mobility 0.6–1.57 1/K0, ramp time 300 milliseconds. Target intensity was set to 20,000 with a threshold of 2,500.

#### HLA-peptidomics computational analysis

HLA class-I peptidomics dataset contains eighteen raw files corresponding to two cell lines, A375 and PC-9 (nine files for each cell line). Each set is divided into SMG1i#1 and SMG1i#2 drug treatments and DMSO control. Three biological replicates were prepared for each treatment. This dataset was analyzed by MaxQuant,^173^ and the search engine used was Andromeda, integrated within MaxQuant (v2.1.3.0). The sequence database contained the non-canonical (*i.e*., *in silico*-translated peptides derived from PTC-containing transcripts identified by RNA-seq) and the canonical human proteome, obtained from Ensembl GRCh38 and the UniProt database^190^ after removal of 100% sequence redundancy using CD-HIT.^191^ The maximum allowed precursor mass tolerance was 20 ppm. N-terminal acetylation and methionine oxidation were set as variable modifications. A peptide spectrum match FDR of 0.05 was used, and no protein FDR was set. Enzyme specificity was set as ‘unspecific’. The obtained peptides were filtered by multiple criteria: Peptides with MaxQuant scores less than or equal to 60 or PEP larger than 0.1 were discarded. Any peptide not predicted by NetMHCpan (v4.1)^100^ to bind the relevant HLA-alleles (either as strong < 0.5% rank or weak < 2% rank binders) was removed. MaxQuant ‘by MS/MS’ hits were obtained, and peptides showing MS/MS % fragmentation coverage lower than or equal to 60% were filtered out (fragmentation coverage was calculated as the number of fragmented ions (0 or 1) between two amino acids along the peptide sequence length -1 and multiplied by 100). Alternative ambiguous peptide matching the MS/MS was examined based on the Delta score of MaxQuant. Peptides were discarded in cases where the next best matching MS/MS of MaxQuant score is 20 or less relative to the best matching score. Furthermore, isobaric AA ambiguity was analyzed by substituting each Leu with Ile (and vice versa) in all possible combinations along the peptide sequence and comparing the derived sequence against the canonical human database. Pseudogenes were checked against the output provided in (http://www.pseudogene.org/Human/Human90.txt). Furthermore, peptides were compared against long noncoding transcripts obtained from GeneCode (v38).^183^ Additionally, peptides were compared against the IEDB^192^ and the NuORF databases^53^ (Table 1). Finally, to account for cancer specificity, the HLA Ligand Atlas^193^ was re-analyzed with PRISM (to increase the speed and efficiency of the search),^142^ including the non-canonical customized reference database described above, to remove any peptide previously identified in normal tissues. Histograms show the predicted NetMHCpan binding rank of all peptides identified in DMSO-treated and SMG1-treated samples. Peptides with rank < 2 and restricted to 8- to 14-mers were considered as predicted binders. Predicted binders were then further filtered by RNA-seq differentially expressed genes. Pie plots illustrate percentages of drug-specific, isoform-specific identified peptides arising from different types of transcripts. Heatmaps show predicted peptides in drug-specific, shared, and DMSO-specific peptides, respectively. IGV was used to plot RNA-seq coverage of representative drug-induced, candidate neoepitopes. All analyses were performed using R (v4.2.2).

#### HLA-peptidomics spectrum validation using light synthetic peptides

Light synthetic peptides for spectra validation were ordered from GenScript, as HPLC grade (≥ 85% purity). These were analyzed using the same LC–MS/MS system and acquisition parameters as indicated below for the endogenous peptides, with the following changes: the gradient was set from 4% to 30% acetonitrile in 20 minutes, and NCE was set to 27. The data were processed with MaxQuant using the following parameters: all FDRs were set to 1, and the individual peptide mass tolerance was set to false. MaxQuant spectra from endogenous and synthetic runs were correlated against each other using Prosit,^170^ as described in Weller et al.,^51^ mirror plots were plotted on proteomicsdb (https://www.proteomicsdb.org/use/?fragment_tol=10&fragment_tol_unit=ppm&matching_tol=10&matching_tol_unit=ppm).

#### Immunogenicity assessment for peptides identified in HLA-peptidomics

The protocol was adapted from Ali et al.^104^ PBMCs were isolated on day -4 from fresh blood of healthy donors with HLA-matched alleles. 5-10 × 10^7^ PBMCs were subjected to CD14 separation (with CD14 microbeads, Miltenyi Biotec). The CD14^−^ cell fraction was cryopreserved in freezing media (10% DMSO – 90% FBS) on day -4 and thawed on day -1 in the presence of DNase I (10 μg/mL, Sigma-Aldrich) to isolate naïve CD8^+^ T cells (with CD8^+^ T Cell Isolation Kit in conjunction with CD45RO and CD57 microbeads, Miltenyi Biotec). CD14^+^ monocytes were grown and differentiated into immature monocyte-derived dendritic cells (MoDCs) for three days in X-VIVO 15 (Lonza) media supplemented with 5% human AB serum (Sigma-Aldrich), 100 U/mL P/S (Gibco, Invitrogen), GM-CSF (800 IU/mL, PeproTech), and IL-4 (50 IU/mL, PeproTech). On day -1, DCs were matured by the addition of a maturation cocktail containing 10 ng/mL lipopolysaccharides (LPS from *Escherichia coli* O111:B4, [Sigma-Aldrich]) and 100 ng/mL IFNγ (PeproTech) for 16 hours. On day 0, mature monocyte-derived DCs were pulsed with the corresponding synthetic peptides or DMSO at a concentration of 1 μg/mL for 2 hours and subsequently co-cultured with autologous T cells for 12 days. On days 3, 5, 7, and 9, IL-7 (5 ng/mL, PeproTech) and IL-15 (5 ng/mL, PeproTech) were added to the culture media. On day 12, T cells were restimulated with MoDCs generated as above, pre-pulsed with the relevant peptides, and cultured for an additional 9 days. On day 21, cells were collected, washed twice in PBS (Gibco, Invitrogen), and resuspended to 1 × 10^6^ cells/mL. IFN-γ ELISpot was performed using ELISpot Plus: Human IFN-γ HRP (Mabtech) according to the manufacturer’s instructions. 5 × 10^4^ T cells/well were plated in 50 μL. 50 μL of peptide (1 μM)-containing media (X-VIVO 15 [Lonza] media supplemented with 5% human AB serum [Sigma-Aldrich] and 100 U/mL penicillin-streptomycin) was added, and the plate was incubated for 24 hours at 37 °C. Media without peptide or media containing phorbol myristate acetate (PMA; 50 ng/mL, Sigma-Aldrich) and ionomycin (1 μg/mL) served as negative and positive controls. The plate was then washed and stained according to the manufacturer’s instructions and imaged using Bioreader 7000-E (BioSys).

#### Cloning

NYE-EGFP-NMD reports: the EGFP and beta-globin sequences were lifted from the EGFP^PTC-231^ and EGFP^PTC-35^ plasmids^62^ and inserted into the pLVX-IRES-ZsGreen1 (Takara Bio) plasmid using In-Fusion Snap Assembly (Takara Bio) as per the manufacturer’s instructions. The sequence encoding for the NY-ESO-1 A*02:01-SLLMWITQC (NYE_157-165_) peptide was then cloned in-frame of the EGFP gene to obtain a single RNA transcript encoding for the NY-ESO-1 A*02:01 antigen, EGFP, and the beta-globin sequence degraded by the NMD pathway (pLentiNYE-EGFP^NMD+^) or escaping NMD pathway degradation (pLentiNYE-EGFP^NMD−^). Plasmids were transformed into recombination-deficient bacteria (Stbl3, ThermoFisher Scientific) and individual clones were screened by colony PCR and confirmed by Sanger sequencing.

CRISPR-Cas9 constructs: CRISPR-Cas9 cloning was performed as described in https://media.addgene.org/cms/filer_public/4f/ab/4fabc269-56e2-4ba5-92bd-09dc89c1e862/zhang_lenticrisprv2_and_lentiguide_oligo_cloning_protocol_1.pdf. Briefly, pLenti-CRISPRv2 backbone (Addgene #52961)^141^ was digested with BsmBI (ThermoFisher Scientific) and dephosphorylated with FastAP Thermosensitive Alkaline Phosphatase (ThermoFisher Scientific). Oligonucleotides encoding each sgRNAs with pLentiCRISPRv2-compatible overhangs were phosphorylated with T4 Polynucleotide Kinase (New England Biolabs) using T4 ligation buffer (New England Biolabs) and annealed as follows: 37 °C for 30 minutes, 95 °C for 5 minutes, followed by cooling down to 25 °C at 5 °C/minute. Annealed oligos were diluted 1:200 in water and ligated with Quick Ligase (New England Biolabs) into the digested vector. Ligated plasmids were transformed into recombination-deficient bacteria (Stbl3, ThermoFisher Scientific), and individual clones were screened by colony PCR and confirmed by Sanger sequencing across the U6-sgRNA cassette. Sequences of sgRNAs and sequencing primers are provided in Table S5.

#### Lentivirus production and viral transduction

HEK293T cells were transfected with the standard PEI method. Briefly, cells were plated in a 6 cm ∅ dish. When cells reached 90% confluency, they were transfected with the following transfection mix: 1 mL of Opti-MEM (Gibco, Invitrogen) containing 16.5 μL of PEI (1 mg/mL, pH 4.5, Polysciences), 1.25 μg of pCMV-ΔR8.2-dvpr (Addgene, #8455), 0.415 μg of pCMV VSV-G (Addgene, #8454), and 2.85 μg of pLentiNYE-EGFP^NMD+^ or pLentiNYE-EGFP^NMD−^ expressing the NY-ESO-1 A*02:01-SLLMWITQC (NYE 157-165) peptide or pLentiCRISPRv2 with different sgRNA sequences. 16 hours post-transfection media was replaced with DMEM Glutamax (Gibco, Invitrogen) supplemented with 20% FBS (Gibco, Invitrogen) and 100 U/mL P/S (Gibco, Invitrogen). Viral supernatant was collected 48 and 72 hours post-transfection. Supernatant from the 48 hour collection was kept at 4 °C for 24 hours and mixed with the 72 hour supernatant. The mix was then centrifuged at 300 × *g* for 5 minutes at room temperature, the supernatant was collected, avoiding contact with any visible pellet, and immediately filtered with a 0.45 μm low-adhesion filter (Merk Millipore) to remove any cell debris. Viral supernatant was either used fresh for lentiviral transduction or stored at -80 °C. Lentivirus produced in HEK293T cells was used to stably transduce A375, PC-9, HCT116 cells, or LUAD organoids. For established cell lines, 500 μL of fresh virus was added to a 6 cm ∅ dish containing 70% confluent cells in 2 mL of media supplemented with 10% FBS, 100 U/mL P/S, and 10 μg/mL polybrene (Sigma-Aldrich). For LUAD organoids, the organoids were dissociated to single cells with Accutase (Gibco, Invitrogen) and resuspended at 1 × 10^6^ cells/mL in DMEM/F12 medium supplemented with 100 U/mL P/S (Gibco, Invitrogen). Dissociated organoids were combined 1:1 with viral supernatant in the presence of 10 mg/mL polybrene (Sigma-Aldrich) and 10 mM Y-27632 and plated at 1 × 10^6^ cells/well in a 12-well plate, centrifuged for 1 hour at 100 × *g*, and incubated overnight at 37 °C. Tumor cells were washed in PBS and plated in BME for organoid formation. Successfully infected cells and organoids were selected in appropriate media supplemented with 1 μg/mL puromycin (InvivoGen) for 1 week. After selection, cells were sorted on a BD FACSAria™ Fusion Cell Sorter. EGFP-positive cells were collected and used for further experiments.

#### Flow cytometry for EGFP

To assess NMD impairment upon SMG1 inhibition/knockdown in cell lines bearing the EGFP^PTC-231^ and the EGFP^PTC-35^ reporters or the NYE-EGFP^NMD+^ and NYE-EGFP^NMD−^ reporters, cells and organoids were collected 72 hours (for EGFP^PTC-231^ and EGFP^PTC-35^ reporters) or 48 hours (for NYE-EGFP^NMD+^ and NYE-EGFP^NMD−^ reporters) after transfection/chemical inhibition. Cells were then washed twice with cold FACS buffer (PBS supplemented with 1% BSA [Sigma-Aldrich] and 2.5 mM EDTA [Sigma-Aldrich]) and passed through a 35 μm filter to obtain a single-cell suspension. Cells were then stained with eBioscience™ Fixable Viability Dye eFluor™ 780 (ThermoFisher Scientific, 1:1,000) and incubated at 4 °C in the dark for 30 minutes. Cells were then washed twice in FACS buffer and resuspended in FACS buffer for flow cytometry acquisition. All experiments were run on a BD LSRFortessa™ X-20 Cell Analyzer.

#### Cloning of TCR sequences into retroviral vectors

Retroviral SFG.mR vectors were kindly gifted by Dr. Martin Pule. The SFG plasmid backbone contains a sort-suicide gene, RQR8,^138^ as a surrogate marker for TCR expression. The TCR DNA sequences (described in Bethune et al.,^105^ Table S5) were cloned into the SFG.mR.RQR8 vector as an NcoI/MluI fragment. TCR DNA sequences were ordered as gBlocks (IDT) encoding both the TCR α and β chain, separated by an E2A peptide. The synthetic TCR was designed to include the human constant domain TRBC2. TRBC2 was codon-optimized and engineered to incorporate stabilizing murine residues. For cloning, both the TCR gBlock fragments and plasmid backbone were enzymatically digested with NcoI and MluI and ligated with Quick Ligase (New England Biolabs).

#### Retrovirus production and Jurkat and peripheral blood T cells transduction

HEK293T cells were seeded in 10 cm ∅ dishes the day before transfection with the viral products. RD114-pseudotyped γ-retroviral supernatants were generated by transfection of the HEK293T cells with an SFG vector plasmid, an RD114 or RDF envelope expression plasmid, and a Gag-pol expression plasmid or PeqPam-env. Cells were transfected with GeneJuice (Sigma-Aldrich) according to the manufacturer’s instructions. Viral supernatant was collected at 48 and 72 hours post-transfection. After collection of the 72-hour supernatant, the two timepoints were combined and sterile-filtered with a 0.45 μm filter.

Jurkat and peripheral blood CD8^+^ T cells were transduced as described in Thomas et al.^194^ Briefly, cells were split the day before transduction and kept at a concentration of 1 × 10^6^. On the day, viral supernatant was added to RetroNectin (Takara Bio)-coated 24-well plates. 1 × 10^6^ Jurkat cells or peripheral blood CD8^+^ T cells were added to the virus-containing wells. The plate was spun at 2,000 × *g* for 2 hours at 32 °C. After centrifugation, the plate was returned to the incubator. Transduction efficiency was determined as the percentage of RQR8^+^ cells by flow cytometry 48 hours post-transduction. In the case of peripheral blood CD8^+^ T cells, cells were additionally sorted for CD8^+^ expression. Transduced cells were used for downstream applications seven days posttransduction.

#### Co-culture experiments

For co-culture experiments, A375, PC-9, and HCT116 cells and LUAD PDTOs (stably transduced with either the pLentiNYE-EGFP^NMD+^ or pLentiNYE-EGFP^NMD−^ constructs) were collected 48 hours after transfection with siCtrl or siSMG1 or treatment with DMSO, SMG1i#1, or SMG1i#2 in the presence of IFN-γ (100 U/mL). Cells were dissociated to single cells and passed through a 40 μm strainer. 5 × 10^4^ tumor cells or PDTOs were plated with 1 × 10^5^ Jurkat or healthy donor peripheral blood CD8^+^ T cells (target : effector ratio 1 : 2) in 200 μL of RPMI supplemented with 10% FBS (Jurkat) or 10% HS (HD peripheral blood CD8^+^ T cells) and 100 U/mL P/S per well of a U-bottom 96-well plate. T cells without tumor cells were used as a negative control. Phorbol myristate acetate (PMA; 50 ng/mL, Sigma-Aldrich) and ionomycin (1 μg/mL) served as positive control. For MHC-I/MHC-II blocking experiments, HLA-ABC (MHC-I block, RRID: AB_2561492, clone W6/32, Biolegend, Ultra-LEAF™, 311427) or HLA-DR/DP/DQ (MHC-II block, RRID: AB_395938, clone Tü39; BD Biosciences, Ultra-LEAF™, 555556) blocking antibodies were added to the co-culture at a final concentration of 10 μg/mL.

24 hours after co-culture, T cells were collected, washed twice in FACS buffer (PBS supplemented with 1% BSA [Sigma-Aldrich] and 2.5 mM EDTA [Sigma-Aldrich]). Cells were then stained with BB700 mouse anti-Human CD3 (RRID: AB_2860004, clone SK7, BD Biosciences, 1:200), FITC mouse anti-human CD4 (RRID: AB_395751, clone RPA-T4, BD Biosciences, 1:20), BV421 mouse anti-Human CD8 (RRID: AB_11154035, clone RPA-T8, BD Biosciences, 1:200), BV650 mouse anti-human CD69 (to assess Jurkat T cell activation; RRID: AB_2738442, clone FN50, BD Biosciences, 1:100), PE mouse anti-human CD137 (to assess peripheral blood-derived CD8^+^ T cell activation; RRID: AB_398477, clone 4B4-1, BD Biosciences, 1:30), PE mouse anti-human TCRαβ (RRID: AB_2738921, clone IP26, BD Biosciences, 1:100), and eBioscience™ fixable viability dye eFluor™ 780 (ThermoFisher Scientific, 1:1,000) and incubated at 4 °C in the dark for 30 minutes. Cells were then washed twice in FACS buffer and resuspended in FACS buffer for flow cytometry. All experiments were run on a BD LSRFortessa™ X-20 Cell Analyzer.

#### Cell killing co-culture

For cell killing co-culture experiments, A375 stably transduced with either the pLentiNYE-EGFP^NMD+^ or pLentiNYE-EGFP^NMD−^ constructs were collected 24 hours after transfection with siCtrl or siSMG1 or treatment with DMSO, SMG1i#1, or SMG1i#2. 5 × 10^3^ tumor cells were stained with CellTrace Far Red Proliferation Kit according to the manufacturer’s instructions (final concentration 1 μM), washed, plated in 200 μL of DMEM supplemented with 10% FBS and 100 U/mL P/S per well of a flat-bottom 96-well plate, and placed in a tissue culture incubator to adhere. 24 hours after plating, the media was removed, and 1 × 10^4^ CD8^+^-sorted peripheral blood CD8^+^ T cells stably transduced with the NY-ESO-1-specific TCR (1G4) were added to each well in RPMI supplemented with 10% AB HS, 100 U/mL P/S, and 100 U/mL of IL-2. The plate was then placed in an Incucyte SX5 equipped with the Green-Orange-NIR module and scanned every hour for 36 hours.

#### Flow cytometry analysis of PDFs

For T cell activation and phenotypic evaluation, PDFs were analyzed by high-dimensional flow cytometry. PDFs were manually retrieved from the ECM 96 hours after treatment. PDFs were pooled for each condition and processed into single-cell suspensions by enzymatic digestion on a rotator at 37 °C for 1 hour with a digestion mixture RPMI 1640 (Gibco, Invitrogen) supplemented with 1 mg/mL of collagenase type IV (Sigma-Aldrich) and 25.2 μg/mL of DNase I (Sigma-Aldrich). Samples were subsequently washed with ice-cold PBS, followed by manual mashing through a 100 μm filter (Miltenyi Biotec).

The following antibodies were used for flow cytometry analysis of the PDFs: BUV496 mouse anti-human CD8 (RRID: AB_2870223, clone RPA-T8, BD Biosciences, 1:80), BUV563 mouse anti-Human CD45RA (RRID: AB_2870211, clone: HI100, BD Biosciences, 1:80), BUV805 mouse anti-human CD3 (RRID: AB_2870181, clone: SK7, BD Biosciences, 1:40), BB790-P anti-human CD4 (RRID: N/A, clone: SK3, BD Custom Conjugates, 1:160), Brilliant Violet 421™ mouse anti-human CD279 (PD-1) (RRID: AB_10960742, clone: SK3, BioLegend, 1:20), Brilliant Violet 650™ mouse anti-human CD197 (CCR7) (RRID: AB_2563867, clone: G043H7, BioLegend, 1:10), Brilliant Violet 785™ mouse anti-human CD45 (RRID: AB_2563129, clone: HI30, BioLegend, 1:20), APC rat anti-human Foxp3 (RRID: AB_1603280, clone: PCH101, Invitrogen, 1:40).

For chemokine receptor staining, cells were incubated with 25 μL of antibody mixes containing CCR7 antibody (BioLegend) with Human TruStain FcX™ Fc Receptor Blocking Solution (BioLegend) at RT in the dark for 20 minutes. In the meantime, antibody mixes were prepared in Brilliant Staining Buffer Plus (BD Biosciences) and FACS buffer (PBS containing 2% FBS and 2 mM EDTA) for subsequent extracellular staining. Next, CCR7-stained cell suspensions were supplemented with 25 μL of antibody mix containing the aforementioned extracellular antibodies diluted in FACS buffer and incubated for 30 minutes at 4 °C in the dark. For all samples, eBioscience Fixable Viability Dye eFluor 780 (1:1,000 dilution, ThermoFisher Scientific) was used for the extracellular staining step for dead cell exclusion. Next, cells were washed twice, fixed, and permeabilized using Foxp3 Transcription Factor Staining Buffer set (ThermoFisher Scientific) according to the manufacturer’s instructions and incubated for 30 minutes at room temperature in the dark. For intracellular staining, cells were washed twice and resuspended in 50 μL of intracellular antibody staining mix with the aforementioned antibodies (intracellular antibodies only) and incubated for 30 minutes at 4 °C in the dark. Samples were washed thrice before acquisition.

Data acquisition was carried out on a BD FACSymphony A5 (BD Biosciences). Data were collected using BD FACS Diva Software (BD Biosciences) and further analyzed with FlowJo (v10.9.0) and GraphPad Prism (v10.0.2). An example of the gating strategy is shown in Figure S3C.

#### Cytometric Bead Array (CBA)

Supernatants from the PDFs were collected 96 hours after treatment, right before fragment dissociation, and stored at -80 °C until all patients’ supernatants were collected. Supernatants were then thawed, and Human IFN-γ Enhanced Sensitivity Flex Set (BD Biosciences) was performed according to the manufacturer’s instructions. All experiments were run on a BD LSRFortessa™ X-20 Cell Analyzer.

#### scRNA/TCR-seq sample preparation

PDTF were treated with either DMSO or SMG1i#2 for 96 hours. Single-cell suspensions were then prepared as described above, and cells were sorted. Subsequently, cells were washed twice in PBS 0.04% BSA, counted, and resuspended to a concentration of 1 × 10^3^ cells/μL in PBS 0.04% BSA. Cells were then loaded and processed on the 10X Chromium Controller using the CG000331 Chromium Next GEM Single Cell 5’ Reagent Kits v2 (Dual Index), which enabled us to process the gene expression and immune profiling of the samples. Once the libraries were processed, they were pooled with a GEX 4:1 TCR ratio (as per 10X recommendation) and submitted for sequencing. Each library can be recognized by its unique sample index. Sample sequencing was performed at Novogene, using a Novaseq 6000 platform and PE150 sequencing strategy.

#### scRNA/TCR-seq analysis

Sequenced reads were aligned using the CellRanger Single-Cell Software Suite (v7.1.0, Dec 7, 2022) against the hg38 reference genome. Data processing of scRNA-seq data was conducted using R, and the foundational analysis pipeline was established using the Seurat package (4.3.0).^150^ Seurat objects of the control and drug group were created, and cells with fewer than 400 expressed genes were excluded from the analysis. Next, cells with a percentage of mitochondrial RNAs of more than 10% were discarded. RNA expression was log-normalized using the scale factor of 10,000. Cells were clustered and annotated into T cells (*CD3D, CD3E*), NK cells (*NKG7*), B cells (*CD79A, MS4A1*), plasma cells (*IGKC, JCHAIN*), macrophages (*TYROBP, FCER1G*), and monocytes (*CST3*). T cells were further clustered into helper T cells (IL7R), effector T cells (*CD8A, GZMA*), exhausted T cells (*CXCL13, LAG3, TIGIT*), naïve T cells (*TCF7, CCR7*), and regulatory T cells (*FOXP3, IL2RA*) (Table S3). To quantify the level of tumor-antigen specificity and exhaustion of CD8^+^ T cells in the tumor microenvironment, the AddModuleScore() function embedded in the Seurat package was utilized to calculate the NeoTCR8 score and exhaustion score.^82,83^ The genes used for calculating the NeoTCR8 (*n* = 243) and the exhaustion (*n* = 90) scores are listed in Table S4. TCR clonotypes were called using ScRepertoire.^151^ TCR clones with a copy number greater than 5 in each sample were used as candidate libraries. TCR clonotypes were defined rare if the relative proportion occupied by those clones was less than 0.0001, small if the relative proportion occupied by those clones was between 0.0001 and 0.001, medium if the relative proportion occupied by those clones was between 0.001 and 0.01, large if the relative proportion occupied by those clones was between 0.01 and 0.1, and hyperexpanded if the relative proportion occupied by those clones was between 0.1 and 1. The relative abundances of different clonotypes were compared between treatments (DMSO, SMG1i#2) within each T cell group (effector and exhausted). Additionally, the relative abundance of TCRs that were shared between the SMG1i#2 and the DMSO group was also compared in the two T cell groups.

#### Mouse treatment

On the day of injection, LLC1 cells were collected, passed through a 70 μm cell strainer to obtain a single-cell suspension, washed 3× in PBS and counted. Cells were resuspended in PBS at a concentration of 2.5 × 10^6^ live cells, and 100 μL of cell suspension was injected into the right flank of 8-12 weeks of age male mice weighing at least 20 grams, using a 25G needle, which was changed after every injection. Seven days later, when tumor volume was between 50 and 100 mm^3^, mice were randomly assigned to treatment cohorts. For treatments, KVS0001 was dissolved in DMSO at a concentration of 120 mg/mL and stored at -80 °C. On the day of treatment, KVS0001 and DMSO were resuspended in 20% SBE-β-CD (Cambridge Bioscience) (5% KVS0001 or DMSO, 95% solvent). KVS0001 and DMSO were administered via intraperitoneal injection daily, Monday-Friday, 30 mg/kg. Anti-mouse PD-1 (CD279, clone RMP-1-14, InVivoMAb, BioXCell) was diluted at a working concentration of 1 mg/mL and administered via intraperitoneal injection (100μg/dose, q3 [3 injections every third day]). 3-4 times per week, mice were weighed, and tumors were measured. Tumor volume was calculated using the following formula: tumor volume = L × l × l × 0.52 (π/6); where L is the major length and l the minor length. Tumors were collected either on day 9 post-treatment (for high-dimensional flow cytometry experiments) or when ethical limits were reached (i.e., ulceration or tumor volume between 1.5 and 2 cm^3^). After sacrifice, tumors were immediately removed and washed in PBS and either snap-frozen in liquid nitrogen for RNA extraction or dissociated for flow cytometry.

#### Mouse tumor dissociation

On day 9 after treatment initiation, tumors were excised and transferred into plain RPMI 1640 Medium (Gibco, Invitrogen) immediately following culling. To increase digestion efficiency, the tumors were physically dissociated into small fragments approximately 3-4 mm^3^ in size. Fragments were then incubated at 37 °C in 1 mL of digestion mix (plain RPMI (Gibco, Invitrogen), Liberase TL (0.33 mg/mL, Sigma-Aldrich), and DNase I (75 μg/mL, Sigma-Aldrich) on a rocking platform at 800 RPM for 30 minutes. 500 μL plain RPMI was added to the digestion mix, which was then poured onto a 70 μm MACS SmartStrainers (Miltenyi Biotec). Residual tissue on the strainer was gently mashed using a syringe plunger and flushed with FACS buffer (PBS, 2% BSA, 2 mM EDTA). This step was performed twice, and the filtered cell suspension was collected in 15 mL centrifuge tubes and centrifuged at 450 × *g* for 3 minutes twice. The resulting pellets were then resuspended in complete RPMI (RPMI 1640, 10% FBS, 100 U/mL Penicillin/Streptomycin) and underlaid with 3 mL Histopaque-1119 (Sigma-Aldrich) using a glass Pasteur pipette. The processed content was then centrifuged at 800 × *g* for 10 minutes at room temperature with a brake-free setting. The buffy coat was carefully taken out, transferred to a new 15 mL centrifuge tube.

#### High-dimensional spectral flow cytometry for mouse tumors

Cells were washed with FACS buffer and centrifuged at 450 × *g* for 5 minutes twice and incubated at room temperature for 10 minutes with 60 μL in-house-made Superblock (PBS [Gibco, Invitrogen], 5% mouse serum [Sigma-Aldrich], 5% rat serum [Sigma-Aldrich], 5% rabbit serum [Sigma-Aldrich], 2% FBS [Gibco, Invitrogen], rat anti-mouse CD16/32 [final concentration: 25 μg/mL, BioLegend]). The cells were topped up with FACS buffer to 200 μL and centrifuged at 450 × *g* for 5 minutes. The wash was repeated twice.

To perform extracellular antigen staining, in a 50 μL per sample staining volume system with Brilliant Stain Buffer Plus (BD Biosciences), antibody mix was prepared as follows: BUV395 mouse anti-mouse NK-1.1 antibody (dilutions: 1:250, clone: PK136, BD Biosciences, RRID: AB_2738618), BUV496 rat anti-mouse CD4 antibody (dilutions: 1:250, clone: GK1.5, BD Biosciences, RRID: AB_2722549), BUV563 rat anti-mouse CD45 antibody (dilutions: 1:250, clone: 30-F11, BD Biosciences, RRID: AB_2722550), BUV661 rat anti-CD11b antibody (dilutions: 1:400, clone: M1/70, BD Biosciences, RRID: AB_2870249), BUV737 rat anti-mouse CD3 antibody (dilutions: 1:250, clone: 17A2, BD Biosciences, RRID: AB_2738781), BUV805 rat anti-mouse CD8a antibody (dilutions: 1:250, clone: 53-6.7, BD Biosciences, RRID: AB_2870186), BV510 rat anti-mouse CD357 (GITR) antibody (dilutions: 1:100, clone: DTA-1, BD Biosciences, RRID: AB_2739945), BV711 hamster anti-mouse CD137 (4-1BB) antibody (dilutions: 1:200, clone: 17B5, BD Biosciences, RRID: N/A), BV786 rat anti-mouse CD134 (OX40) antibody (dilutions: 1:100, clone: OX86, BD Biosciences, RRID: AB_2740573), FITC rat anti-mouse CD25 antibody (dilutions: 1:100, clone: PC61, BioLegend, RRID: AB_312854), PE/Dazzle 594 rat anti-mouse CD279 (PD-1) antibody (dilutions: 1:100, clone: 29F.1A12, BioLegend, RRID: AB_2566006), PE/Cyanine7 hamster anti-human/mouse/rat CD278 (ICOS) antibody (dilutions: 1:150, clone: C398.4A, BioLegend, RRID: AB_10641839), eFluor780 Fixable Viability Dye (LIVE/DEAD) (dilutions: 1:1000, Invitrogen, RRID: N/A), 10% Superblock, and remaining volume was topped up with FACS buffer. Cells were incubated at 4 °C in the dark for 30 minutes, then resuspended in 200 μL of FACS buffer. The suspension was centrifuged at 450 × *g* for 5 minutes, and this washing step was repeated thrice. Cells were then permeabilized and fixed with 200 μL of permeabilization and fixation buffer using the Foxp3 Transcription Factor Staining Buffer Set (Invitrogen) at room temperature in the dark for 30 minutes. Fixed cells were then centrifuged at 800 × *g* for 5 minutes and resuspended in permeabilisation buffer. This wash step was repeated thrice before intracellular staining. For intracellular and intranuclear antigen and transcription factor staining, in a 50 μL per sample staining volume system with permeabilisation buffer, antibody mix was prepared as follows, BUV496 rat anti-mouse CD4 antibody (dilutions: 1:250, clone: GK1.5, BD Biosciences, RRID: AB_2722549), BUV737 rat anti-mouse CD3 antibody (dilutions: 1:250, clone: 17A2, BD Biosciences, RRID: AB_2738781), BUV805 rat anti-mouse CD8a antibody (dilutions: 1:250, clone: 53-6.7, BD Biosciences, RRID: AB_2870186), eFluor450 rat anti-mouse FOXP3 antibody (dilutions: 1:100, clone: FJK-16s, Invitrogen, RRID: AB_1518812), APC mouse anti-human/mouse Granzyme B antibody (dilutions: 1:100, clone: GB11, Invitrogen, RRID: AB_2536539), AF700 rat anti-human/mouse/rat Ki-67 antibody (dilutions: 1:200, clone: SolA15, Invitrogen, RRID: AB_2637479). Untreated samples were used as unstained controls and were used to extract the autofluorescence (AF) profile. All single stain controls were prepared in the same manner and underwent the same permeabilization and fixation procedure as the samples to ensure consistency with the AF fluorescent profile. Acquisition was performed on a Sony ID7000 Spectral Analyzer (Sony Biotechnology). Unmixing was performed on the instruments and exported as unmixed FCS files. FCS files were further analyzed using FlowJo (BD Bioscience). An example of the gating strategy is shown in Figure S4F.

#### Mouse TCR-seq

For TCR-seq, 16 LLC1 tumor xenografts of mice treated with DMSO, KVS0001, DMSO+αPD-1, or KVS0001+anti-PD-1 (4 mice per treatment) when tumors reached 1.5-2 cm^3^ were used. Total RNA was extracted with the Nucleospin RNA kit (Macherey-Nagel) according to the manufacturer’s instructions, and cDNA libraries were prepared using the SMARTer^(R)^ Mouse TCR a/b Profiling Kit (Takara Bio). Bulk TCR-seq was performed on an Illumina MiSeq platform using the Illumina MiSeq Reagent Kit v2 (500 cycles, TCR-beta chain, paired-end reads, 2 x 250bp configuration). Raw reads were aligned and assembled into TCR-beta CDR3 sequences using MiXCR (v4.7.0) and its preset for the SMARTer^(R)^ Mouse TCR a/b Profiling Kit, with an adjustment to assemble clonotypes by the CDR3 region.^195^ The immunarch (v.0.9.1; https://immunarch.com/, https://github.com/immunomind/immunarch) package was used for downstream analyses in R. The unique number of clonotypes was calculated using the repExplore() function, and the relative abundance of clonotypes was computed using the repClonality() function. To measure repertoire similarity between all sample pairs, the repOverlap() function was applied using the “public” clonotypes method.

#### Quantification and Statistical Analysis

Details regarding data presentation and statistical information are provided in the figure legends. All flow cytometry data were analyzed with FlowJo (v10.10.0). All statistical analyses were performed and visualized in RStudio (v2024.12.1+563; R v4.4.2) and GraphPad Prism 10 (v10.6.1, 799) for macOS. Final figures were generated using Adobe Illustrator 2026 (v30.1).

Bar plots represent the mean ± s.e.m.; for boxplots, boxes denote the interquartile range, the black line represents the median, and whiskers extend to 1.5× the interquartile range. For statistical significance, GraphPad Prism notation for *p*-values was used: ns, *p* > 0.05; *, *p* ≤ 0.05; **, *p* ≤ 0.01; ***, *p* ≤ 0.001; ****, *p* ≤ 0.0001.

## Supplementary Material

Supplementary Materials

Table S1

Table S4

## Figures and Tables

**Table 1:** List of aberrant peptides identified in the immunopeptidomics experiments

Gene Ensembl	Transcript classification	Peptide sequence	Predicted HLA binding	Predicted binding strength	*In vitro* priming validation	IEDB HLA allele DB	HLA-ligand atlas normal	nuORF Riboseq	Unique in genome	Treatments	Cell line	FDR5 %	FDR1 %	# of DMSO replicates detected	# of SMG1i#1 replicates detected	# of SMG1i#2 replicates detected
DDX20	NMD	SSDPSLIGVY	HLA-A*01:01	strong	yes	no	no	yes	yes	both	A375	yes	yes	1/3 (intensity change)	3/3	3/3
ALG8	NMD	ILGPEAFSDV	HLA-A*02:06	weak	N/A	no	no	yes	yes	SMG1i#2	PC-9	yes	yes	0/3	0/3	1/3
RPS12	intron retention	VYQSQYCGF	HLA-A*24:02	strong	yes	no	no	yes	yes	SMG1i#2	PC-9	yes	yes	0/3	0/3	1/3
SLC44A2	intron retention	SASPTGPAL	HLA-B*07:02; HLA-B*55:02	strong	no	no	no	yes	yes	SMG1i#2	PC-9	yes	yes	0/3	0/3	1/3
RPS12	intron retention	EFMVLAQKF	HLA-A*24:02	strong	yes	no	no	yes	yes	both	PC-9	yes	yes	0/3	3/3	3/3
UCHL1	processed transcript	AAVSPHGPA	HLA-B*55:02	weak	N/A	no	no	yes	yes	both	PC-9	yes	yes	0/3	1/3	2/3
H2AC25	missense mutation	GLLPNIQAV	HLA-A*02:01	strong	yes	no	no	N/A	yes	both	A375	yes	no	2/3 (intensity change)	2/3	3/3
SEC24C	missense mutation	VTETSVFFY	HLA-A*01:01	strong	no	no	no	N/A	yes	both	A375	yes	no	0/3	2/3	1/3

HLA, human leukocytes antigens; IEDB, immune epitope database; nuORF, novel or unannotated open reading frame; FDR, false discovery rate. Related to Figure 5.

## Data Availability

Data from public repositories were accessed as follows: TCGA and GTEx exome and transcriptome data are accessible at the Genomic Data Commons Data Portal (https://portal.gdc.cancer.gov). MAF files containing somatic mutations for the TCGA cohorts were directly downloaded from cBioPortal (https://www.cbioportal.org). CPI1000+ exome and transcriptome data are publicly accessible (10.1016/j.cell.2021.01.002). Raw bulk RNA-seq data from cell lines and bulk TCR-seq data from mice have been deposited into the NCBI Sequence Read Archive (Bio-Project: PRJNA1263526) and are publicly available as of the date of publication. Raw bulk RNA-seq data and scRNA/TCR-seq data from human PDFs used during this study have been deposited with the European Genome-phenome Archive (EGA), which is hosted by the European Bioinformatics Institute (EBI) and the Center for Genomic Regulation (CRG) (EGA: EGAC50000000725). Details regarding applications for access are available on the relevant EGA page. The mass spectrometry proteomics data have been deposited to the ProteomeXchange Consortium via the PRIDE^137^ partner repository (PRIDE: PXD063528, proteomics; and PRIDE: PXD073256, immunopeptidomics) and are publicly available as of the date of publication. All original western blot, microscopy images, and processed data used in the figures have been uploaded on Zenodo (Zenodo: 10.5281/zenodo.10599407) and are publicly available as of the date of publication. github.com/Lab-TIGI-UCL/NMDi_promotes_cancer_neoantigens_2026_ Immunity). The code used in this manuscript has been deposited into GitHub and is publicly available as of the date of publication (GitHub: https://github.com/Lab-TIGI-UCL/NMDi_promotes_cancer_neoantigens_2026_Immunity). Any additional information required to reanalyze the data reported in this study is available from the lead contact with a completed data transfer agreement (DTA).

## Highlights

- NMD decay restricts access to a large pool of neoantigens
- NMD inhibition induces MHC class I presentation of immunogenic non-canonical peptides
- Targeting NMD promotes T cell activation and increases checkpoint blockade efficacy
- Rigorous framework for NMD-target discovery and NMD-derived antigen identification

## In brief

Vendramin et al. identify nonsense-mediated mRNA decay (NMD) as a central constraint on tumor antigenicity. Inhibiting the NMD kinase SMG1 stabilizes aberrant NMD-targeted transcripts and reveals a previously inaccessible class of non-canonical, non-mutational neoantigens that reshape the cancer immunopeptidome, drive antigen-dependent T cell responses, and increase checkpoint blockade efficacy.
