# Supplementary material for: Nonsense-mediated mRNA decay inhibition reshapes the cancer immunopeptidome
Source: Immunity. Author manuscript; Available in PMC 2026 Jun 13. (PMC7619149; doi:10.1016/j.immuni.2026.02.005)
Supplement: Supplementary Materials [file EMS213923-suppement-Supplementary_Materials.pdf]

CellPress
OPEN ACCESS

SUPPLEMENTAL INFORMATION

Supplemental information can be found online at https://doi.org/10.1016/j.immuni.2026.02.005.

Received: August 12, 2025
Revised: December 10, 2025
Accepted: February 9, 2026

Immunity, Volume *59*

Supplemental information

Nonsense-mediated mRNA decay inhibition

reshapes the cancer immunopeptidome

Roberto Vendramin, Hongchang Fu, Shanila Fernandez Patel, Yue Zhao, Danwen Qian, Lorena Ligammari, Osnat Bartok, Polina Greenberg, Ronen Levy, Andrea Castro, Krupa Thakkar, Jun Murai, Wei-Ting Lu, Christopher C.T. Sng, Chen Weller, Gordon Beattie, Amandeep Bhamra, Roc Farriol-Duran, Despoina Karagianni, Marcellus Augustine, Krijn K. Dijkstra, Christopher L. Pinder, Benjamin S. Simpson, Gordon Weng-Kit Cheung, TRACERx Consortium, Felipe Galvez-Cancino, Petra Vlckova, Silvia Surinova, Manuel Rodriguez-Justo, Mansi Shah, Nicholas McGranahan, Jeremy G. Carlton, Eva Grönroos, James L. Reading, Yardena Samuels, Charles Swanton, Sergio A. Quezada, and Kevin Litchfield

Figure S1

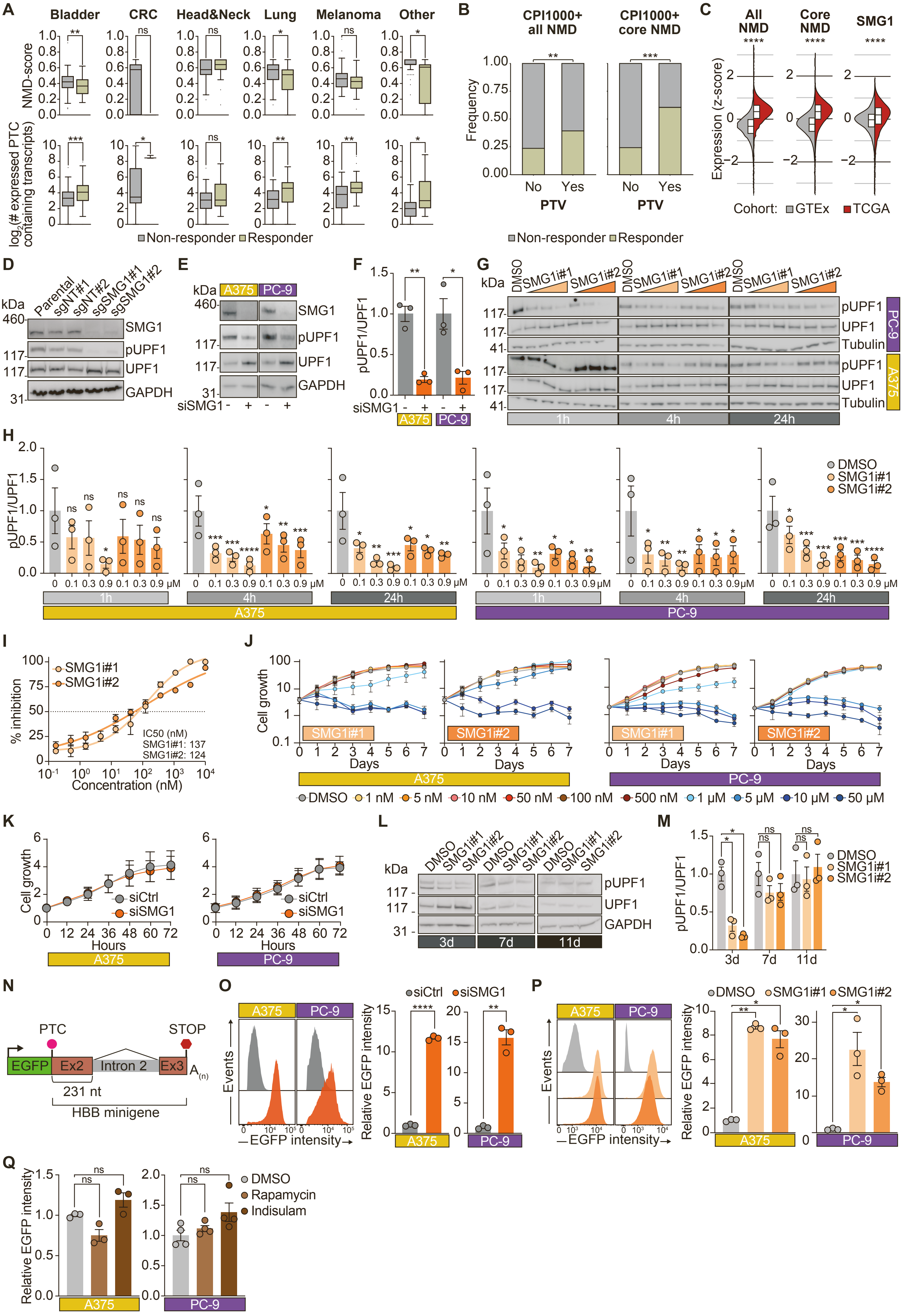

A
Bladder
CRC
Head&Neck
Lung
Melanoma
Other
NMD-score
log2(# expressed PTC containing transcripts)
Non-responder
Responder
B
CPI1000+ all NMD
CPI1000+ core NMD
Frequency
No
Yes
PTV
C
All NMD
Core NMD
SMG1
Expression (z-score)
Cohort:
GTEx
TCGA
D
kDa
Parental
sgNT#1
sgNT#2
sgSMG1#1
sgSMG1#2
SMG1
pUPF1
UPF1
GAPDH
E
A375
PC-9
siSMG1
F
pUPF1/UPF1
G
DMSO
SMG1i#1
SMG1i#2
Tubulin
1h
4h
24h
H
SMG1i#1
SMG1i#2
I
% inhibition
IC50 (nM)
SMG1i#1: 137
SMG1i#2: 124
Concentration (nM)
J
Cell growth
Days
1 nM
5 nM
10 nM
50 nM
100 nM
500 nM
1 μM
5 μM
10 μM
50 μM
K
Hours
siCtrl
siSMG1
L
3d
7d
11d
M
N
PTC
STOP
EGFP
Ex2
Intron 2
Ex3
231 nt
HBB minigene
O
Events
EGFP intensity
Relative EGFP intensity
P
Q
Rapamycin
Indisulam

Figure S1. Supporting data for Identification of SMG1 as a candidate for cancer immunotherapy, related to Figure 1

(A) Association between CPI response and either NMD-score (top) or the number of expressed PTC-containing transcripts (bottom) across the tumor types included in the CPI1000+ cohort (two-tailed Mann-Whitney U test).

(B) CPI response rates in patients' tumors with WT NMD genes versus those harboring a protein-truncating variant (PTV) in any NMD gene (all the genes included in the NMD peer-reviewed Reactome pathway database [R-HSA-927802],[61] and/or with a significant MAGeCK score from a recent genome-wide CRISPR screen for NMD-factors;[62] left) or in core NMD genes (SMG1, SMG5, SMG6, SMG7, SMG8, SMG9, UPF1, UPF2, UPF3A, and UPF3B; right) within the CPI1000+ cohort (Fisher's exact test).

(C) RNA expression (z-score) of all NMD genes (left), core NMD genes (middle), or SMG1 (right) in normal tissues (GTEx) versus tumor samples (TCGA) (two-tailed Mann-Whitney U test).

(D) Representative western blot showing SMG1, total UPF1, and phosphorylated UPF1 (pUPF1) in A375 parental cells (untransduced) or in CRISPR-Cas9-expressing cells transduced with either non-targeting control single guide RNAs (sgNT#1 and sgNT#2) or SMG1-targeting single guide RNAs (sgSMG1#1 and sgSMG1#2). GAPDH was used as a loading control.

(E) Representative western blot showing SMG1, total UPF1, and phosphorylated UPF1 (pUPF1) in A375 (left) and PC-9 (right) cells 72 h after SMG1 knockdown (siSMG1) compared with a non-targeting siRNA control (siCtrl). GAPDH was used as a loading control.

(F) Quantification of western blots described in (E) (two-tailed Student's t test, paired; $n = 3$ biological replicates).

(G) Representative western blots of total UPF1 and pUPF1 in PC-9 (top) and A375 (bottom) cells at 1, 4, and 24 h following treatment with DMSO, SMG1i#1, or SMG1i#2 at various concentrations (100, 300, 900 nM). Tubulin was used as a loading control.

(H) Quantification of western blots described in (G) (two-way ANOVA with Tukey's multiple comparisons test, comparing DMSO control with different inhibitors and concentrations; $n = 3$ biological replicates).

(I) Determination of SMG1 kinase activity inhibition and IC_{50} calculation by pUPF1 ELISA 6 h after SMG1i treatment.

(J) Growth curves of A375 (left) and PC-9 (right) cells treated with SMG1i versus DMSO ($n = 4$ biological replicates), as measured by CyQUANT Cell Proliferation Assay.

(K) Growth curves of A375 (left) and PC-9 (right) cells treated with siSMG1 or siCtrl ($n = 3$ biological replicates), as measured by IncuCyte live-cell imaging.

(L) Representative western blot showing total UPF1 and pUPF1 in A375 cells following treatment (on day 0) with SMG1i#1, SMG1i#2, or DMSO for 3, 7, or 11 days. GAPDH was used as a loading control.

(M) Quantification of pUPF1 relative to total UPF1 from western blots shown in (L). (two-way repeated-measures ANOVA with Tukey's multiple comparisons test; $n = 3$ biological replicates).

(N) Schematic representation of the EGFP-NMD reporter; nt = nucleotides.

(O and P) Representative histograms (left) and quantification (right) of EGFP fluorescence measured by flow cytometry in A375 or PC-9 cells expressing the EGFP-NMD reporter 48 h after treatment with siSMG1 relative to siCtrl (O, two-tailed Student's t test, paired; $n = 3$ biological replicates), or after treatment with SMG1#1 or SMG1i#2 relative to DMSO (P, one-way repeated-measures ANOVA with Holm-Šidák correction; $n = 3$ biological replicates).

(Q) Quantification of EGFP fluorescence measured by flow cytometry in A375 or PC-9 cells expressing the EGFP-NMD reporter 48 h after treatment with rapamycin (100 nM) or indisulam (1 μM) relative to DMSO (one-way repeated-measures ANOVA with Holm-Šidák correction; $n = 3$–4 biological replicates). Bar plots represent the mean ± SEM; for boxplots, boxes denote the interquartile range, the black line represents the median, and whiskers extend to 1.5× the interquartile range; ns, $p > 0.05$, *$p \leq 0.05$, **$p \leq 0.01$, ***$p \leq 0.001$, ****$p \leq 0.0001$.

Figure S2

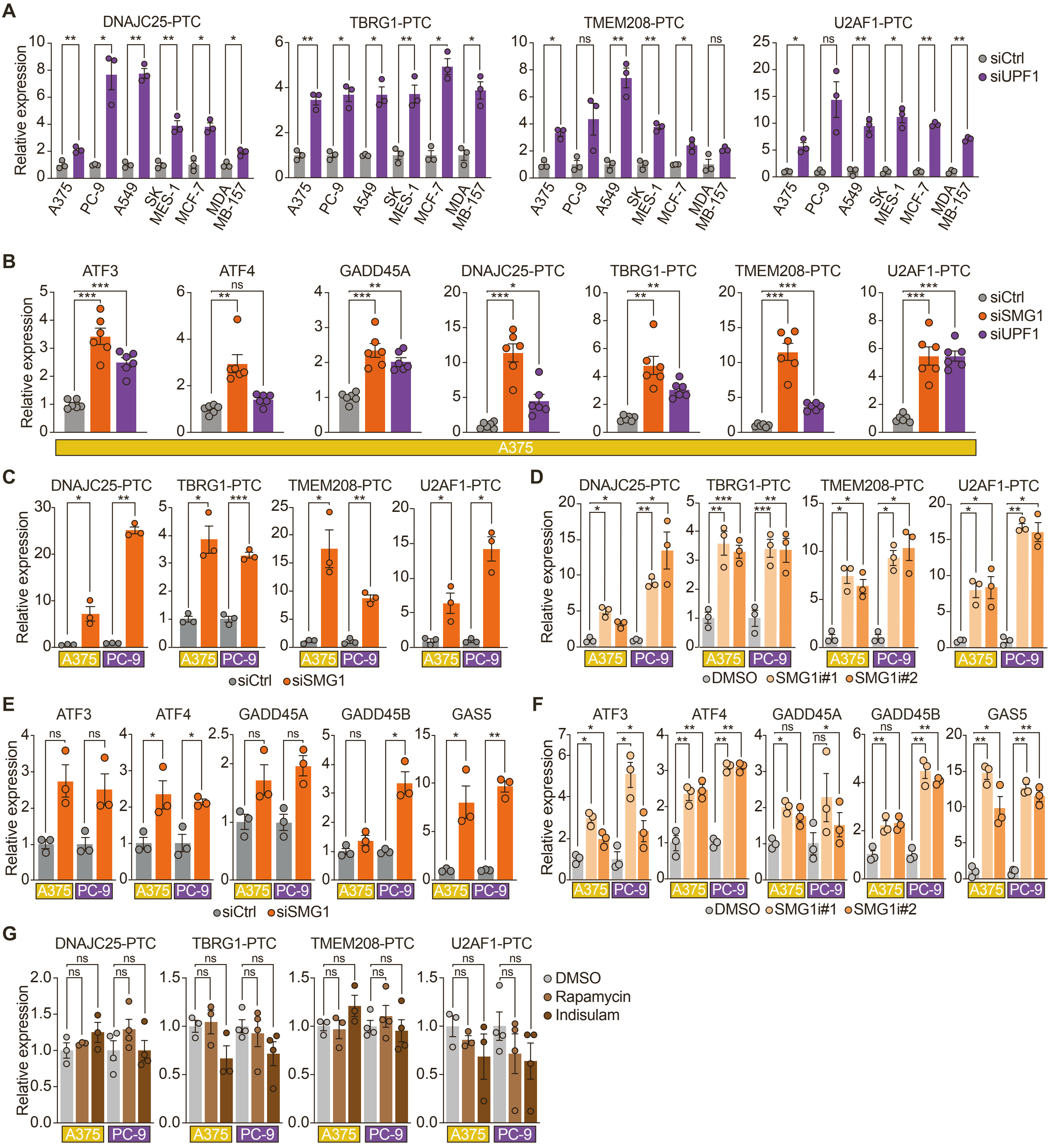
A
DNAJC25-PTC
TBRG1-PTC
TMEM208-PTC
U2AF1-PTC
Relative expression
A375
PC-9
A549
SK MES-1
MCF-7
MDA MB-157
siCtrl
siUPF1
B
ATF3
ATF4
GADD45A
DNAJC25-PTC
TBRG1-PTC
TMEM208-PTC
U2AF1-PTC
siCtrl
siSMG1
siUPF1
A375
C
siCtrl
siSMG1
D
DMSO
SMG1i#1
SMG1i#2
E
ATF3
ATF4
GADD45A
GADD45B
GAS5
siCtrl
siSMG1
F
DMSO
SMG1i#1
SMG1i#2
G
DNAJC25-PTC
TBRG1-PTC
TMEM208-PTC
U2AF1-PTC
DMSO
Rapamycin
Indisulam

Figure S2. Supporting data for Identification of SMG1 as a candidate for cancer immunotherapy, related to Figure 1

(A) Relative expression of PTC-containing transcript (DNAJC25-PTC [ENST00000447096], TBRG1-PTC [ENST00000284290], TMEM208-PTC [ENST00000562235], and U2AF1-PTC [ENST00000464750]) measured by RNA-seq in a panel of six cancer cell lines 48 h after UPF1 knockdown (siUPF1) relative to siCtrl (two-tailed Student's *t* test, paired; n = 3 biological replicates).

(B) Relative expression of canonical NMD target genes (ATF3, ATF4, and GADD45A) and selected PTC-containing transcripts (DNAJC25-PTC, TBRG1-PTC, TMEM208-PTC, and U2AF1-PTC) measured by RT-qPCR in A375 cells 72 h after treatment with siSMG1 or siUPF1 relative to siCtrl (one-way repeated-measures ANOVA with Holm-Šidák correction; n = 6 biological replicates).

(C and D) Relative expression of PTC-containing transcript measured by RNA-seq in A375 and PC-9 cells 48 h after treatment with siSMG1 relative to siCtrl (C; two-tailed Student's *t* test, paired, without multiple hypothesis correction; n = 3 biological replicates) or after treatment with SMG1i#1 or SMG1i#2 relative to DMSO (D; one-way repeated-measures ANOVA with Holm-Šidák correction; n = 3 biological replicates).

(E and F) Relative expression of canonical NMD target genes measured by RNA-seq in A375 and PC-9 cells 48 h after treatment with siSMG1 relative to siCtrl (E; two-tailed Student's *t* test, paired; n = 3 biological replicates) or after treatment with SMG1i#1 or SMG1i#2 relative to DMSO (F; one-way repeated-measures ANOVA with Holm-Šidák correction; n = 3 biological replicates).

(G) Relative expression of PTC-containing transcripts measured by RT-qPCR in A375 or PC-9 cells 48 h after treatment with rapamycin (100 nM) or indisulam (1 μM) relative to DMSO (one-way repeated-measures ANOVA with Holm-Šidák correction; $n \geq 3$ biological replicates).

Bar plots represent the mean ± SEM; ns, $p > 0.05$, *$p \leq 0.05$, **$p \leq 0.01$, ***$p \leq 0.001$.

Figure S3

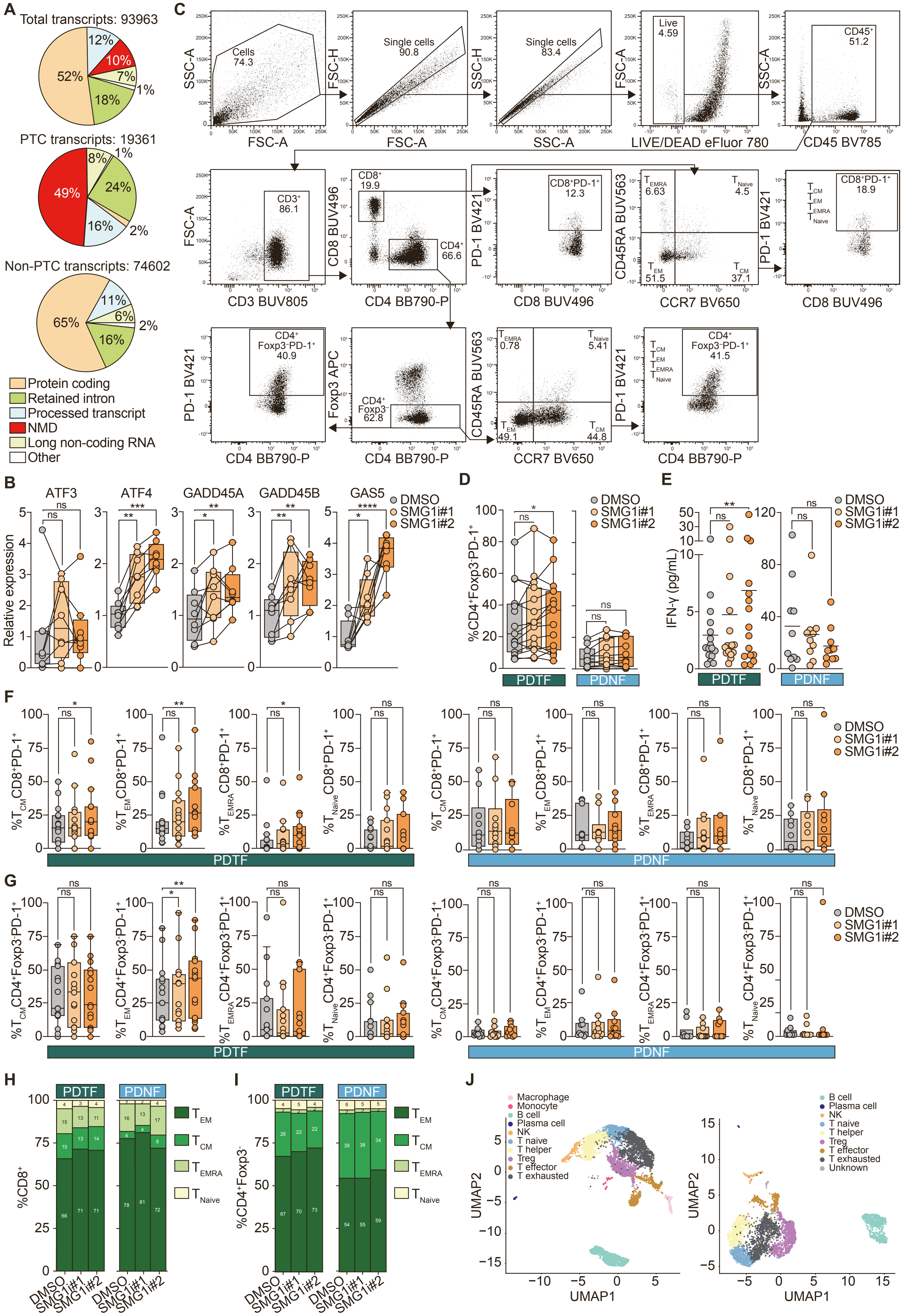
A
Total transcripts: 93963
PTC transcripts: 19361
Non-PTC transcripts: 74602
Protein coding
Retained intron
Processed transcript
NMD
Long non-coding RNA
Other
C
B
ATF3
ATF4
GADD45A
GADD45B
GAS5
Relative expression
DMSO
SMG1i#1
SMG1i#2
D
E
IFN-γ (pg/mL)
PDTF
PDNF
F
G
H
I
J
UMAP1
UMAP2

Figure S3. Supporting data for SMG1i activates tumor-specific T cells in human patient-derived fragments, related to Figure 2

(A) Pie chart showing the proportion (%) of all transcripts (top), PTC-containing transcripts (middle), and non-PTC-containing transcripts (bottom) grouped by GENCODE/Ensembl biotypes identified by RNA-seq in colorectal cancer (CRC) PDTFs 24 h after treatment with SMG1i#1, SMG1i#2, or DMSO (n = 8 patients).

(B) Relative expression of canonical NMD target genes measured by RNA-seq in CRC PDTFs 24 h after treatment with SMG1i#1 or SMG1i#2 relative to DMSO (one-way repeated-measures ANOVA with Holm-Šidák correction; n = 8 patients).

(C) Representative gating strategy of high-dimensional flow cytometry experiments on PDFs.

(D) PD-1 surface expression (% of $CD4^+Foxp3^-$ T cells) in CRC PDTFs (left; n = 15 patients) and in matched NAT PDNFs (right; n = 10 patients) 96 h after treatment with SMG1i#1, SMG1i#2, or DMSO (one-way repeated-measures ANOVA with Holm-Šidák correction).

(E) IFN-γ levels in supernatants from CRC PDTFs (left; n = 16 patients) and NAT PDNFs (right; n = 10 patients) following treatment with SMG1i#1, SMG1i#2, or DMSO (Wilcoxon matched-pairs signed-rank test).

(F and G) Percentage of PD-1 surface expression in $CD8^+$ (F) or $CD4^+Foxp3^-$ (G) T cells from CRC PDTF (left; n = 15 patients) and NAT PDNF (right; n = 10 patients described in (D), stratified by T cell subset: central memory (T_{CM}), effector memory (T_{EM}), effector memory cells re-expressing CD45RA (T_{EMRA}), and naive (T_{Naive}) (one-way repeated-measures ANOVA with Holm-Šidák correction).

(H and I) Relative abundance (%) of $CD8^+$ (H) and $CD4^+Foxp3^-$ (I) T cells described in (F) (for $CD8^+$) and in (G) (for $CD4^+Foxp3^-$).

(J) Uniform manifold approximation and projection (UMAP) plot of immune cells (colored by cell type) identified in scRNA-seq of CRC PDTFs after treatment with DMSO (left) or SMG1i#2 (right).

Bar plots represent the mean ± SEM; for boxplots, boxes denote the interquartile range, the black line indicates the median, and whiskers extend to 1.5× the interquartile range. A line connects dots corresponding to the same patient across treatments; ns, $p > 0.05$, *$p \leq 0.05$; **$p \leq 0.01$; ***$p \leq 0.001$; ****$p \leq 0.0001$.

Figure S4

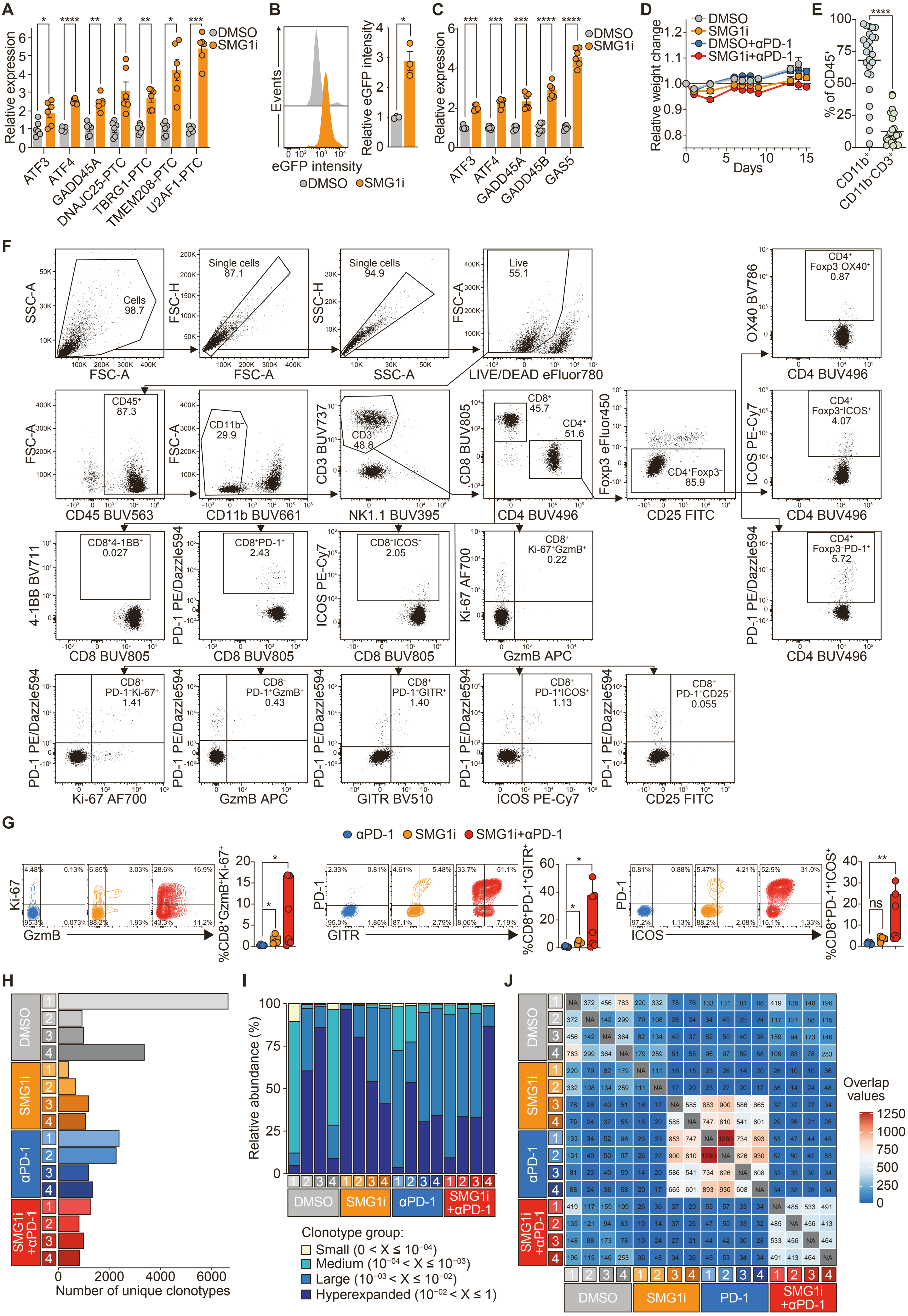
A
Relative expression
DMSO
SMG1i
ATF3
ATF4
GADD45A
DNAJC25-PTC
TBRG1-PTC
TMEM208-PTC
U2AF1-PTC
B
Events
eGFP intensity
Relative eGFP intensity
DMSO
SMG1i
C
Relative expression
ATF3
ATF4
GADD45A
GADD45B
GAS5
DMSO
SMG1i
D
Relative weight change
Days
DMSO
SMG1i
DMSO+αPD-1
SMG1i+αPD-1
E
% of CD45+
CD11b+
CD11b-CD3+
F
Cells 98.7
SSC-A
FSC-A
Single cells 87.1
FSC-H
Single cells 94.9
SSC-H
SSC-A
Live 55.1
LIVE/DEAD eFluor780
CD45+ 87.3
CD45 BUV563
CD11b- 29.9
CD11b BUV661
CD3+ 48.8
CD3 BUV737
NK1.1 BUV395
CD8+ 45.7
CD4+ 51.6
CD8 BUV805
CD4 BUV496
CD4+Foxp3- 85.9
Foxp3 eFluor450
CD25 FITC
CD4+ Foxp3-OX40+ 0.87
OX40 BV786
CD4+ Foxp3-ICOS+ 4.07
ICOS PE-Cy7
CD4+ Foxp3-PD-1+ 5.72
PD-1 PE/Dazzle594
CD8+4-1BB+ 0.027
4-1BB BV711
CD8+PD-1+ 2.43
CD8+ICOS+ 2.05
CD8+ Ki-67+GzmB+ 0.22
Ki-67 AF700
GzmB APC
CD8+ PD-1+Ki-67+ 1.41
CD8+ PD-1+GzmB+ 0.43
CD8+ PD-1+GITR+ 1.40
GITR BV510
CD8+ PD-1+ICOS+ 1.13
CD8+ PD-1+CD25+ 0.055
G
αPD-1
SMG1i
SMG1i+αPD-1
Ki-67
GzmB
%CD8+GzmB+Ki-67+
PD-1
GITR
%CD8+PD-1+GITR+
ICOS
%CD8+PD-1+ICOS+
ns
H
DMSO
SMG1i
αPD-1
SMG1i +αPD-1
Number of unique clonotypes
I
Relative abundance (%)
DMSO
SMG1i
αPD-1
SMG1i +αPD-1
Clonotype group:
Small (0 < X ≤ 10^-04)
Medium (10^-04 < X ≤ 10^-03)
Large (10^-03 < X ≤ 10^-02)
Hyperexpanded (10^-02 < X ≤ 1)
J
DMSO
SMG1i
αPD-1
PD-1
SMG1i +αPD-1
Overlap values

Figure S4. Supporting data for SMG1 inhibition potentiates tumor immunogenicity and CPI efficacy *in vivo* by enhancing T cell responses, related to Figure 3

(A) Relative expression of canonical NMD target genes and PTC-containing transcripts measured by RT-qPCR 24 h after treatment of PC-9 cells with KVS0001 (SMG1i) relative to DMSO (two-tailed Student's *t* tests, paired, with Holm-Šidák correction for multiple comparison; $n = 6$ biological replicates).

(B) Representative histograms (left) and quantification (right) of EGFP fluorescence measured by flow cytometry in PC-9 cells expressing the EGFP-NMD reporter 72 h after treatment with SMG1i relative to DMSO (two-tailed Student's *t* test, paired; $n = 3$ biological replicates).

(C) Relative expression of canonical NMD target genes measured by RT-qPCR 24 h after treatment of LLC1 cells with SMG1i relative to DMSO (two-tailed Student's *t* tests, paired, with Holm-Šidák correction for multiple comparison; $n = 6$ biological replicates).

(D) Body weight changes over time in mice treated with DMSO ($n = 9$ biological replicates), SMG1i ($n = 7$ biological replicates), αPD-1 ($n = 7$ biological replicates), or SMG1i+αPD-1 ($n = 8$ biological replicates), shown relative to day 0 (pre-treatment initiation).

(E) Quantification of $CD11b^+$ (myeloid) and $CD11b^-CD3^+$ (T cell) populations as a percentage of total $CD45^+$ immune cells in tumors described in Figure 3B ($n = 25$; Wilcoxon matched-pairs signed-rank test).

(F) Representative gating strategy of high-dimensional flow cytometry experiments on mouse xenograft tumors.

(G) Representative flow cytometry plots (left) and quantification (right) of $CD8^+$ T cells following treatment with SMG1i ($n = 4$ biological replicates), αPD-1 ($n = 5$ biological replicates), and SMG1i+αPD-1 ($n = 7$ biological replicates) (two-tailed Mann-Whitney U test).

(H) Number of unique T cell clonotypes identified by bulk TCR-seq of LLC1 tumors described in Figure 3D ($n = 4$ biological replicates).

(I) Relative abundance (%) of TCRs in tumors described in Figure 3E.

(J) TCR overlap analysis of bulk TCR-seq of tumors described in Figure 3E.

Bar plots represent the mean ± SEM; for boxplots, boxes indicate the interquartile range, the black line represents the median, and whiskers extend to 1.5× the interquartile range; ns, $p > 0.05$, *$p \leq 0.05$; **$p \leq 0.01$; ***$p \leq 0.001$; ****$p \leq 0.0001$.

Figure S5

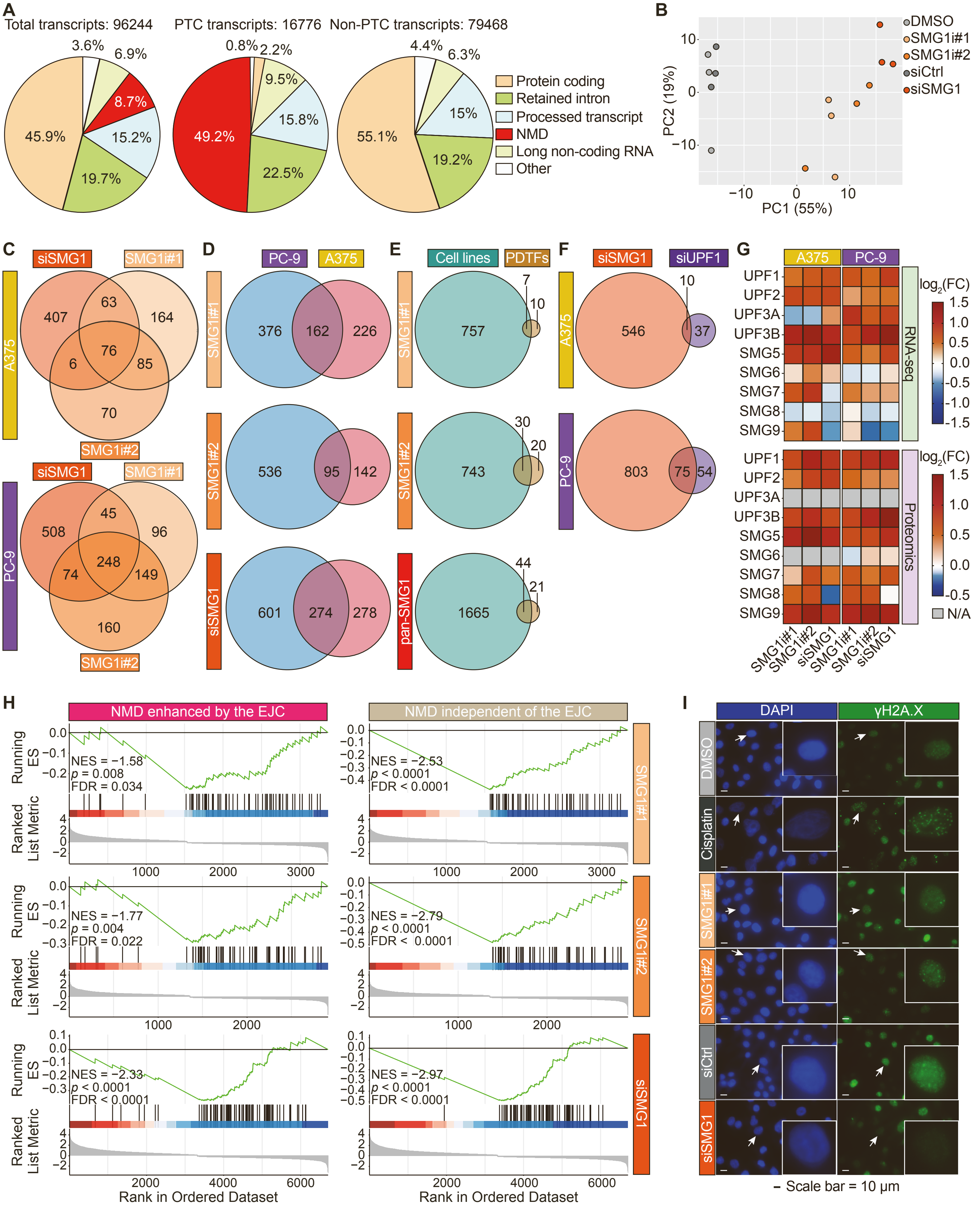

A
Total transcripts: 96244
PTC transcripts: 16776
Non-PTC transcripts: 79468
Protein coding
Retained intron
Processed transcript
NMD
Long non-coding RNA
Other
B
DMSO
SMG1i#1
SMG1i#2
siCtrl
siSMG1
PC1 (55%)
PC2 (19%)
C
D
E
F
G
log2(FC)
RNA-seq
Proteomics
N/A
H
NMD enhanced by the EJC
NMD independent of the EJC
Running ES
Ranked List Metric
Rank in Ordered Dataset
I
DAPI
γH2A.X
DMSO
Cisplatin
SMG1i#1
SMG1i#2
siCtrl
siSMG1
Scale bar = 10 μm

Figure S5. Supporting data for SMG1 inhibition promotes expression of PTC-containing isoforms encoding strong binding neoantigens, related to Figure 4

(A) Pie chart showing the proportion (%) of all transcripts (left), PTC-containing transcripts (middle), and non-PTC-containing transcripts (right) grouped by GENCODE/Ensembl biotypes identified by RNA-seq in A375 and PC-9 cells across all conditions.

(B) PCA plot of RNA-seq data for PC-9 cells as described in (A); PC: principal component.

(C) Venn diagrams illustrating the overlap of significantly upregulated PTC-containing transcripts ($q < 0.05$, rIF > 1.5) between SMG1i#1, SMG1i#2, and siSMG1 in A375 (top) and PC-9 (bottom) cells.

(D) Venn diagrams illustrating the overlap of significantly upregulated PTC-containing transcripts ($q < 0.05$, rIF > 1.5) in PC-9 (left) and A375 (right) cells following treatment with SMG1i#1 (top), SMG1i#2 (middle), or siSMG1 (bottom).

(E) Venn diagrams illustrating the overlap of significantly upregulated PTC-containing transcripts ($q < 0.05$, rIF > 1.5) between cell lines (A375 and PC-9, left) and PDTFs (right), following treatment with SMG1i#1 (top), SMG1i#2 (middle) or the intersection of SMG1i#1, SMG1i#2, and siSMG1 for cell lines and SMG1i#1 and SMG1i#2 for PDTFs (pan-SMG1, bottom).

(F) Venn diagrams illustrating the overlap of significantly upregulated PTC-containing transcripts ($q < 0.05$, rIF > 1.5) following treatment with siSMG1 or siUPF1 in A375 (top) and PC-9 (bottom) cells.

(G) Heatmaps of log_2(FC) in RNA (top) and protein (bottom) expression of core NMD genes in A375 (left) and PC-9 (right) cells following treatment with SMG1i#1 or SMG1i#2 relative to DMSO, or with siSMG1 relative to siCtrl, measured by RNA-seq and proteomics, respectively (n = 3 biological replicates).

(H) GSEA enrichment plots for NMD pathways in the Reactome database[61]: NMD enhanced by the EJC (R-HSA-975957, left) and NMD independent of the EJC (R-HSA-975956, right) from combined RNA-seq data in A375 and PC-9 cells following treatment with SMG1i#1 (top), SMG1i#2 (middle), or siSMG1 (bottom). NES: normalized enrichment score.

(I) Representative image of γH2A.X foci staining in A375 cells treated as described in Figure 4G. Scale bar = 10 μm.

Figure S6

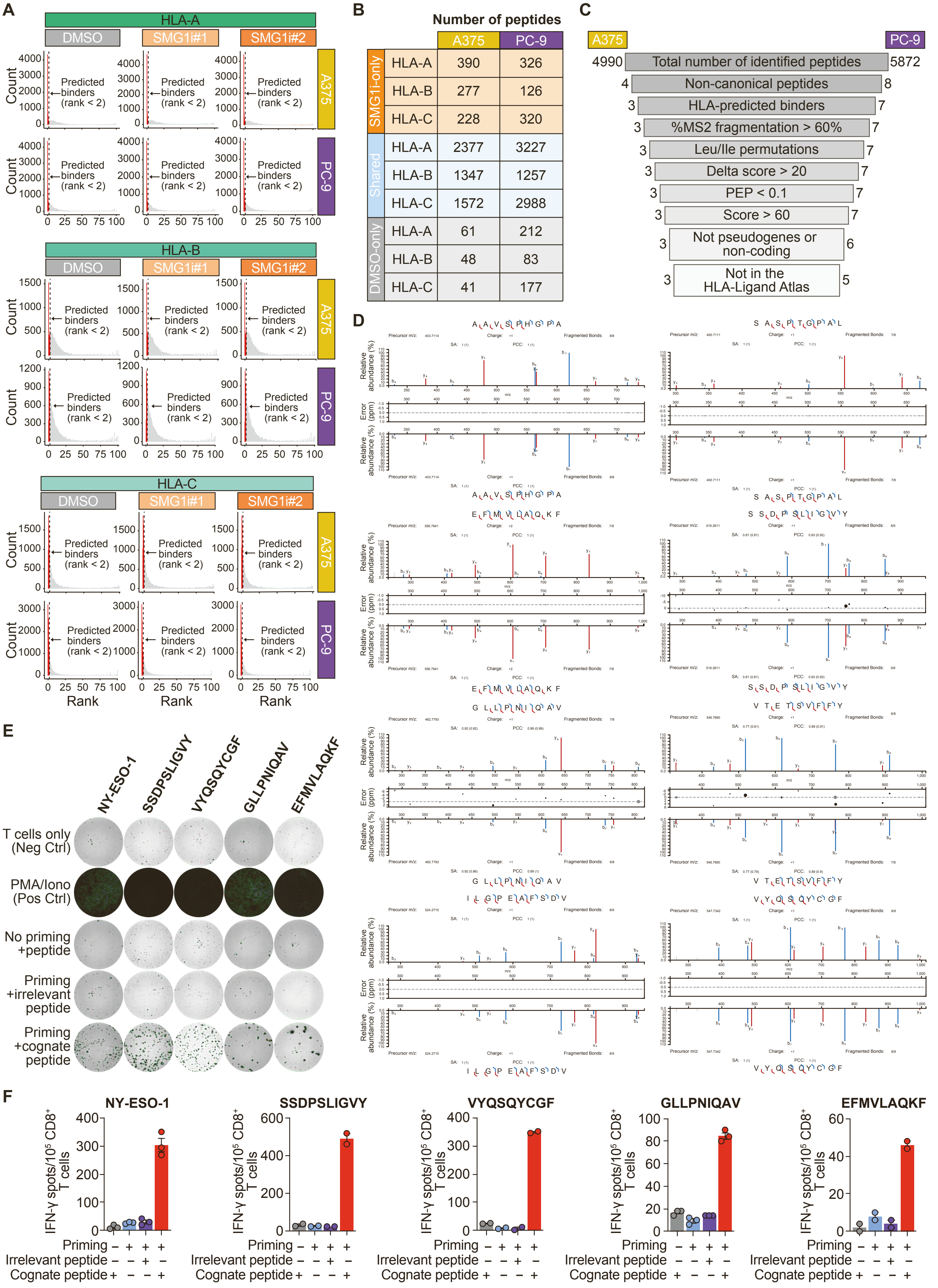
A
HLA-A
HLA-B
HLA-C
DMSO
SMG1i#1
SMG1i#2
A375
PC-9
Count
Predicted binders (rank < 2)
Rank
B
Number of peptides
		A375	PC-9
SMG1i-only	HLA-A	390	326
	HLA-B	277	126
	HLA-C	228	320
Shared	HLA-A	2377	3227
	HLA-B	1347	1257
	HLA-C	1572	2988
DMSO-only	HLA-A	61	212
	HLA-B	48	83
	HLA-C	41	177
C
A375
PC-9
4990 Total number of identified peptides 5872
4 Non-canonical peptides 8
3 HLA-predicted binders 7
3 %MS2 fragmentation > 60% 7
3 Leu/Ile permutations 7
3 Delta score > 20 7
3 PEP < 0.1 7
3 Score > 60 7
3 Not pseudogenes or non-coding 6
3 Not in the HLA-Ligand Atlas 5
D
AALVSPHGPA
SASPTGPAL
EFMVLAQKF
SSDPSLIGVY
GLLPNIQAV
VTETSVFFY
ILGPEAFSDV
VYQSQYCGF
Relative abundance (%)
Error (ppm)
E
NY-ESO-1
SSDPSLIGVY
VYQSQYCGF
GLLPNIQAV
EFMVLAQKF
T cells only (Neg Ctrl)
PMA/Iono (Pos Ctrl)
No priming +peptide
Priming +irrelevant peptide
Priming +cognate peptide
F
NY-ESO-1
SSDPSLIGVY
VYQSQYCGF
GLLPNIQAV
EFMVLAQKF
IFN-γ spots/10^5 CD8+ T cells
Priming – + + +
Irrelevant peptide – – + –
Cognate peptide + – – +

Figure S6. Supporting data for SMG1 inhibition promotes presentation of immunogenic neoantigens derived from PTC-containing transcripts via MHC-I, related to Figure 5

(A) Representative histogram showing the predicted NetMHCpan binding rank of peptides identified by HLA-A, -B, and -C immunoprecipitation followed by peptidomics from A375 and PC-9 cells treated with SMG1i or DMSO. Peptides with a rank < 2 (highlighted in red, on the left side of the dashed line) are considered predicted binders.

(B) Number of peptides identified for each HLA allele (-A, -B, and -C) in at least one replicate following treatment with SMG1i#1, SMG1i#2, or DMSO in A375 (left) and PC-9 (right) cells (*n* = 3 biological replicates).

(C) Funnel plot illustrating the filtering process used to detect aberrant peptides induced by treatment with SMG1i#1 or SMG1i#2 in the immunopeptidomics data.

(D) Mirror plots presenting the peptide spectrum matches between experimentally identified (top spectrum) and synthetic (bottom spectrum) peptides in A375 and PC-9 cells; PCC: Pearson correlation coefficient; SA: Spectrum angle; FB: Fragmented bonds.

(E) Representative ELISpot images for peptides tested in Figure 5E. T cells only and PMA (phorbol 12-myristate 13-acetate)/Iono (ionomycin) serve as a negative (Neg Ctrl) and a positive (Pos Ctrl) control, respectively. NY-ESO-1 SLLMWITQC ($NYE_{157–165}$) was used as an immunogenic antigen control.

(F) Quantification of IFN-γ ELISpot responses (spots per 10^5 $CD8^+$ T cells) from (E); NY-ESO-1 SLLMWITQC ($NYE_{157–165}$) was used as an immunogenic antigen control (*n* = 2–3 technical replicates). Bar plots represent the mean ± SEM.

Figure S7

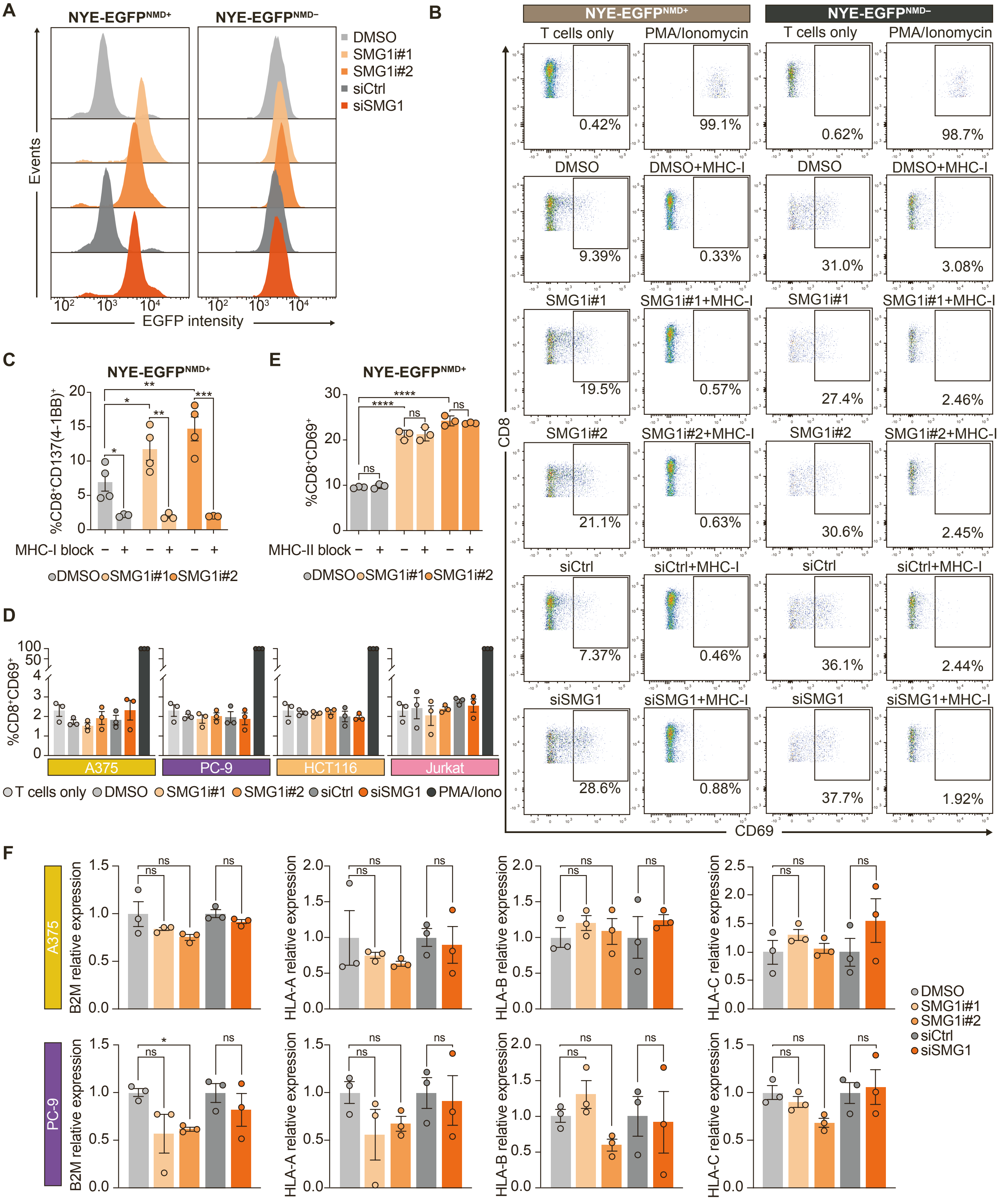

A
NYE-EGFPNMD+
NYE-EGFPNMD−
DMSO
SMG1i#1
SMG1i#2
siCtrl
siSMG1
Events
EGFP intensity
B
NYE-EGFPNMD+
NYE-EGFPNMD−
T cells only
PMA/Ionomycin
DMSO
DMSO+MHC-I
SMG1i#1
SMG1i#1+MHC-I
SMG1i#2
SMG1i#2+MHC-I
siCtrl
siCtrl+MHC-I
siSMG1
siSMG1+MHC-I
0.42%
99.1%
0.62%
98.7%
9.39%
0.33%
31.0%
3.08%
19.5%
0.57%
27.4%
2.46%
21.1%
0.63%
30.6%
2.45%
7.37%
0.46%
36.1%
2.44%
28.6%
0.88%
37.7%
1.92%
CD8
CD69
C
NYE-EGFPNMD+
%CD8+CD137(4-1BB)+
MHC-I block
DMSO SMG1i#1 SMG1i#2
E
NYE-EGFPNMD+
%CD8+CD69+
MHC-II block
DMSO SMG1i#1 SMG1i#2
D
%CD8+CD69+
A375
PC-9
HCT116
Jurkat
T cells only
DMSO
SMG1i#1
SMG1i#2
siCtrl
siSMG1
PMA/Iono
F
A375
PC-9
B2M relative expression
HLA-A relative expression
HLA-B relative expression
HLA-C relative expression
DMSO
SMG1i#1
SMG1i#2
siCtrl
siSMG1

Figure S7. Supporting data for SMG1 inhibition promotes tumor immunogenicity and T cell killing in an NMD- and antigen-dependent manner, related to Figure 6

(A) Representative histograms of EGFP fluorescence in A375 cells expressing the NYE-EGFP^{NMD+} (left) and NYE-EGFP$^{NMD–}$ (right) reporters 72 h after treatment with DMSO, SMG1i#1, SMG1i#2, siCtrl, or siSMG1.

(B) Representative gating strategy for 1G4 CD8^{+}CD69^{+} Jurkat T cells after 24 h of co-culture with A375 cells expressing the NYE-EGFP^{NMD+} (left) or NYE-EGFP$^{NMD–}$ (right) reporter. T cells only and PMA/ionomycin serve as negative and positive controls, respectively.

(C) CD137 (4-1BB) surface expression (%) in peripheral blood CD8^{+} T cells from healthy donors (HD), transduced with the 1G4 TCR and co-cultured for 24 h with LUAD PDTOs expressing the NYE-EGFP^{NMD+} reporter pre-treated for 48 h with SMG1i#1, SMG1i#2, or DMSO. Co-cultures were performed in the presence (+) or absence (–) of an MHC-I blocking antibody (two-way mixed-effects model with Tukey's multiple comparisons test; *n* = 3–4 biological replicates).

(D) CD69 surface expression (%) in 1G4 CD8^{+} Jurkat T cells co-cultured for 24 h with A375, PC-9, or HCT116 cells (not expressing the NYE-EGFP^{NMD+} or the NYE-EGFP$^{NMD–}$ reporters) pre-treated for 48 h with DMSO, SMG1i#1, SMG1i#2, siCtrl, or siSMG1. CD69 surface expression was also assessed in 1G4 CD8^{+} Jurkat T cells treated for 48 h with the same conditions, without co-culturing with cancer cells. T cells only (no co-culture) serve as a negative control; PMA/Iono-treated T cells serve as a positive control; *n* = 3 biological replicates.

(E) CD69 surface expression (%) in 1G4 CD8^{+} Jurkat T cells co-cultured for 24 h with A375 cells expressing the NYE-EGFP^{NMD+} reporter pre-treated for 48 h with SMG1i#1, SMG1i#2, or DMSO, in the presence (+) or absence (–) of an MHC class II blocking antibody (two-way repeated-measures ANOVA with Tukey's multiple comparisons test; *n* = 3 biological replicates).

(F) Relative expression of MHC class I-encoding genes measured by RNA-seq in A375 (top) and PC-9 (bottom) cells after treatment with SMG1i#1 or SMG1i#2 relative to DMSO, or with siSMG1 relative to siCtrl (one-way repeated-measures ANOVA with Holm-Šidák correction; *n* = 3 biological replicates).

Bar plots represent the mean ± SEM; ns, $p > 0.05$, *$p \leq 0.05$; ***$p \leq 0.001$; ****$p \leq 0.0001$.

Table S2. Patient characteristics, related to Figure 2

ID	Use	Gender	Age	Tumour type	MMR status	Tumour location	Stage (TNM)	Therapy	Normal tissue
CRC_000	Optimization	F	72	Moderately differentiated adenocarcinoma	Proficient	Rectal	pT2, N1b, Mx	N/A	No
CRC_001	Flow cytometry; CBA	M	70	Moderately differentiated adenocarcinoma	Proficient	Caecum	T4a, N1a	5-FU adjuvant chemotherapy	No
CRC_002	Flow cytometry; CBA	F	64	Poorly differentiated adenocarcinoma	Deficient	Caecum	pT3, pN0	N/A	Yes
CRC_003	Flow cytometry; CBA	F	62	Well differentiated adenocarcinoma	Proficient	Caecum	pT4a, pN0	N/A	Yes
CRC_004	Flow cytometry; CBA	M	50	Moderately differentiated adenocarcinoma	Proficient	Rectum	pT3, N0	6 months of adjuvant capecitabine chemotherapy	No
CRC_005	Flow cytometry; CBA	M	42	Moderately differentiated adenocarcinoma	Proficient	Rectum	pT3, N0	N/A	No
CRC_006	Flow cytometry; CBA	M	74	Moderately differentiated adenocarcinoma	Deficient	Caecum	pT3, N1c, M0	4 cycles of adjuvant CAPOX chemotherapy	No
CRC_007	CBA	M	51	Moderately differentiated adenocarcinoma	Proficient	Rectum	pT2, N0,Mx	N/A	No
CRC_008	Flow cytometry; CBA; RNA-seq	F	58	Well differentiated adenocarcinoma	Deficient	Rectum	ypT4a, ypN1	6 weeks XRT prior to resection followed by 6 months of adjuvant FOLFOX after resection	No
CRC_009	Flow cytometry; CBA	M	58	Moderately differentiated adenocarcinoma	Proficient	Ascending colon	pT4a, N2a, Mx	6 months of adjuvant 2 weekly FOLFOX	Yes

CRC_010	Flow cytometry; CBA	F	90	Moderately differentiated adenocarcinoma	Proficient	Ascending colon	pT4a, N2b, M1c	Death due to bronchopneumonia	Yes
CRC_011	Flow cytometry; CBA; RNA-seq	M	80	Moderately differentiated adenocarcinoma	Proficient	Rectum	pT1, N0, M0	N/A	No
CRC_012	Flow cytometry; CBA; RNA-seq	F	69	Moderately differentiated adenocarcinoma	Proficient	Rectum	pT4a, N1c, Mx	6 months of adjuvant FOLFOX	Yes
CRC_013	Flow cytometry; CBA; RNA-seq	M	88	Moderately differentiated adenocarcinoma	Proficient	Caecum	pT3, N0, Mx	N/A	Yes
CRC_014	Flow cytometry; CBA; RNA-seq	M	64	Moderately differentiated adenocarcinoma	Proficient	Ascending colon	pT3, N1a, Mx	N/A	Yes
CRC_015	Flow cytometry; CBA	F	74	Moderately differentiated adenocarcinoma	Proficient	Colon	pT2, pN0, pMx	N/A	Yes
CRC_016	Flow cytometry; CBA; RNA-seq	M	78	Moderately differentiated adenocarcinoma	Proficient	Colon	T3, N1b	Radical chemoradiation with rectal boost (2007). Adjuvant FOLFOX (5-FU single agent for first cycle) (2023)	Yes
CRC_017	RNA-seq	M	59	Adenocarcinoma	Proficient	Sigmoid colon	T3, N1b, M0	3 months adjuvant CAPOX	No
CRC_018	RNA-seq	F	77	Adenocarcinoma	Proficient	Transverse colon	pT4b, N1c, Mx	N/A	No
CRC_019	Flow cytometry; CBA	F	62	Adenocarcinoma	Deficient	Rectum	pT3, N1c	Neoadjuvant CAPOX for liver metastatic disease	Yes

Table S3. Markers used to determine the identity of T cell clusters, related to Figure 2

Treatment	Cluster ID	Annotation	Marker
DMSO	0	$CD4^+$ T cells	IL7R
	1	Treg	FOXP3, IL2RA
	2	Exhausted $CD8^+$ T cells	CXCL13, TIGIT
	3	Naive T cells	TCF7, CCR7
	4	B cells	CD79A, MS4A1
	5	Exhausted $CD8^+$ T cells	CXCL13, LAG3
	6	B cells	IGHM, CD79A, MS4A1
	7	Cytotoxic $CD8^+$ T cells	CD8A, GZMA
	8	Cytotoxic $CD8^+$ T cells	CD8A, GZMK, GZMH
	9	NK	KLRC3, KLRD1
	10	Macrophage	TYROBP, FCER1G
	11	Monocyte	CST3
	12	Plasma cell	JCHAIN, IGKC
SMG1i#2	0	Treg	FOXP3, IL2RA
	1	$CD4^+$ T cells	IL7R
	2	Naive T cells	TCF7, CCR7
	3	B cells	CD79A
	4	Exhausted $CD8^+$ T cells	CXCL13
	5	Exhausted $CD8^+$ T cells	ENTPD1, LAG3, CXCL13
	6	B cells	MS4A1, CD79A
	7	Cytotoxic $CD8^+$ T cells	CD8A, GZMA
	8	Cytotoxic $CD8^+$ T cells	CD8A, CD8B, GZMK, GZMH
	9	NK	GNLY, NKG7
	10	Plasma cell	JCHAIN, IGKC

Table S5. List of primers and TCR sequences used in this study, related to STAR★ Methods

Primer	Forward (FWD) primer sequence	Reverse (REV) primer sequence
Human_DNAJC25-PTC	CTACCACCCTGACCGCTACC	TGCAGGAGGACTTGAGTGTC
Human_TBRG1-PTC	ACAACAAACTGGAAGATCATCACC	CACTTCTGGTCTGGGCACTT
Human_TMEM208-PTC	GCCAGCCCAGGCACCTTA	CCAGAAGCCAGAAGGACCAG
Human_U2AF1-PTC	CCCAAAACAGTGCACAGACG	CGTCAGCAGACTGGGAAGAG
Human_ATF3	GCCATTGGAGAGCTGTCTTC	GGG CCATCTGGAACATAAGA
Human_ATF4	TTAAGCCATGGCGCTTCTCA	CTAGGCTTTCTTCAGCCCCC
Human_GADD45A	CAGAAGACCGAAAGGATGGA	ATCTCTGTCGTCGTCCTCGT
Human_GADD45B	TGACAACGACATCAACATC	GTGACCAGAGACAATGCAG
Human_GAS5	TCCTGTGAGGTATGGTGCTG	CTTCCTTGGGGACACAACTG
Human_HPRT	AGCCAGACTTTGTTGGATT TG	TTTACTGGCGATGTCAATAAG
Human_TBP	CGGCTGTTTAACTTCGCTTC	CACACGCCAAGAAACAGTGA
Human_UBC	ATTTGGGTCGCGGTTCTTG	TGCCTTGACATTCTCGATGGT
Mouse_ATF3	GGCAGGAGCATCCTTTGTCT	TGCTTTGCATAGGACCCCAG
Mouse_ATF4	CACAACATGACCGAGATGAG	CGAAGTCAAACTCTTTCAGATCC
Mouse_GADD45A	TGGAGGAAGTGCTCAGCAAG	CCACGTTATCGGGGTCTACG
Mouse_GADD45B	CCTCCTGGTCACGAACTGTC	TGGGTCTCAGCGTTCCTCTA
Mouse_GAS5	TTTCCGGTCCTTCATTCTGA	TCTTCTATTTGAGCCTCCATCCA
Mouse_HPRT	CAAACTTTGCTTTCCCTGGT	TCTGGCCTGTATCCAACACTTC
Mouse_RER1	GCCTTGGGAATTTACCACCT	CTTCGAATGAAGGGACGAAA
Mouse_RPL13A	ATGACAAGAAAAAGCGGATG	CTTTTCTGCCTGTTTCCGTA
CRISPR-V2 seq	ATTTCTTGGGTAGTTTGCAG	CAATTCCCACTCCTTTCAAG

sgRNA	Forward (FWD) guide sequence	Reverse (REV) guide sequence
sgNT_1 (non-targeting Ctrl)	CACCGATCGTTTCCGCTTAACG GCG	AAACCGCCGTTAAGCGGAAACGA TC
sgNT_2 (non-targeting Ctrl)	CACCGTAGAGATATCCGATCGT GGT	AAACACCACGATCGGATATCTCTA C
sgNSMG1_1	CACCGTAGTGTCGAGCATGGAA CCG	AAACCGGTTCCATGCTCGACACTA C
sgNSMG1_1	CACCGCATGTTAAGTCCGGATC CGA	AAACTCGGATCCGGACTTAACATG C

TCR	DNA sequence	Protein sequence
NY-ESO-1_A*02_1G4 _TCR-Vα	AAGCAAGAAGTGACACAGATCCCTGCCGCTCTGTCTGTG CCTGAGGGCGAAAACCTGGTGCTGAACTGCAGCTTCAC CGACAGCGCCATCTACAACCTGCAGTGGTTCAGACAGG ACCCCGGCAAGGGACTGACAAGCCTGCTGCTGATTCAG AGCAGCCAGAGAGAGCAGACCAGCGGCAGACTGAATGC CAGCCTGGATAAGTCCTCCGGCAGAAGCACCCTGTATAT CGCCGCTTCTCAGCCTGGCGATAGCGCCACATATCTGTG TGCCGTGCGACCTCTGTACGGCGGCAGCTACATCCCTAC ATTTGGCAGAGGCACCAGCCTGATCGTGCACCCCTAC	KQEVTQIPAALSVPEGE NLVLNCSFTDSAIYNLQ WFRQDPGKGLTSLLLIQ SSQREQTSGRLNASLDK SSGRSTLYIAASQPGDS ATYLCAVRPLYGGSYIPT FGRGTSLIVHPY

NY-ESO-1_A*02_1G4_TCR-Vβ	GGCGTGACCCAGACACCTAAGTTCCAGGTGCTGAAAACCGGCCAGAGCATGACCCTGCAGTGCGCCCAGGATATGAACCACGAGTACATGAGCTGGTACAGACAGGACCCTGGCATGGGCCTGAGACTGATCCACTATTCTGTCGGAGCCGGCATCACCGACCAGGGCGAAGTTCCTAATGGCTACAACGTGTCCAGAAGCACCACCGAGGACTTCCCACTGAGACTGCTGTCTGCCGCTCCTAGCCAGACCAGCGTGTACTTTTGTGCCAGCAGCTACGTGGGCAACACCGGCGAGCTGTTTTTTGGCGAGGGCAGCAGACTGACCGTGCTC	GVTQTPKFQVLKTGQSMTLQCAQDMNHEYMSWYRQDPGMGLRLIHYSVGAGITDQGEVPNGYNVSRSTTEDFPLRLLSAAPSQTSVYFCASSYVGNTGELFFGEGSRLTVL
